# Phosphine-Promoted Synthesis of Naphthoquinones Fused with Cyclopentadienyl Moiety Via Ring Expansion: Synthesis, Reactivity, and Ring Contraction Via [1,5] Sigmatropic Rearrangement

**DOI:** 10.1021/acs.orglett.4c03052

**Published:** 2024-10-07

**Authors:** Wei-Qing Wang, Sureshbabu Nallapati, Chun-Yu Chen, Tomoya Yaoita, Shuri Yamaoka, Michihisa Murata, Shih-Ching Chuang

**Affiliations:** † Department of Applied Chemistry, National Yang Ming Chiao Tung University, Hsinchu 30010, Taiwan; ‡ Department of Applied Chemistry, Osaka Institute of Technology, Osaka 535-8585, Japan

## Abstract

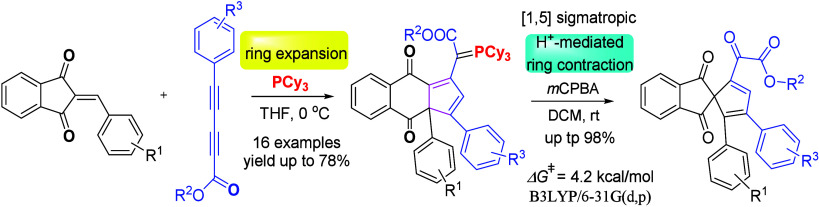

Phosphine-promoted unprecedented [3 + 2] annulation reactions via ring expansion by using 2-benzylidene-indane-1,3-diones and diynoates for the synthesis of biologically interesting novel naphthoquinones fused with a five-membered ring bearing a phosphorus ylide up to 78% yield are described. Further ring contraction through [1,5] sigmatropic rearrangement to the spiro indan-1,3-diones by *m*CPBA oxidation was revealed and inferred through oxidation, followed by protonation. The relevant structures were confirmed by single-crystal X-ray diffraction. Electrochemical studies show that the naphthoquinones and lactones with phosphorus ylides could be applied to redox colorimetric materials.

Naphthoquinone frameworks are important structural cores and exhibit a wide range of biological properties,^1^ which include anticancer, antibacterial, antifungal, antiviral, antimalarial, fungicidal, and anti-inflammatory activities. The quinone moiety is also present in many natural products,^2^ for example, Utahmycin B, 3-methyl-1*H*-benzo[*f*]indole-4,9-dione, and crassiflorone (Figure 1). Therefore, a wide range of processes have been developed for the synthesis of naphthoquinone with diverse substitution patterns.^3^

**Figure 1:**
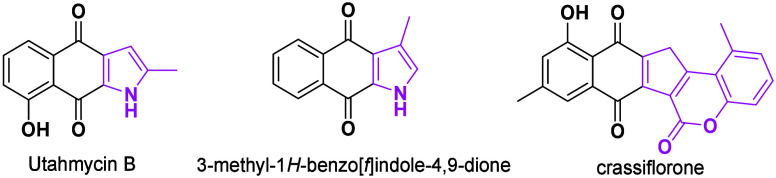
Selected examples of naturally occurring compounds containing a naphthoquinone skeleton.

Furthermore, a ring-expansion strategy is a useful tool for organic chemists to construct macromolecules of various biologically active natural products and essential drug molecules which are difficult-to-prepare in other methods.^4^ It has attracted much attention from the synthetic community due to its efficiency toward the construction of important carbocyclic compounds in the area of drug discovery. Our group has passions on unusual α-addition reactions, which have been quite rare in traditional organophosphine chemistry.^5^ In this context, we have successfully developed several phosphine-promoted multicomponent reactions.^6^

Xu reported the construction of benzo[*f*]indole-4,9-diones by silver-catalyzed tandem reaction of tosylmethyl isocyanide (TosMIC) and 2-methyleneindene-1,3-diones via a ring expansion strategy (Scheme 1a).^7^ In another report, other substituted benzo[*f*]indole-4,9-diones were obtained from indanones, aldehydes, and TosMIC under copper catalysis in one pot (Scheme 1b).^8^ Herein, we report a phosphine-promoted ring expansion by using simple starting materials 2-arylideneindan-1,3-diones and diyneoates for the synthesis of a new naphthoquinone fused with a five-membered carbocycle bearing a quaternary carbon with good yields (Scheme 1c) and studies of its ring contraction through [1,5] sigmatropic rearrangement.

**Scheme 1:**
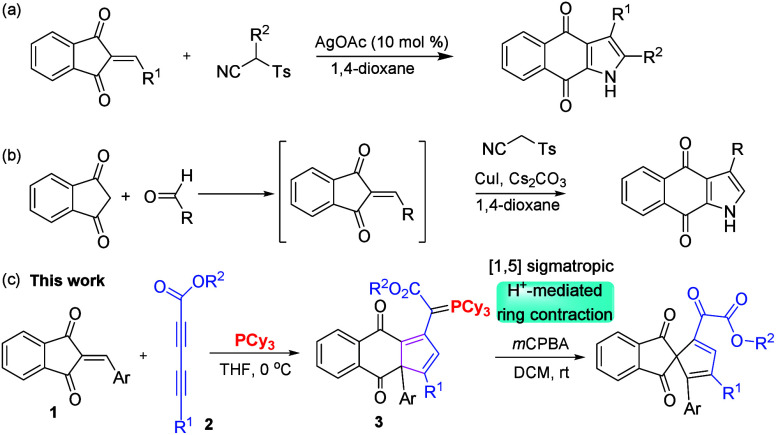
Different Synthetic Strategies for Ring Extension

We first synthesized various substituted 2-arylideneindane-1,3-diones **1** and diynoates **2** and selected **1f** and **2a** as model substrates for condition optimization by employing various solvents (Table 1). To optimize the conditions for the reaction, in an initial experiment, **1f** (1.0 equiv) was reacted with diynoate **2a** (1.2 equiv) in the presence of PCy_3_ (1.2 equiv) in THF at 0 °C for 12 h to afford **3f** in 48% yield (entry 1). We further increased the reaction time to 24 h and also increased solvent volume and found the yield of **3f** slightly increased (entries 2–3). Further increase of the molar ratios of **2a** and PCy_3_ with respect to **1f** gave higher yields 67% and 60%, respectively, for ratios of 1:1.5:1.2 and 1:1.5:1.5 (entries 4 and 5). A reaction with a higher loading of **2a** did not improve the yield (entry 6). We next screened the effect of solvents with reactions in chlorinated solvents, such as *o*-dichlorobenzene benzene (*o*-DCB), but did not observe desired product **3f** (entry 7); that in the nonchlorinated aromatic solvents, such as toluene (entry 8), gave 34% yield. Moreover, those in benzene (entry 9) or 1,4-dioxane did not give the desired product **3f** (entry 10). A more nucleophilic phosphine PBu_3_ also did not yield the products. Among all the optimized conditions, entry 4 was proved to be efficient and is the optimal condition for the synthesis of compound **3f**.

**Table 1:**
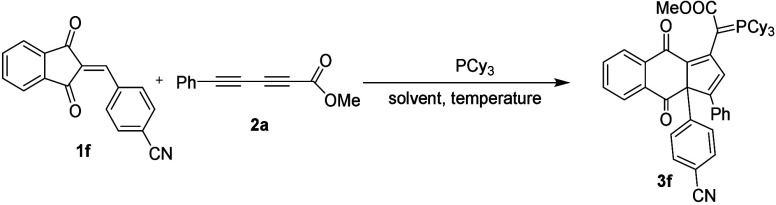
Optimization of Reaction Condition^a^

Entry	PCy_3_ (equiv)	**2a** (equiv)	Solvent (mL)	°C	Time (h)	Yield^b^ (%)
1	1.2	1.2	THF (5)	0	12	48
2	1.2	1.2	THF (5)	0	24	56
3	1.2	1.2	THF (10)	0	24	58
4^c^	1.2	1.5	THF (10)	0	24	67
5^c^	1.5	1.5	THF (10)	0	24	43
6	1.2	1.2	THF (5)	rt	12	41
7	1.2	1.2	*o*-DCB (5)	rt	12	ND
8	1.2	1.2	Toluene (5)	rt	12	34
9	1.2	1.2	Benzene (5)	rt	12	ND
10	1.2	1.2	1,4-dioxane (5)	rt	12	ND
11^d^	1.2	1.5	THF (10)	0	24	ND

a Reaction was carried out under anhydrous conditions unless otherwise noted.

b Yields were determined by ^1^H NMR spectroscopy with mesitylene as an internal standard.

c Reaction was carried out via slow addition of **2a** in 30 min.

d PBu_3_ was used.

We used the best conditions (entry 4, Table 1) to develop the scope of this reaction. Variously substituted asymmetric diynoates and various 2-arylideneindane-1,3-diones were successfully employed in the reaction to efficiently synthesize functionalized 2-(4,9-dioxo-3-phenyl-3a,9-dihydro-4*H*-cyclopenta[*b*]naphthalen-1-yl)-2-(tricyclohexyl-λ^5^-phosphanylidenes) acetate **3** in moderate to good yields. The positions of the substituents on the aromatic ring were shown to have little influence on the efficiency of this reaction. As can be seen in Scheme 2, the electron-withdrawing substituents R^1^, with *para*-substitution of bromo, chloro, and fluoro, afforded the desired products in moderate to good yields (**3c**, **3d**, **3e**, **3j**, and **3k**), and those with cyano and nitro groups at the *para* position also provided corresponding products (**3f**, **3g**, **3l**, **3n**, and **3o**) in moderate to good yields. Moreover, those containing electron-neutral and donating groups, such as *para* methyl and *meta* methoxy, were also tolerated and provided desired products (**3a**, **3b**, **3h**) in moderate yields. Furthermore, a reaction with 2-naphthyl was also successful with **2a**, resulting in a yield of 66% for the corresponding products **3i**. Last, 2-benzylidene-5,6-dichloro-1*H*-indene-1,3(2*H*)-dione reacted with **2a** under the optimized condition to give the target molecule **3p** in 64% yield. We carried out reactions with heteroaryl substituent 2-thienyl and noted that the corresponding product decomposed immediately upon isolation.

**Scheme 2:**
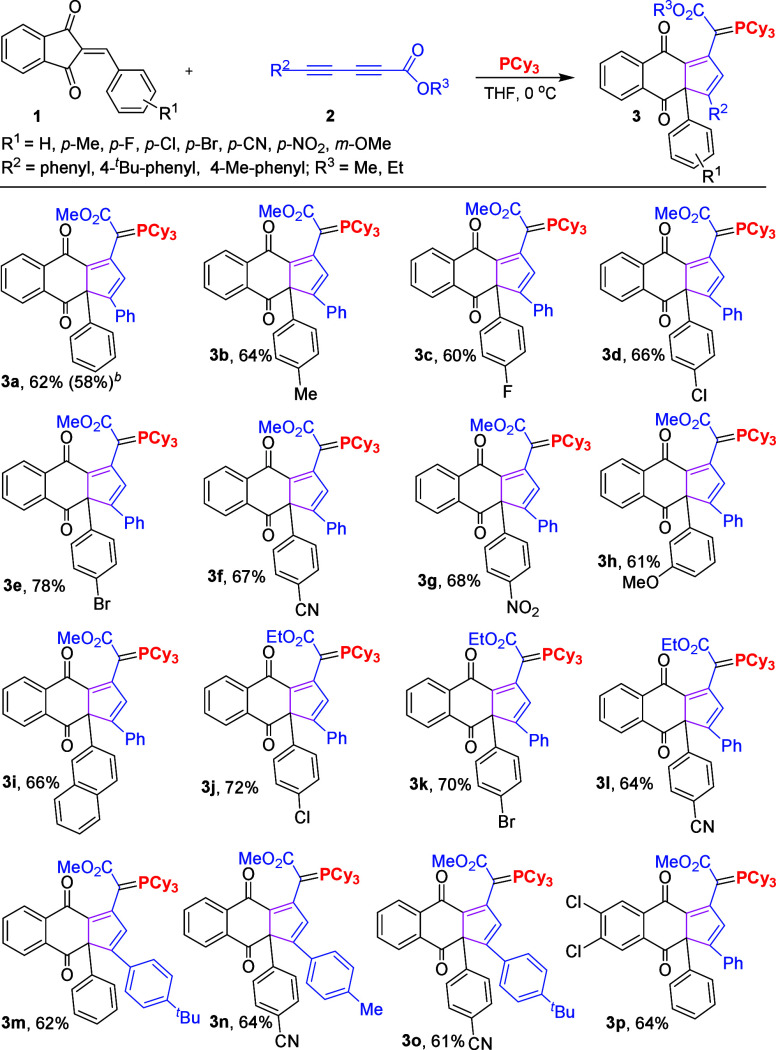
Study of the Reaction Scope **1** (0.20 mmol), **2** (0.30 mmol), and PCy_3_ (0.24 mmol) in anhydrous THF (10 mL) at 0 °C for 24 h. 1.0 mmol scale reaction.

The possible mechanism (Scheme 3) for formation of ylide **3** is proposed through initial nucleophilic attack of phosphine PCy_3_ at the α-carbon of diynoates **2** to generate reactive zwitterionic species **A** and to a resonance-derived cumulated trienoate intermediate **A**^**’**^, followed by the addition to arylidene-1,3-indanedione **1** that results in formation of the adduct intermediate **B**. Next, a proton transfer occurred to give **C**, and this was followed by intramolecular nucleophilic addition of the carbanion to the carbonyl group of the arylidene-1,3-indanedione, affording intermediate **D**. The reopening of cyclopropanoxide **D** undergoes addition to the trienoate moiety, leading to the newly generated carbanion **E**. The dione **3** is formed after proton transfer and resonance.

**Scheme 3:**
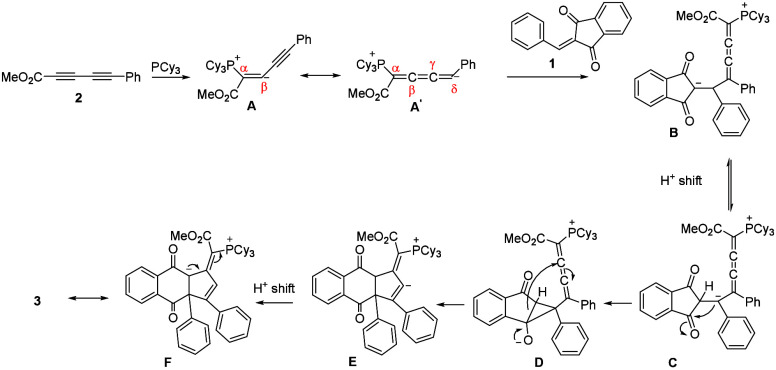
Proposed Mechanism for the Formation of Compound 3

Due to their chemical reactivity toward oxidation, the selected compounds **3a**, **3l**, and **3o** were oxidized by *m*CPBA (2.4 equiv) to give α-keto esters **4a**–**c**, respectively, in excellent yields (Scheme 4). We found that ring contraction took place through a [1,5] sigmatropic rearrangement, giving unexpected spiro species **4**. A Density Functional Theory (DFT) study showed that the direct ring contraction from **3a** with an ylide moiety to **4aPCy**_**3**_ through transition state **TS1** is unlikely to take place due to its higher Δ*G*^‡^ of 28.9 kcal mol^–1^ (Figure 2) or via the pathway through ring contraction immediately after oxidation from **3aO** to **4a** through **TS2** (Δ*G*^‡^ of 17.2 kcal mol^–1^). The most probable pathway would be the spontaneous ring contraction from protonated **3aOH** through **TS3** (Δ*G*^‡^ of 4.2 kcal mol^–1^) to **4aH** at room temperature. The proton source came from the reagent *m*CPBA used for oxidation of the ylide moiety to the keto group.

**Scheme 4:**
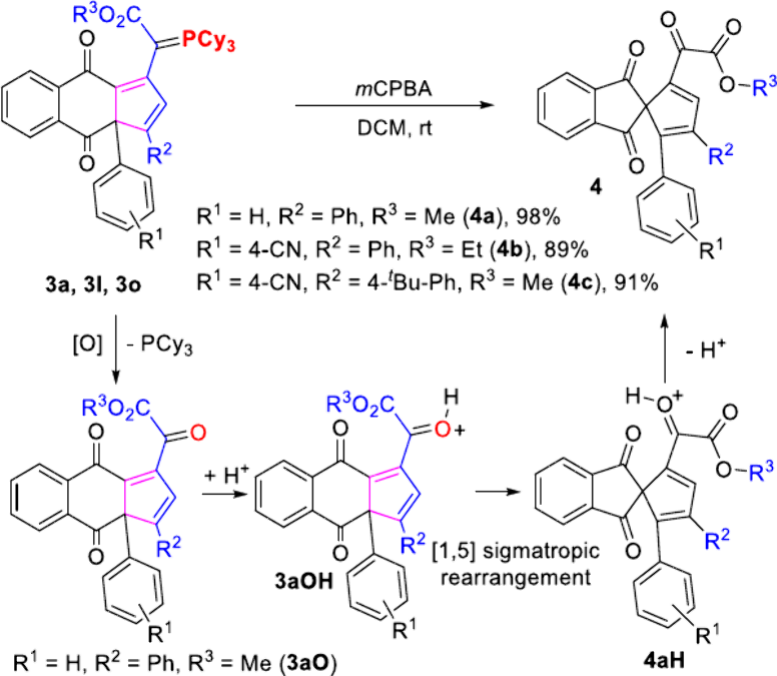
Oxidation of Naphthoquinone Ylides **3** by *m*CPBA and Ring Contraction

**Figure 2:**
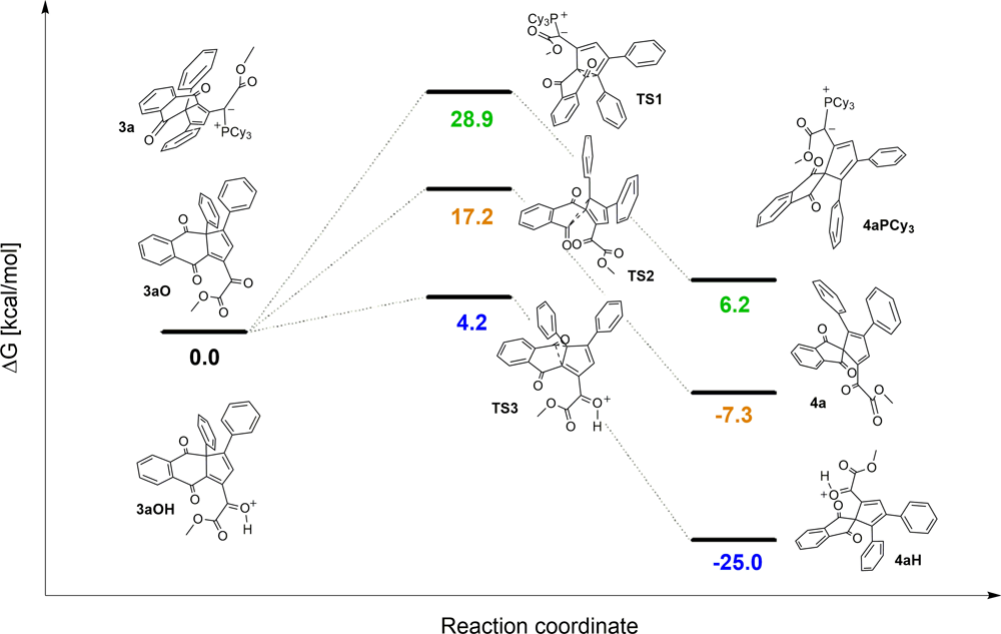
Energy profile for the ring contraction through [1,5] sigmatropic rearrangement calculated at the B3LYP/6-31G(d,p) level of theory.

These isolated compounds were characterized by using ^1^H and ^13^C nuclear magnetic resonance (NMR), electron-spray mass spectrometry (ESI), and infrared (IR) spectroscopy. We observed that these compounds with a PCy_3_ ylide moiety decomposed gradually, and immediate isolation and rapid characterization were necessary. But they could be stored in solvents pretreated with aqueous sodium bicarbonate. Further, the isolated compounds **3g**, **3h**, and **4a** tended to crystallize upon slow evaporation of their dichloromethane or chloroform solutions. We showed the single-crystal X-ray structures of compounds **3h** and **4a** (Figure 3). The pyramidalization angle (*θ*_*p*_) on C8 of **3h** is calculated to be 21.2°.

**Figure 3:**
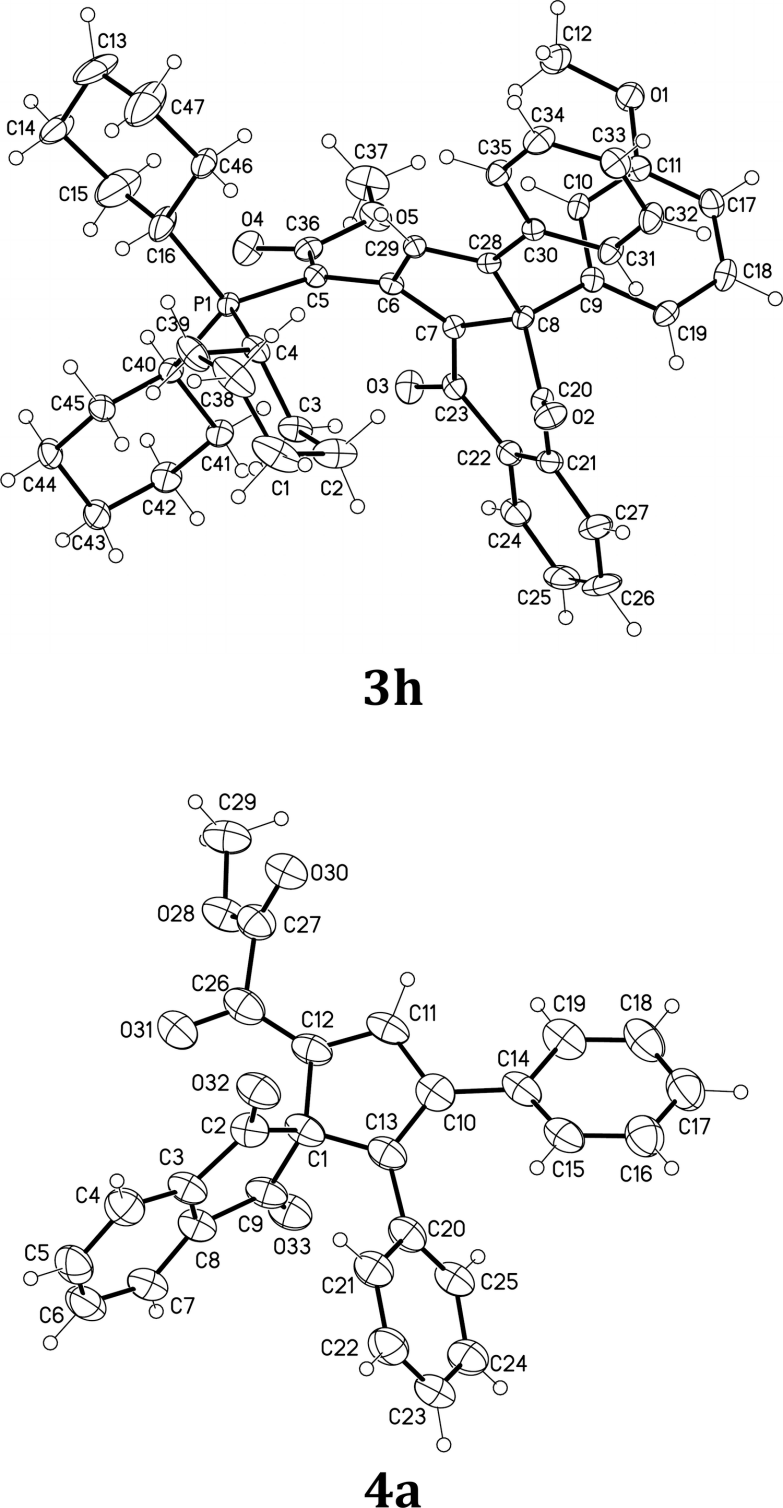
ORTEP of single-crystal X-ray structure of compounds **3h** and **4a**.

We summarized the electrochemical properties of naphthoquinone ylide **3a**, spiro indan-1,3-dione **4b**, together with naphthoquinone (**NQ**), **MTPA**, as well as γ-lactone ylide **5a** and **5b**, in Table 2. The **NQ** displayed its half-wave reduction potential (*E*_1/2_^red^) at −1.14 V and LUMO energy level of −3.25 eV. Compared to **NQ**, the *E*_1/2_^red^ of **3a**, **4a**, **5a**, and **5b** are −2.01, −1.49, −1.51, and −1.54, respectively, and that of **MTPA** was not observed. This notion indicated that naphthoquinone with a fused five-membered cyclopentadienyl and an ylide moiety exhibited a higher LUMO energy level (−2.38 eV) and compounds **4a**, **5a**, and **5b** with lower LUMO are relatively easy to be reduced. It is interesting to observe half-wave oxidation potential at 0.30, 0.66, 0.28, and 0.26 V for compounds **3a**, **MTPA**, and **5a**–**b** with a phosphorus ylide moiety, featuring its one-electron oxidation at the carbanion bound to the phosphorus. Hence, the typical compound, **MTPA**, without a naphthoquinone moiety, displays a lower-lying HOMO energy level, compared to those of **3a** and **5a**–**b**. Derived from the energy levels of frontier molecular orbitals (FMOs), **3a** shows a larger energy gap, Δ*E*_HOMO–LUMO_ of 2.31 eV, compared to those of **5a**–**b**, 1.79 and 1.80 eV, respectively. These FMOs imply that compounds **3a**, **5a**, and **5b** could be applied toward redox colorimetric materials.

**Table 2:**
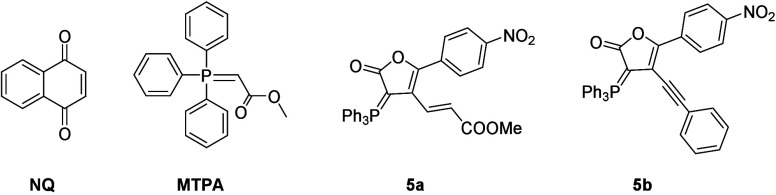
Summarized electrochemical properties of representative substrates **NQ**, **MTPA**, **3a**, **4a**, **5a**, and **5b** in 0.1 M ^*n*^Bu_4_NPF_6_ in DCM at 23 °C

Compound	*E*_1/2_^red^^a^	*E*_1/2_^ox^^a^	LUMO (eV)^b^	HOMO (eV)^b^	Δ*E*_HOMO–LUMO_ (eV)^b^
**NQ**	–1.14	-	–3.25	-	-
**3a**	–2.01	0.30	–2.38	–4.69	2.31
**4a**	–1.49	-	–2.90	-	-
**MTPA**	-	0.66	-	–5.05	-
**5a**(6b)	–1.51	0.28	–2.88	–4.67	1.79
**5b**(6e)	–1.54	0.26	–2.85	–4.65	1.80

a Reduction and oxidation half-wave potentials determined by CV measurement.

b LUMO and HOMO were estimated by the equations: LUMO = −(*E*_1/2_^red^ + 4.39), HOMO = −(*E*_1/2_^ox^ + 4.39), respectively.

The intense reddish color of **3a**, and the orange-red color **5a**–**b**, are consistent with their features of absorption at 503, 487, and 493 nm, respectively. Interestingly, the spiro indandione **4a** shows an absorption at 389 nm, due to its less structural π-conjugation compared to **3a**. The typical compound, **MTPA**, exhibits featureless absorption for its absence of naphthoquinone fused with a cyclopentadienyl moiety (Figure 4).

**Figure 4:**
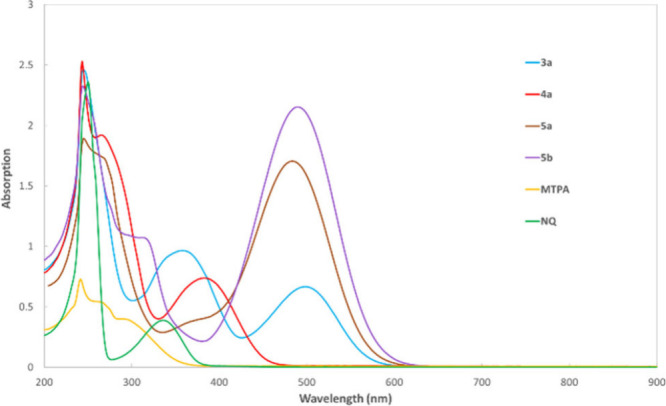
UV–vis absorption spectra of **NQ**, **MTPA**, **3a**, **4a**, **5a**, and **5b** in CHCl_3_ (1.0 × 10^–4^ M).

In summary, we have developed a novel tricyclohexyl phosphine-promoted ring expansion reaction of 2-benzylidene-indane-1,3-diones with diynoates initiated by phosphine α-addition. This method provides rapid and efficient access to functionalized naphthoquinones fused to a five-membered ring bearing phosphorus ylides—which are usually difficult to prepare. This reaction represents a new strategy for the construction of this tricyclic framework through one-carbon-atom ring expansion along with acyl 1,2-migration. Moreover, further ring contraction through [1,5] sigmatropic rearrangement to the spiro indan-1,3-diones by *m*CPBA oxidation was revealed and inferred through oxidation, followed by protonation. Electrochemical studies show that the naphthoquinones and lactones with phosphorus could be applied toward redox colorimetric materials.

## Data Availability

The data underlying this study are available in the published article and its Supporting Information.

## Supporting Information Available

Detailed experimental procedures, spectral data and ^1^H and ^13^C spectra for compounds (PDF)
