# Supplementary material for: Phosphine-Promoted Synthesis of Naphthoquinones Fused with Cyclopentadienyl Moiety Via Ring Expansion: Synthesis, Reactivity, and Ring Contraction Via [1,5] Sigmatropic Rearrangement
Source: Org Lett. 2024 Oct 7;26(41):8730–5. doi: 10.1021/acs.orglett.4c03052 (PMC11494658; doi:10.1021/acs.orglett.4c03052)
Supplement: Supplementary file 1 — ol4c03052_si_001.pdf [file ol4c03052_si_001.pdf]

# Supporting Information

## Phosphine-promoted synthesis of naphthoquinones fused with cyclopentadienyl moiety via ring expansion: synthesis, reactivity, and ring contraction via [1,5] sigmatropic rearrangement

Wei-Qing Wang,[1] Sureshbabu Nallapati,[1] Chun-Yu Chen,[1] Tomoya Yaoita,[2] Shuri Yamaoka,[2] Michihisa Murata,[2,*] Shih-Ching Chuang[1,*]

[1]Department of Applied Chemistry, National Chiao Tung University, Hsinchu, Taiwan

[2]Department of Applied Chemistry, Osaka Institute of Technology, Osaka, Japan

Email: jscchuang@nycu.edu.tw; michihisa.murata@oit.ac.jp

## Contents

General Information ........ 2
General procedure for synthesis of 3 ........ 2
Procedure for the 1.0 mmol reaction scale of 2-benzylidene-1,3-indanedione (1a), diynoate 2a and $PCy_3$ ........ 3
General procedure for the synthesis of compound 4a-c. ........ 3
Spectroscopic and physical data of all new compounds ........ 4
Spectra for all new compounds ........ 17
Crystal growth method and XRD data for compounds 3h and 4a ........ 36
Table S1. Crystal data and experimental details for compound 3h (CCDC 2377008) ........ 37
Figure S39. Solid state structure of compound 3h ........ 38
Table S2. Crystal data and structure refinement for compound 4a (CCDC 2377009) ........ 39
Figure S40. Solid state structure of compound 4a ........ 40
Atomic coordinates of optimized structures ........ 41

## General Information

All reactions were performed under open air atmosphere using oven dried glassware. Reactions were monitored using TLC plate under short/long range ultraviolet light. Flash chromatography was performed on silica gel (230-400 mesh) using hexanes and ethyl acetate. $^{1}$H-NMR spectra were obtained on Agilent 400 MHz unless otherwise noted, and chemical shifts were reported in parts per million (ppm, δ) with *d*-$CHCl_3$ as a standard reference and internal standard as TMS. Proton coupling patterns are described as singlet (s), doublet (d), triplet (t), triplet of doublet (td), doublet of doublet (dd), multiplet (m); coupling constants (*J*) are quoted in Hz. $^{13}$C-NMR data were acquired at 100 MHz unless otherwise specified. High-resolution mass spectrometry (HR-MS)-electron spray ionization (ESI) mode was used to measure the compounds mass values. Melting points were determined using melting point apparatus; IR spectra were recorded on a Bruker spectrometer. Solvents were distilled prior to use. All chemicals were used as purchased unless otherwise mentioned. 2-Arylidene-1,3-indanediones **1** and diynoates **2** were prepared according to reported procedures.[1,2] We perform the computational study using Gaussian 16, Revision B.01 software. Geometry optimizations and frequency calculations are carried out with B3LYP/6-31G(d,p) for all atoms.[3]

## General procedure for synthesis of 3

We carried out reaction in aprotic etherate solvent tetrahydrofuran (THF, 10 mL) by three-component assembly of 2-arylidene-1,3-indanedione **1** (0.20 mmol), diynoates **2** (0.30 mmol) and tricyclohexyl phosphine (0.24 mmol) stirred at 0 °C for 24 h. Using this reaction condition, we proceeded to explore the substrate scope of the reaction using various substituted diynoates and various 2-arylideneindane-

---

1 Lee, C.J; Shu, CN, T Sai, CC; Wu, Z.Z., Lin, W.W. *Chem.Commun.* **2014**,50,5304.

2 J.-C. Deng, C.-W. Kuo, S.-C. Chuang, *Chem. Commun.* **2014**, *50*, 10580-10583.

3 Gaussian 16, Revision B.01, M. J. Frisch, G. W. Trucks, H. B. Schlegel, G. E. Scuseria, M. A. Robb, J. R. Cheeseman, G. Scalmani, V. Barone, G. A. Petersson, H. Nakatsuji, X. Li, M. Caricato, A. V. Marenich, J. Bloino, B. G. Janesko, R. Gomperts, B. Mennucci, H. P. Hratchian, J. V. Ortiz, A. F. Izmaylov, J. L. Sonnenberg, D. Williams-Young, F. Ding, F. Lipparini, F. Egidi, J. Goings, B. Peng, A. Petrone, T. Henderson, D. Ranasinghe, V. G. Zakrzewski, J. Gao, N. Rega, G. Zheng, W. Liang, M. Hada, M. Ehara, K. Toyota, R. Fukuda, J. Hasegawa, M. Ishida, T. Nakajima, Y. Honda, O. Kitao, H. Nakai, T. Vreven, K. Throssell, J. A. Montgomery, Jr., J. E. Peralta, F. Ogliaro, M. J. Bearpark, J. J. Heyd, E. N. Brothers, K. N. Kudin, V. N. Staroverov, T. A. Keith, R. Kobayashi, J. Normand, K. Raghavachari, A. P. Rendell, J. C. Burant, S. S. Iyengar, J. Tomasi, M. Cossi, J. M. Millam, M. Klene, C. Adamo, R. Cammi, J. W. Ochterski, R. L. Martin, K. Morokuma, O. Farkas, J. B. Foresman, and D. J. Fox, Gaussian, Inc., Wallingford CT, 2016.

1,3-diones employed in the reaction to give the functionalized 4,9-dioxo-3-phenyl-3a,9-dihydro-4*H*-cyclopenta[*b*]naphthalen-1-yl)-2-(tricyclohexyl-l5-phosphanylidenes (**3a-p**) in moderate to good yields. The reaction mixtures were subjected to flash column chromatography using hexanes and ethyl acetate as eluents. The collected solutions were evacuated to nearly dryness and later precipitated with hexanes to give pure solids.

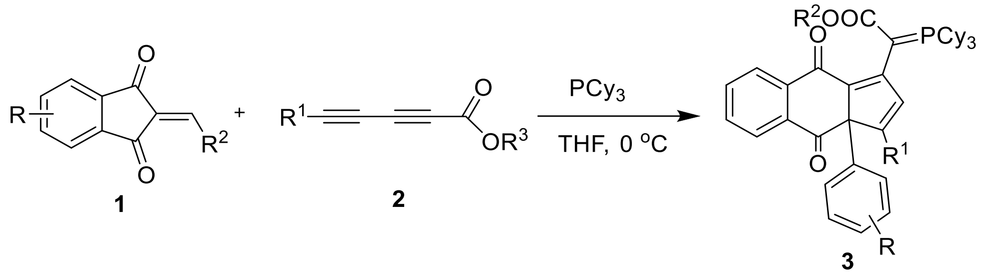


**Procedure for the 1.0 mmol reaction scale of 2-benzylidene-1,3-indanedione (1a), diynoate 2a and $PCy_3$**

To a solution containing 2-benzylidene-1,3-indanedione (1.0 mmol, 234 mg) and diynoate **2a** (1.5 mmol, 276 mg) at 0 °C was added $PCy_3$ (1.2 mmol, 336 mg) in 50 mL anhydrous THF. The resulting mixture was stirred for 24 hours. The reaction mixtures were subjected to flash column chromatography using hexanes and ethyl acetate as eluents after solvents were removed under reduced pressure. The collected solutions were evacuated to nearly dryness and later precipitated with hexanes to give pure solid **3a** in 58% yield (405 mg).

**General procedure for the synthesis of compound 4a-c.**

To a 20 mL anhydrous DCM solution containing Compound **3** (0.014 mmol) was added 10 mL of DCM containing *m*CPBA (0.034 mmol) through a syringe slowly in 30 min at room temperature. The mixture was then stirred for another 30 min. Upon completion of the reaction, the solution was quenched with saturated sodium bicarbonate for 30 min and extracted with DCM. The extract was dried with sodium sulfate. After evaporation of DCM, the resulting solids were re-dissolved in chloroform (2 mL), precipitated with *n*-hexane (6 mL) and centrifuged for three times to give pure solids **4**.

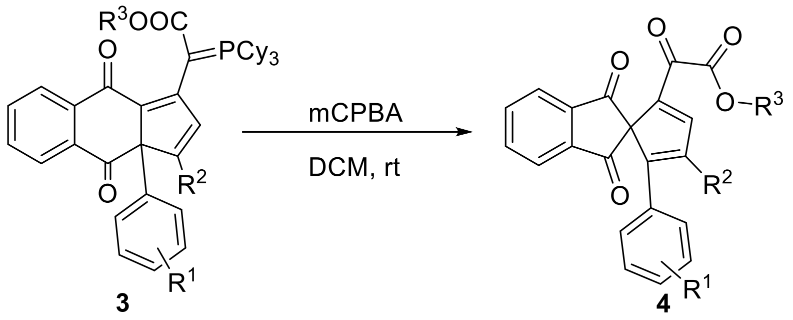


**Spectroscopic and physical data of all new compounds**

**Methyl 2-(4,9-dioxo-3,3a-diphenyl-3a,9-dihydro-4*H*-cyclopenta[*b*]naphthalen-1-yl)-2-(tricyclohexyl- $\lambda^5$-phosphaneylidene) acetate (3a)**

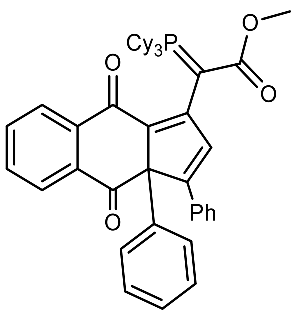


Yield 62% (86.7 mg), red solid, m.p. 147−150 ºC; $R_f$ = 0.28 (hexanes/EA = 7:3); $^1$H NMR ($CDCl_3$, 400 MHz) δ 7.98 (d, $J$ = 6.8 Hz, 1H), 7.84 (d, $J$ = 7.6 Hz, 1H), 7.55–7.48 (m, 2H), 7.38–7.35 (m, 3H), 7.24 (d, $J$ = 6.0 Hz, 2H), 7.18 (d, $J$ = 6.8 Hz, 2H), 7.07–7.01 (m, 3H), 6.69 (s, 1H), 3.64 (s, 3H), 2.80–2.66 (m, 3H), 2.34–2.20 (m, 3H), 1.92–1.86 (m, 3H), 1.78–1.70 (m, 9H), 1.61–1.45 (m, 6H), 1.35–1.23 (m, 4H), 1.20–1.14 (m, 5H) ppm; $^{13}$C NMR ($CDCl_3$, 100 MHz) δ 196.5, 176.6, 171.4 (d, $^2J_{PC}$ = 16.3 Hz), 156.1, 155.9 (d, $^2J_{PC}$ = 7.6 Hz), 138.3, 137.9, 136.5, 135.48, 135.44, 135.39, 133.1, 131.5, 128.5 (d, $^3J_{PC}$ = 12.1 Hz), 128.0, 127.87, 127.80, 127.1, 127.06, 127.01, 126.1, 77.9, 50.1, 44.8 (d, $^1J_{PC}$ = 107.0 Hz), 32.9 (d, $^1J_{PC}$ = 47.8 Hz), 27.9 (d, $^2J_{PC}$ = 3.3 Hz), 27.6, 27.5 (d, $^3J_{PC}$ = 9.2 Hz), 27.4, 26.0 ppm; FT-IR (KBr) $\tilde{\nu}$ (cm$^{-1}$) 2931, 1693, 1637, 1128; HRMS (ESI) m/z: $[M + H]^+$ Calcd for $C_{46}H_{52}O_4P$ 699.3603; Found 699.3603.

**Methyl 2-(4,9-dioxo-3-phenyl-3a-(p-tolyl)-3a,9-dihydro-4*H*-cyclopenta[b]naphthalen-1-yl)-2 -(tricyclohexyl-$\lambda^5$-phosphanylidene) acetate (3b)**

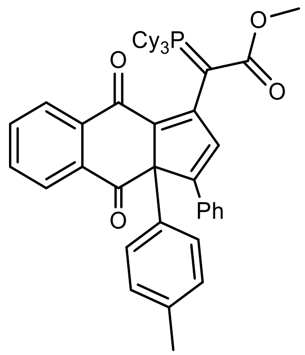

Yield 64% (91.3 mg), red solid, m.p. 212–215 ºC; $R_f$ = 0.40 (hexanes/EA = 7:3); $^1$H NMR ($CDCl_3$, 400 MHz) δ 7.98 (d, $J$ = 6.8, 1H), 7.83 (d, $J$ = 6.8, 1H), 7.54–7.47 (m, 2H), 7.39–7.38 (m, 2H), 7.25–7.23 (m, 3H), 7.10–7.0 (d, $J$ = 7.6, 2H), 6.90–6.84 (d, $J$ = 7.6, 2H), 6.68 (s, 1H), 3.62 (s, 3H), 2.72–2.70 (m, 3H), 2.13 (s, 3H), 2.08–2.07 (m, 3H), 1.90–1.88 (m, 3H), 1.82–1.70 (m, 7H), 1.58–1.47 (m, 7H), 1.32–1.04 (m, 10H) ppm; $^{13}$C NMR ($CDCl_3$, 100 MHz) δ 197.3, 176.5, 171.5 (d, $^2J_{PC}$ = 15.6 Hz), 157.4 (d, $^2J_{PC}$ = 8.0 Hz), 155.4, 144.4, 138.4, 138.3, 137.9, 136.5, 134.2, (d, $^3J_{PC}$ = 9.5 Hz), 133.6, 133.6, 132.2, 131.8, 131.6, 128.9, 128.5, 127.93, 127.91, 127.3, 126.0, 118.5, 110.8, 77.7, 50.2, 44.5 (d, $^1J_{PC}$ = 106.9 Hz), 32.9 (d, $^1J_{P\text{-}C}$ = 47.8 Hz), 27.9 (d, $^2J_{PC}$ = 3.0 Hz), 27.6, 27.5, 27.4, 26.0, 20.9 ppm; FT-IR (KBr) $\tilde{\nu}$ (cm$^{-1}$) 2930, 1694, 1637, 1129; HRMS (ESI) m/z: [M + H]$^+$ Calcd for $C_{47}H_{54}O_4P$ 713.3760; Found 713.3754.

**Methyl 2-(3a-(4-fluorophenyl)-4,9-dioxo-3-phenyl-3a,9-dihydro-4*H*-cyclopenta[*b*]naphthalen-1-yl)-2-(tricyclohexyl- *λ*$^5$-phosphaneylidene) acetate (3c)**

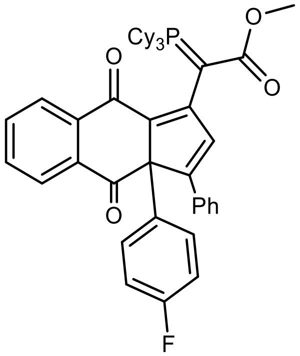

Yield 60% (86.0 mg), red solid, m.p. 172−173 ºC; $R_f$ = 0.38 (hexanes/EA = 7 : 3); $^1$H NMR ($CDCl_3$, 400 MHz) δ 7.99 (d, $J$ = 7.6 Hz, 1H), 7.81 (d, $J$ = 7.2 Hz, 1H), 7.56–7.48 (m, 2H), 7.40 (d, $J$ = 6.0 Hz, 2H), 7.31–7.25 (m, 1H), 7.22–7.18 (m, 2H), 7.14 (d, $J$ = 8.4 Hz, 2H), 7.00 (d, $J$ = 8.4 Hz, 2H), 6.69 (s, 1H), 3.62 (s, 3H), 4.21–4.07 (m, 2H), 2.80–2.60 (m, 3H), 2.28–2.20 (m, 3H), 1.90–1.77 (m, 3H), 1.74–1.54

(m, 9H), 1.50–1.45 (m, 4H), 1.34–1.16 (m, 6H), 1.14 (t, $J$ = 7.0 Hz, 3H) ppm; $^{13}$C NMR ($CDCl_3$, 100 MHz) δ 197.2, 177.1, 171.5 (d, $^2J_{PC}$ = 16.1 Hz), 162.9 (d, $^1J_{FC}$ = 245.1 Hz), 156.2 (d, $^2J_{PC}$ = 15.0 Hz), 138.3 (d, $^2J_{FC}$ = 24.0 Hz), 136.6, 135.3, 135.0 (d, $^3J_{PC}$ = 10.0 Hz), 133.7 (d, $^4J_{FC}$ = 3.2 Hz), 133.3, 132.1 (d, $^3J_{FC}$ = 6.3 Hz), 131.6, 128.8 (d, $^3J_{FC}$ = 8.0 Hz), 128.6, 128.3, 127.8, 127.9, 127.1, 126.1, 115.5 (d, $^2J_{FC}$ = 22.7 Hz), 77.2, 50.2, 45.2 (d, $^1J_{PC}$ = 106.6 Hz), 32.9 (d, $^1J_{PC}$ = 47.0 Hz), 31.5, 27.9 (d, $^2J_{PC}$ = 3.4 Hz), 27.6, 27.5, 27.4, 26.0, 22.6 ppm; FT-IR (KBr) $\tilde{\nu}$ (cm$^{-1}$) 2929, 1694, 1635, 1120; HRMS (ESI) m/z: $[M + H]^+$ Calcd for $C_{46}H_{51}FO_4P$ 717.3509; Found 717.3491.

**Methyl 2-(3a-(4-chlorophenyl)-4,9-dioxo-3-phenyl-3a,9-dihydro-4*H*-cyclopenta[*b*]naphthalen-1-yl) 2 -(tricyclohexyl-$\lambda^5$-phosphanylidene) acetate (3d)**

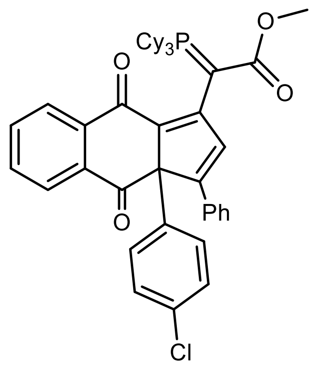


Yield 66% (96.8 mg), red solid, m.p. 158–160 ºC; $R_f$ = 0.36 (hexanes/EA = 7:3); $^1$H NMR ($CDCl_3$, 400 MHz) δ 7.98 (d, $J$ = 7.2 Hz, 1H), 7.90 (d, $J$ = 8.4 Hz, 2H), 7.84 (d, $J$ = 6.8 Hz, 1H), 7.58–7.51 (m, 2H), 7.41–7.39 (m, 2H), 7.35–7.33 (d, $J$ = 8.0 Hz , 2H), 7.26–7.25 (m , 3H), 6.70 (s, 1H), 3.64 (s, 1H), 2.74–2.65 (m, 3H), 2.06 (m, 3H), 1.91–1.86 (m, 3H), 1.77–1.70 (m, 8H), 1.60–1.46 (m, 8H), 1.35–1.13 (m, 10H) ppm; $^{13}$C NMR ($CDCl_3$, 100 MHz) δ 196.5, 176.5, 171.5 (d, $^2J_{PC}$ = 15.5 Hz), 157.3 (d, $^2J_{PC}$ = 7.9 Hz), 155.2, 146.8, 146.3, 138.3, 137.8, 134.5, 134.1, (d, $^3J_{PC}$ = 9.8 Hz), 133.7, 131.9, 128.6, 128.4, 128.1, 128.0, 127.4, 126.1, 123.7, 50.2, 46.1 (d, $^1J_{PC}$ = 105.8 Hz), 32.9 (d, $^1J_{PC}$ = 47.4 Hz), 27.9 (d, $^2J_{PC}$ = 3.1 Hz), 27.5, 27.4, 27.3 26.9, 26.01 ppm; FT-IR (KBr) $\tilde{\nu}$ (cm$^{-1}$) 2931, 1697, 1639, 1128; HRMS (ESI) m/z: $[M + H]^+$ Calcd for $C_{46}H_{51}ClO_4P$ 733. 3213; Found 733.3227.

**Methyl 2-(3a-(4-bromophenyl)-4,9-dioxo-3-phenyl-3a,9-dihydro-4*H*-cyclopenta[b]naphthalen-1-yl)-2-(tricyclohexyl- $\lambda^5$-phosphaneylidene) acetate (3e)**

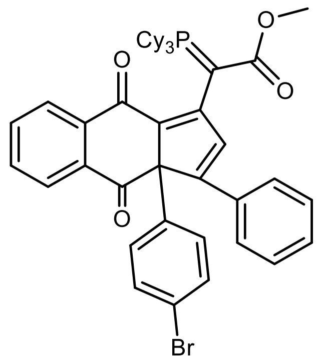

Yield 78% (121.3 mg), red solid, m.p. 148−150 ºC; $R_f$ = 0.35 (hexanes/EA = 7:3); $^{1}$H NMR ($CDCl_3$, 400 MHz) δ 7.99 (d, $J$ = 7.2 Hz, 1H), 7.81 (d, $J$ = 7.2 Hz, 1H), 7.56–7.48 (m, 3H), 7.40 (d, $J$ = 6.4 Hz, 1H), 7.28–7.27 (m, 1H), 7.25–7.24 (m, 2H), 7.17 (d, $J$ = 8.4 Hz, 2H), 7.06 (d, $J$ = 8.4 Hz, 2H), 6.69 (s, 1H), 3.62 (s, 3H), 2.74–2.65 (m, 3H), 2.12–1.98 (m, 3H), 1.90–1.83 (m, 3H), 1.77–1.69 (m, 8H), 1.49–1.43 (m, 6H), 1.35–1.13 (m, 10H) ppm; $^{13}$C NMR ($CDCl_3$, 100 MHz) δ 196.9, 177.0, 171.5 (d, $^{2}J_{PC}$ = 16.1 Hz), 156.4 (d, $^{2}J_{PC}$ = 8.3 Hz), 155.7, 138.3, 138.1, 137.3, 136.7, 135.0, 134.8 (d, $^{3}J_{PC}$ = 9.5 Hz), 133.4, 131.7, 131.5, 128.9, 128.6, 128.0, 127.9, 127.1, 126.1, 120.0, 50.2, 45.3 (d, $^{1}J_{PC}$ = 106.6 Hz), 32.9 (d, $^{1}J_{PC}$ = 47.5 Hz), 27.95 (d, $^{2}J_{PC}$ = 3.2 Hz), 27.5, 27.4 (d, $^{3}J_{PC}$ = 3.3 Hz), 27.3, 26.0 ppm; FT-IR (KBr) $\tilde{\nu}$ ($cm^{-1}$) 2930, 1694, 1638, 1559, 1128; HRMS (ESI) m/z: $[M + H]^+$ Calcd for $C_{46}H_{51}BrO_4P$ 777.2708; Found 777.2702.

**Methyl 2-(3a-(4-cyanophenyl)-4,9-dioxo-3-phenyl-3a,9-dihydro-4*H*-cyclopenta[*b*]naphthalen-1-yl)-2-(tricyclohexyl- $\lambda^5$-phosphaneylidene) acetate (3f)**

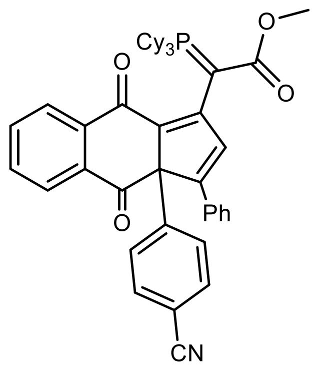

Yield 67% (97.0 mg), red solid, m.p. 152−154 ºC; $R_f$ = 0.26 (hexanes/EA = 7:3); $^{1}$H NMR ($CDCl_3$, 400 MHz) δ 7.98 (dd, $J$ =1.2, 7.4 Hz, 1H), 7.82 (dd, $J$ = 1.2, 6.8 Hz, 1H), 7.57–7.49 (m, 2H), 7.38–7.36 (m, 2H), 7.35–7.29 (m, 2H), 7.28–7.27 (m, 2H), 7.25–7.21 (m, 3H), 6.73 (s, 1H), 3.62 (s, 3H), 4.21–4.07 (m, 2H), 2.80–2.60 (m, 3H), 2.28–2.20 (m, 3H), 1.90–1.77 (m, 3H), 1.74–1.54 (m, 6H), 1.50–1.45 (m, 6H),

1.34–1.16 (m, 7H), 1.14 (t, $J$ = 13.3 Hz, 3H) ppm; $^{13}$C NMR ($CDCl_3$, 100 MHz) δ 196.5, 176.6, 171.4 (d, $^{2}J_{PC}$ = 15.5 Hz), 157.2 (d, $^{2}J_{PC}$ = 7.9 Hz), 155.2, 144.1, 138.3, 137.8, 137.1, 134.5, 134.1, (d, $^{3}J_{PC}$ = 9.5 Hz), 133.6, 132.2, 131.8, 128.5, 128.2, 128.0, 127.8, 127.3, 126.1, 118.5, 110.9, 77.5, 50.2, 46.0 (d, $^{1}J_{PC}$ = 105.8 Hz), 32.9 (d, $^{1}J_{PC}$ = 47.4 Hz), 27.9 (d, $^{2}J_{PC}$ = 3.1 Hz), 27.5, 27.4 (d, $^{3}J_{PC}$ = 3.7 Hz), 27.3, 26.0 ppm; FT-IR (KBr) $\tilde{\nu}$ (cm$^{-1}$) 2931, 1700, 1639, 1129; HRMS (ESI) m/z: $[M + H]^+$ Calcd for $C_{47}H_{50}NO_4P$ 724.3556; Found 724.3529.

**Methyl 2-(3a-(4-nitrophenyl)-4,9-dioxo-3-phenyl-3a,9-dihydro-4*H*-cyclopenta[*b*]naphthalen-1-yl)-2-(tricyclohexyl-*λ*$^5$-phosphanylidene) acetate (3g)**

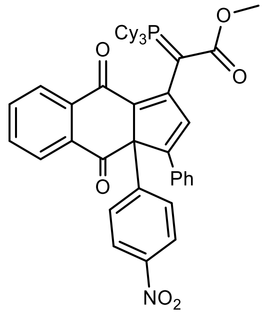


Yield 68% (101.2 mg), red solid, m.p. 197–200 ºC; $R_f$ = 0.36 (hexanes/EA = 7:3); $^{1}$H NMR ($CDCl_3$, 400 MHz) δ 7.99 (dd, $J$ = 1.6, 6.8 Hz, 1H), 7.97–7.88 (m, 2H), 7.85 (dd, $J$ = 1.2, 7.2 Hz, 1H), 7.57–7.53 (m, 2H), 7.36–7.33 (m, 2H), 7.29–7.26 (m, 2H), 7.25–7.24 (m, 3H), 6.77 (s, 1H), 3.65 (s, 3H), 2.74–2.66 (m, 3H), 2.12–2.06 (m, 3H), 1.92–1.89 (m, 3H), 1.70–1.71 (m, 9H), 1.61–1.43 (m, 6H), 1.36–1.14 (m, 9H) ppm; $^{13}$C NMR ($CDCl_3$, 100 MHz) δ 196.5, 176.5, 171.4 (d, $^{2}J_{PC}$ = 15.6 Hz), 157.3 (d, $^{2}J_{PC}$ = 8.0 Hz), 155.1, 146.8, 146.3, 138.3, 137.8, 137.3, 134.5, 134.1 (d, $^{3}J_{PC}$ = 9.5 Hz), 133.7, 131.9, 128.6, 128.3, 128.1, 128.0, 127.3, 126.1, 123.7, 50.3, 45.6 (d, $^{1}J_{PC}$ = 105.9 Hz), 32.9 (d, $^{1}J_{PC}$ = 47.9 Hz), 27.9 (d, $^{2}J_{PC}$ = 3.0 Hz), 27.5, 27.4 (d, $^{3}J_{PC}$ = 12.0 Hz), 27.3, 26.0 ppm; FT-IR (KBr) $\tilde{\nu}$ (cm$^{-1}$) 2932, 1696,1639, 1518, 1128; HRMS (ESI) m/z: $[M + H]^+$ Calcd for $C_{46}H_{51}NO_6P$ 744.3454; Found 744.3443.

**Methyl 2-(3a-(3-methoxyphenyl)-4,9-dioxo-3-phenyl-3a,9-dihydro-4*H*-cyclopenta[*b*]naphthalen-1-yl)-2-(tricyclohexyl-*λ*$^5$-phosphanylidene) acetate (3h)**

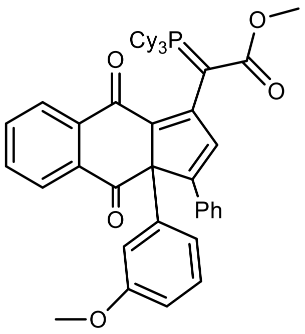

Yield 61% (88.9 mg), red solid, m.p. 194–196 ºC; $R_f$ = 0.28 (hexanes/EA = 7:3); $^1$H NMR ($CDCl_3$, 400 MHz) δ 7.99 (d, $J$ = 7.6, 1H), 7.82 (d, $J$ = 7.6, 1H), 7.55–7.47 (m, 2H), 7.39–7.38 (m, 2H), 7.25–7.23 (m, 1H), 7.24–7.21 (m, 2H), 6.99–6.94 (t, 1H), 6.78–6.75 (m, 2H), 6.66 (s, 1H), 6.58–6.56 (dd, $J$ = 1.6 , 6.4 Hz 1H), 3.64 (s, 3H), 3.60 (s, 3H), 2.75–2.66 (m, 3H), 2.06 (m, 3H), 1.90–1.88 (m, 3H), 1.77–1.70 (m, 7H), 1.60–1.44 (m, 7H), 1.36–1.10 (m, 10H) ppm; $^{13}$C NMR ($CDCl_3$, 100 MHz) δ 197.2, 177.2, 171.7 (d, $^2J_{PC}$ = 16.3 Hz), 159.5, 156.1 (d, $^2J_{PC}$ = 12.6 Hz), 139.3, 138.3,136.6, 135.6, 135.1, 135.0, 133.1, 131.5, 129.2, 128.6, 127.8, 127.1, 126.0, 119.8, 113.0,112.5, 77.9, 55.0, 50.1, 45.0 (d, $^1J_{PC}$ = 107.4 Hz), 32.9 (d, $^1J_{PC}$ = 47.5 Hz), 27.9 (d, $^2J_{PC}$ = 3.0 Hz), 27.5, 27.4 (d, $^3J_{PC}$ = 2.7 Hz), 27.3, 26.0 ppm; FT-IR (KBr) $\tilde{\nu}$ (cm$^{-1}$) 2931, 1694, 1637, 1127,; HRMS (ESI) m/z: [M + H]$^+$ Calcd for $C_{47}H_{54}O_5P$ 729.3708; Found 729.3689.

**Methyl 2-(3a-(naphthalen-2-yl)-4,9-dioxo-3-phenyl-3a,9-dihydro-4*H*-cyclopenta[b]naphthalen-1-yl)-2-(tricyclohexyl- $\lambda^5$-phosphaneylidene) acetate (3i)**

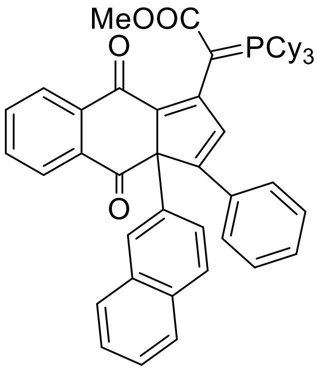

Yield 66% (98.9 mg), red solid, m.p. 170−172 ºC; $R_f$ = 0.34 (hexanes/EA = 7:3); $^1$H NMR ($CDCl_3$, 400 MHz) δ 7.99–7.87 (m, 2H), 7.67–7.57 (m, 3H), 7.57–7.47 (m, 3H), 7.45–7.32 (m, 3H), 7.32–7.25 (m, 2H), 7.25–7.16 (m, 2H), 7.11 (d, $J$ = 1.4 Hz, 1H), 6.66 (s, 1H), 3.65 (s, 3H), 2.77–2.67 (m, 3H), 2.05 (bs, 3H), 1.92–1.88 (m, 3H), 1.78–1.71 (m, 8H), 1.61–1.44 (m, 6H), 1.37–1.14 (m, 10H) ppm; $^{13}$C NMR

($CDCl_3$, 100 MHz) δ 195.3, 174.3, 171.6 (d, $^2J_{PC}$ = 16.0 Hz), 157.8 (d, $^2J_{PC}$ = 8.0 Hz), 156.3, 138.0, 137.9, 137.5, 137.0, 136.3, 136.0, 135.2, 133.9 (d, $^3J_{PC}$ = 9.0 Hz), 129.9, 128.7, 128.6, 128.0, 127.9, 127.4, 127.1, 77.4, 50.3, 47.0 (d, $^1J_{PC}$ = 105.0 Hz), 32.9 (d, $^1J_{PC}$ = 48.0 Hz), 27.9 (d, $^2J_{PC}$ = 3.0 Hz), 27.6, 27.5, 27.4, 26.0 ppm; FT-IR (KBr) $\tilde{\nu}$ ($cm^{-1}$) 2929, 1694, 1635, 1120; HRMS (ESI) m/z: $[M + H]^+$ Calcd for $C_{50}H_{54}O_4P$ 749.3760; Found 749.3770.

**Ethyl 2-(3a-(4-chlorophenyl)-4,9-dioxo-3-phenyl-3a,9-dihydro-4*H*-cyclopenta[*b*]naphthalen-1-yl)-2-(tricyclohexyl- $\lambda^5$-phosphaneylidene) acetate (3j)**

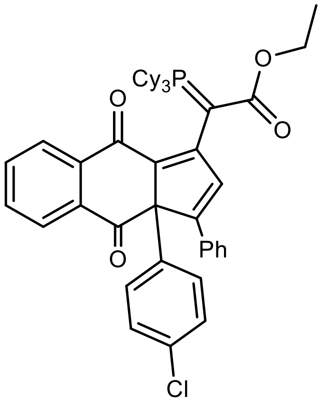


Yield 72% (107.6 mg), red solid, m.p. 194−196 ºC; $R_f$ = 0.40 (hexanes/EA = 7:3); $^1$H NMR ($CDCl_3$, 400 MHz) δ 7.99 (d, $J$ = 7.6 Hz, 1H), 7.81 (d, $J$ = 7.2 Hz, 1H), 7.56–7.48 (m, 2H), 7.40 (d, $J$ = 6.0 Hz, 2H), 7.31–7.25 (m, 1H), 7.22–7.18 (m, 2H), 7.14 (d, $J$ = 8.4 Hz, 2H), 7.00 (d, $J$ = 8.4 Hz, 2H), 6.69 (s, 1H), 3.62 (s, 3H), 4.21–4.07 (m, 2H), 2.80–2.60 (m, 3H), 2.28–2.20 (m, 3H), 1.90–1.77 (m, 3H), 1.74–1.54 (m, 8H), 1.50–1.45 (m, 5H), 1.34–1.16 (m, 8H), 1.14 (t, $J$ = 7.0 Hz, 3H) ppm; $^{13}$C NMR ($CDCl_3$, 100 MHz) δ 197.1, 177.0, 171.3 (d, $^2J_{PC}$ = 15.2 Hz), 156.5 (d, $^2J_{PC}$ = 8.0 Hz), 155.7, 138.4, 138.1, 136.9, 136.7, 135.1, 134.7 (d, $^3J_{PC}$ = 9.9 Hz), 133.3, 132.8, 131.6, 128.7, 128.6, 128.5, 128.0, 127.9, 127.1, 126.0, 77.4, 58.7, 45.7 (d, $^1J_{PC}$ = 106.3 Hz), 32.9 (d, $^1J_{PC}$ = 47.4 Hz), 27.9 (d, $^2J_{PC}$ = 3.0 Hz), 27.6, 27.5, 27.4, 26.0, 14.8 ppm; FT-IR (KBr) $\tilde{\nu}$ ($cm^{-1}$) 2929, 1694, 1635, 1120; HRMS (ESI) m/z: $[M + H]^+$ Calcd for $C_{47}H_{53}ClO_4P$ 747.3370; Found 747.3388.

**Ethyl 2-(3a-(4-bromophenyl)-4,9-dioxo-3-phenyl-3a,9-dihydro-4*H*-cyclopenta[b]naphthalen-1-yl)-2-(tricyclohexyl- $\lambda^5$-phosphaneylidene) acetate (3k)**

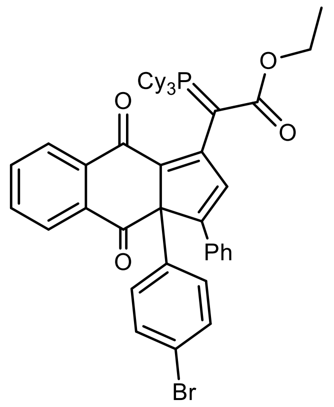

Yield 70% (110.9 mg), red solid, m.p. 195−198 ºC; $R_f$ = 0.40 (hexanes/EA = 7:3); $^1$H NMR ($CDCl_3$, 400 MHz) δ 7.99 (d, $J$ = 7.6 Hz, 1H), 7.81 (d, $J$ = 7.2 Hz, 1H), 7.56–7.48 (m, 2H), 7.40 (d, $J$ = 6.0 Hz, 2H), 7.31–7.25 (m, 1H), 7.22–7.18 (m, 2H), 7.14 (d, $J$ = 8.4 Hz, 2H), 7.00 (d, $J$ = 8.4 Hz, 2H), 6.69 (s, 1H), 4.21–4.07 (m, 2H), 2.80–2.60 (m, 3H), 2.28–2.20 (m, 3H), 1.90–1.77 (m, 3H), 1.74–1.54 (m, 9H), 1.50–1.45 (m, 6H), 1.34–1.16 (m, 9H), 1.14 (t, $J$ = 7.1 Hz, 3H) ppm; $^{13}$C NMR ($CDCl_3$, 100 MHz) δ 197.1, 177.0, 171.3 (d, $^2J_{PC}$ = 15.0 Hz), 156.5 (d, $^2J_{PC}$ = 8.0 Hz), 155.7, 138.4, 138.1, 136.9, 136.7, 135.1, 134.7 (d, $^3J_{PC}$ = 9.9 Hz), 133.3, 132.8, 131.6, 128.7, 128.6, 128.5, 128.0, 127.9, 127.1, 126.0, 77.5, 58.7, 45.2 (d, $^1J_{PC}$ = 107.0 Hz), 32.7 (d, $^1J_{PC}$ = 47.0 Hz), 28.0 (d, $^2J_{PC}$ = 3.1 Hz), 27.6, 27.4 (d, $^3J_{PC}$ = 7.0 Hz), 27.3, 26.1, 14.9 ppm; FT-IR (KBr) $\tilde{\nu}$ (cm$^{-1}$) 2929, 1694, 1635, 1120; HRMS (ESI) m/z: [M + H]$^+$ Calcd for $C_{47}H_{53}BrO_4P$ 791.2865; Found 791.2872.

**Ethyl 2-(3a-(4-cyanophenyl)-4,9-dioxo-3-phenyl-3a,9-dihydro-4H-cyclopenta[b]naphthalen-1-yl) 2-(tricyclohexyl-$\lambda^5$-phosphanylidene) acetate (3l)**

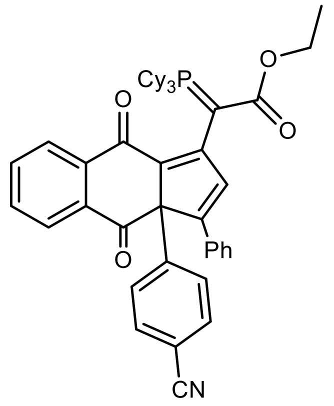

Yield 64% (94.5 mg), red solid, m.p. 194–197 ºC; $R_f$ = 0.34 (hexanes/EA = 7:3); $^1$H NMR ($CDCl_3$, 400 MHz) δ 7.98 (dd, $J$ = 1.2, 6.4 Hz, 1H), 7.83 (dd, $J$ = 1.2, 6.4 Hz, 1H), 7.58–7.50 (m, 2H), 7.39–7.32 (m,

2H), 7.31–7.29 (m, 4H), 7.27–7.26 (m, 1H), 7.26–7.24 (m, 2H), 6.74 (s, 1H), 4.20–4.08 (m, 2H), 2.74–2.65 (m, 3H), 2.06 (m, 3H), 1.90–1.87 (m, 4H), 1.76–1.69 (m, 8H), 1.60–1.43 (m, 6H), 1.35–1.16 (m, 9H), 1.13–1.10 (t, 3H) ppm; $^{13}$C NMR ($CDCl_3$, 100 MHz) δ 196.6, 176.5, 171.2 (d, $^{2}J_{PC}$ = 15.5 Hz), 157.3 (d, $^{2}J_{PC}$ = 7.9 Hz), 155.1, 144.1, 138.4, 137.8, 137.3, 134.5, 133.9 (d, $^{3}J_{PC}$ = 9.5 Hz), 133.8, 133.6, 132.1, 131.7, 128.5, 128.2, 128.0, 127.9, 127.2, 126.0, 118.5, 110.9, 77.5, 58.7, 46.4 (d, $^{1}J_{PC}$ = 105.5 Hz), 32.9 (d, $^{1}J_{PC}$ = 47.4 Hz), 27.9 (d, $^{2}J_{PC}$ = 3.1 Hz), 27.4, 27.38, 27.31, 26.0, 14.8 ppm; FT-IR (KBr) $\tilde{\nu}$ (cm$^{-1}$) 3061, 2931, 1696,1636, 1561, 1129; HRMS (ESI) m/z: $[M + H]^+$ Calcd for $C_{48}H_{53}NO_4P$ 738.3712; Found 738.3714.

**Methyl 2-(3-(4-(tert-butyl)phenyl)-4,9-dioxo-3a-phenyl-3a,9-dihydro-4H-cyclopenta[b]naphthalen-1-yl)-2-(tricyclohexyl- *λ*$^5$-phosphaneylidene) acetate (3m)**

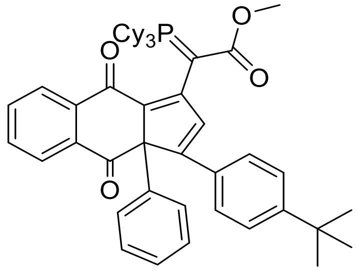


Yield 62% (93.6 mg), red solid, m.p. 172−173 °C; $R_f$ = 0.38 (hexanes/EA = 7:3); $^{1}$H NMR ($CDCl_3$, 400 MHz) δ 7.98 (d, *J* = 6.8 Hz, 1H), 7.85 (d, *J* = 8.4 Hz, 1H), 7.55–7.48 (m, 2H), 7.33 (d, *J* = 8.4 Hz, 2H), 7.26–7.24 (m, 2H), 7.20 (d, *J* = 7.2 Hz, 2H), 7.10–7.01 (m, 3H), 6.72 (s, 1H), 3.62 (s, 3H), 2.76–2.67 (m, 3H), 2.08 (bs, 3H), 1.92–1.88 (m, 3H), 1.82–1.70 (m, 10H), 1.58–1.43 (m, 8H), 1.28 (s, 9H), 1.20–1.14 (m, 6H) ppm; $^{13}$C NMR ($CDCl_3$, 100 MHz) δ 197.3, 177.0, 171.3 (d, $^{2}J_{PC}$ = 15.2 Hz), 156.5 (d, $^{3}J_{PC}$ = 8.0 Hz), 155.7, 138.4, 138.1, 136.9, 136.7, 135.1, 134.7 (d, $^{3}J_{PC}$ = 9.9 Hz), 133.3, 132.8, 131.6, 128.7, 128.6, 128.5, 128.0, 127.9, 127.1, 126.0, 77.4, 58.7, 45.7 (d, $^{1}J_{PC}$ = 106.3 Hz), 34.5, 32.9 (d, $^{1}J_{PC}$ = 47.4 Hz), 31.2, 27.9 (d, $^{2}J_{P\text{-}C}$ = 3.1 Hz), 27.6, 27.5, 27.4, 26.1, ppm; FT-IR (KBr) $\tilde{\nu}$ (cm$^{-1}$) 2929, 1694, 1635, 1120; HRMS (ESI) m/z: $[M + H]^+$ Calcd for $C_{50}H_{60}O_4P$ 755.4229; Found 755.4223.

**Methyl 2-(3a-(4-cyanophenyl)-4,9-dioxo-3-(p-tolyl)-3a,9-dihydro-4*H*-cyclopenta[*b*]naphthalen-1-yl) 2 -(tricyclohexyl-*λ*$^5$-phosphanylidene) acetate (3n)**

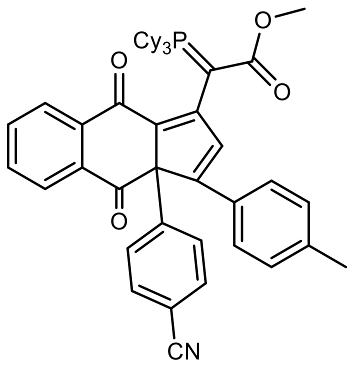

Yield 64% (94.5 mg), red solid, m.p. 196–199 ºC; $R_f$ = 0.32 (hexanes/EA = 7:3); $^1$H NMR ($CDCl_3$, 400 MHz) δ 7.98 (dd, $J$ =1.2 , 7.2 Hz, 1H), 7.83 (d, $J$ = 6.8 Hz, 1H), 7.57–7.50 (m, 2H), 7.34–7.26 (m, 6H), 7.07 (d, $J$ = 8.0 Hz, 1H), 6.87 (d, $J$ = 7.6 Hz, 1H), 6.72 (s, 1H), 3.63 (s, 1H), 2.73–2.64 (m, 3H), 2.3 (s, 3H), 2.05 (m, 3H), 1.90–1.88 (m, 3H), 1.76–1.69 (m, 8H), 1.60–1.42 (m, 8H), 1.35–1.09 (m, 10H) ppm; $^{13}$C NMR ($CDCl_3$, 100 MHz) δ 196.6, 176.5, 171.5 (d, $^2J_{PC}$ = 15.6 Hz), 157.4 (d, $^2J_{PC}$ = 7.6 Hz), 155.4, 144.4, 138.4, 138.3, 137.9, 136.5, 134.2, (d, $^3J_{PC}$ = 9.5 Hz), 133.6, 132.2, 131.8, 131.6, 128.9, 128.5, 127.9, 127.3, 126.0, 118.5, 110.8, 50.2, 45.9 (d, $^1J_{PC}$ = 105.5 Hz), 32.9 (d, $^1J_{PC}$ = 47.4 Hz), 27.92 (d, $^2J_{PC}$ = 3.3 Hz), 27.5, 27.4, 27.3 26.0, 21.2 ppm; FT-IR (KBr) $\tilde{\nu}$ (cm$^{-1}$) 3063, 2931, 1696, 1638, 1511, 1130; HRMS (ESI) m/z: $[M + H]^+$ Calcd for $C_{48}H_{53}NO_4P$ 738.3712; Found 738.3732.

**Methyl 2-(3-(4-(tert-butyl) phenyl)-3a-(4-cyanophenyl)-4,9-dioxo-3a,9-dihydro-4*H*-cyclopenta[b]naphthalen-1-yl)-2-(tricyclohexyl- $\lambda^5$-phosphaneylidene) acetate (3o)**

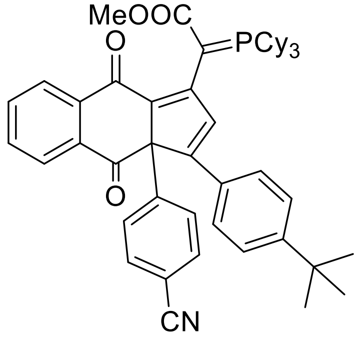

Yield 61% (95.2 mg), red solid, m.p. 98−100 ºC; $R_f$ = 0.3 (hexanes/EA = 7:3); $^1$H NMR ($CDCl_3$, 400 MHz) δ 7.99 (d, $J$ = 8.4 Hz, 1H), 7.85 (d, $J$ = 7.2 Hz, 1H), 7.58–7.53 (m, 2H), 7.36–7.25 (m, 8H), 6.76 (s, 1H), 3.63 (s, 3H), 2.74–2.65 (m, 3H), 2.06 (bs, 3H), 1.92–1.88 (m, 3H), 1.82–1.70 (m, 9H), 1.58–1.43 (m, 6H), 1.28 (s, 9H), 1.20–1.14 (m, 6H), 0.89 (t, $J$ = 6.9 Hz, 3H) ppm; $^{13}$C NMR ($CDCl_3$, 100 MHz) δ

196.6, 176.4, 171.3 (d, $^2J_{PC}$ = 15.5 Hz), 157.2 (d, $^2J_{PC}$ = 8.0 Hz), 155.2, 151.4, 144.4, 138.3, 137.9, 136.4, 134.4 (d, $^3J_{PC}$ = 9.0 Hz), 133.6, 132.2, 131.8, 131.5, 128.0, 127.9, 127.3, 126.0, 125.1, 118.6, 110.8, 77.2, 50.2, 45.8 (d, $J_{PC}$ = 105.9 Hz), 34.6, 32.9 (d, $^1J_{PC}$ = 47.0 Hz), 31.5, 31.1, 27.9 (d, $^2J_{PC}$ = 3.0 Hz), 27.5, 27.4, 27.3, 26.0, 22.6, 14.1 ppm; FT-IR (KBr) $\tilde{\nu}$ ($cm^{-1}$) 2929, 1694, 1635, 1120; HRMS (ESI) m/z: $[M + H]^+$ Calcd for $C_{51}H_{59}O_4NP$ 780.4182; Found 780.4178.

**Methyl 2-(6,7-dichloro-4,9-dioxo-3,3a-diphenyl-3a,9-dihydro-4H-cyclopenta[b]naphthalen-1-yl)-2-(tricyclohexyl- $\lambda^5$-phosphaneylidene) acetate (3p)**

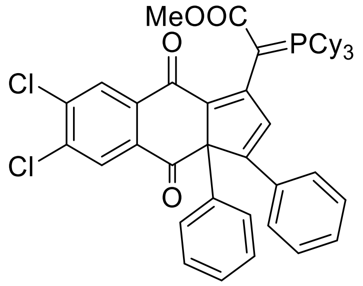


Yield 64% (98.3 mg), red solid, m.p. 158−160 ºC; $R_f$ = 0.36 (hexanes/EA = 7:3); $^1$H NMR ($CDCl_3$, 400 MHz) δ 8.05 (s, 1H), 7.87 (s, 1H), 7.33 (dt, $J$ = 5.9, 3.6 Hz, 2H), 7.27–7.21 (m, 3H), 7.21–7.09 (m, 3H), 7.09 (dd, $J$ = 8.0, 6.3 Hz, 2H), 6.66 (s, 1H), 3.65 (s, 3H), 2.72 (q, $J$ = 12.3, Hz, 3H), 2.08 (d, $J$ = 11.1 Hz, 3H), 1.95–1.86 (m, 3H), 1.84–1.69 (m, 10H), 1.63–1.41 (m, 6H), 1.39–1.09 (m, 8H) ppm; $^{13}$C NMR ($CDCl_3$, 100 MHz) δ 197.2, 177.2, 171.7 (d, $^2J_{PC}$ = 16.0 Hz), 156.2 (d, $^2J_{PC}$ = 8.0 Hz), 155.9, 138.4, 138.3, 136.8, 135.7, 135.6 (d, $^3J_{PC}$ = 9.0 Hz), 135.4, 133.3, 133.2, 132.3, 131.6, 128.5, 128.1, 127.9, 127.9, 127.8, 127.3, 127.1, 126.4, 126.1, 125.7, 125.1, 77.8, 50.2, 46.5 (d, $J_{PC}$ = 105.6 Hz), 32.9 (d, $^1J_{PC}$ = 48.0 Hz), 28.0 (d, $^2J_{PC}$ = 3.2 Hz), 27.6, 27.5, 27.4, 26.0 ppm; FT-IR (KBr) $\tilde{\nu}$ ($cm^{-1}$) 2929, 1694, 1635, 1120; HRMS (ESI) m/z: $[M + H]^+$ Calcd for $C_{46}H_{50}Cl_2O_4P$ 767.2824; Found 767.2821.

**Methyl 2-(1',3'-dioxo-2,3-diphenyl-1',3'-dihydrospiro[cyclopentane-1,2'-indene]-2,4-dien-5-yl)-2-oxoacetate (4a)**

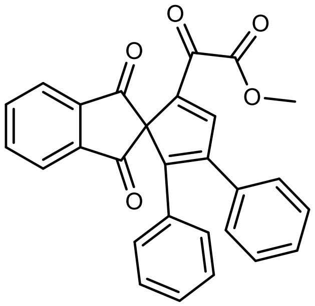

Yield 98% (6.1 mg), yellow solid, $R_f$ = 0.46 (DCM/Hexanes = 3:7); m.p. 142–144 °C; $^1$H NMR ($CDCl_3$, 400 MHz) δ 8.49 (s, 1H), 7.98 (dd, *J* = 5.7, 3.0 Hz, 2H), 7.85 (dd, *J* = 3.1, 2.7 Hz, 2H), 7.36 (dt, *J* = 8.6, 1.8 Hz, 2H), 7.32–7.24 (m, 4H), 7.24–7.19 (m, 2H), 7.07 (dt, *J* = 8.7, 1.7 Hz, 2H), 3.88 (s, 3H); $^{13}$C NMR (100 MHz, Chloroform-d) δ 195.0, 176.4, 161.2, 156.7, 150.2, 146.8, 143.5, 142.7, 135.8, 133.1, 133.0, 128.7, 128.6, 128.51, 128.50, 128.48, 128.45, 124.1, 79.6, 53.0; HRMS (FD) m/z: $[M]^+$ Calcd for $C_{28}H_{18}O_5$ 434.1149; Found 434.1147; FT-IR (KBr) ṽ (cm$^{-1}$) 2917, 2850, 1733, 1707, 1647, 1468, 1441 cm$^{-1}$.

**Ethyl 2-(2-(4-cyanophenyl)-1',3'-dioxo-3-phenyl-1',3'-dihydrospiro[cyclopentane-1,2'-indene]-2,4-dien-5-yl)-2-oxoacetate (4b)**

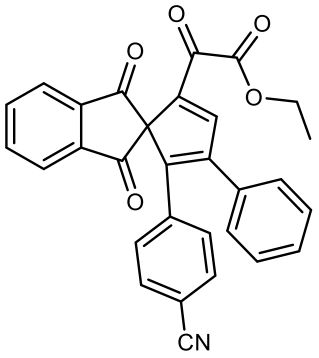

Yield 89% (5.9 mg), yellow solid, m.p. 214−217 °C; $R_f$ = 0.40 (hexanes/EA = 9:1); $^1$H NMR ($CDCl_3$, 400 MHz) δ 8.50 (s, 1H), 7.98 (dd, *J* = 5.7, 3.1 Hz, 2H), 7.85 (dd, *J* = 5.7, 3.0 Hz, 2H), 7.39–7.34 (m, 2H), 7.32–7.22 (m, 5H), 7.11–7.03 (m, 2H), 4.33 (q, *J* = 7.2 Hz, 2H), 1.35 （t, *J* = 7.2 Hz, 3H) ppm; $^{13}$C NMR ($CDCl_3$, 100 MHz) δ 194.4, 176.9, 160.4, 155.6, 148.8, 146.7, 143.5, 143.2, 138.0, 136.1, 132.4, 132.3, 129.4, 129.0, 128.8, 128.4, 124.3, 118.0, 112.4, 79.4, 62.6, 13.9 ppm; FT-IR (KBr) $\tilde{\nu}$ (cm$^{-1}$) 2982, 1732, 1708, 1651, 1127; HRMS (ESI) m/z: $[M + H]^+$ Calcd for $C_{30}H_{20}NO_5$ 474.1341; Found 474.1336.

**Methyl 2-(3-(4-(*tert*-butyl)phenyl)-2-(4-cyanophenyl)-1',3'-dioxo-1',3'-dihydrospiro[cyclopentane-**

**1,2'-indene]-2,4-dien-5-yl)-2-oxoacetate (4c)**

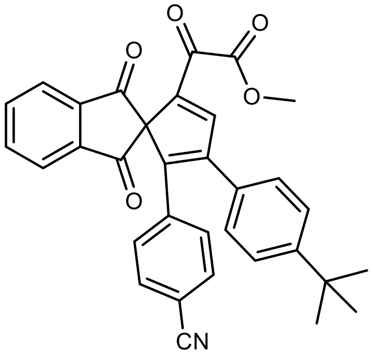

Yield 91% (6.6 mg), yellow solid, m.p. 118−120 ºC; $R_f$ = 0.28 (hexanes/EA = 7:3); $^1$H NMR ($CDCl_3$, 400 MHz) δ 8.50 (s, 1H), 7.97 (dd, $J$ = 5.7, 3.0 Hz, 2H), 7.84 (dd, $J$ = 5.7, 3.1 Hz, 2H), 7.40–7.35 (m, 2H), 7.32 7.27 (m, 2H), 7.19–7.14 (m, 2H), 7.12–7.07 (m, 2H), 3.87 (s, 3H), 1.28 (s, 9H) ppm; $^{13}$C NMR ($CDCl_3$, 100 MHz) δ 194.5, 176.6, 160.9, 156.0, 152.3, 148.4, 146.1, 143.2, 138.3, 136.1, 132.4, 129.4, 129.2, 128.1, 125.7, 124.2, 118.1, 112.3, 79.4, 53.0, 34.7, 31.1 ppm (one fewer carbon was observed); FT-IR (KBr) $\tilde{\nu}$ ($cm^{-1}$) 2961, 1739, 1707, 1654, 1162; HRMS (ESI) m/z: $[M+H]^+$ Calcd for $C_{33}H_{26}NO_5$ 516.1811; Found 516.1806.

## Spectra for all new compounds

**Figure S1.** $^{1}$H-NMR spectrum of compound **3a** ($CDCl_3$, 400 MHz)

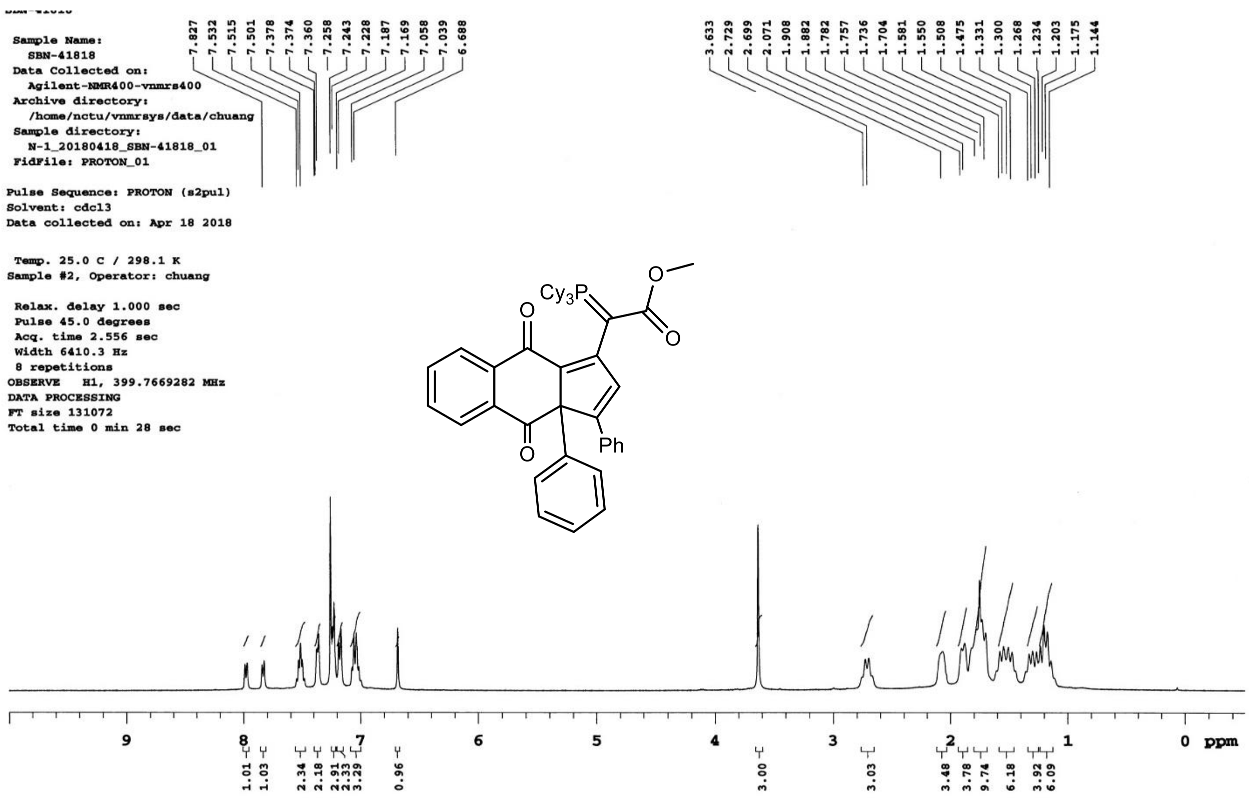


**Figure S2.** $^{13}$C-NMR spectrum of compound **3a** ($CDCl_3$, 100 MHz)

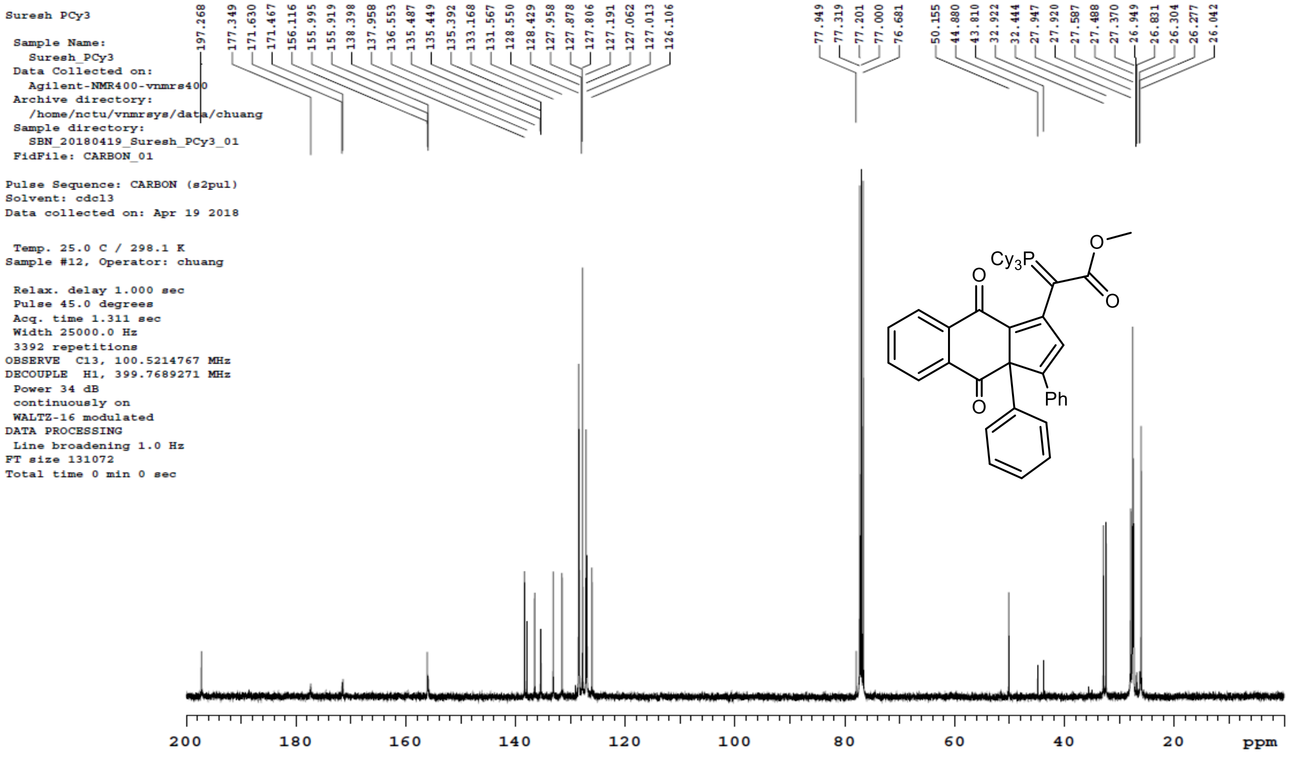

**Figure S3.** $^{1}$H-NMR spectrum of compound **3b** ($CDCl_3$, 400 MHz)

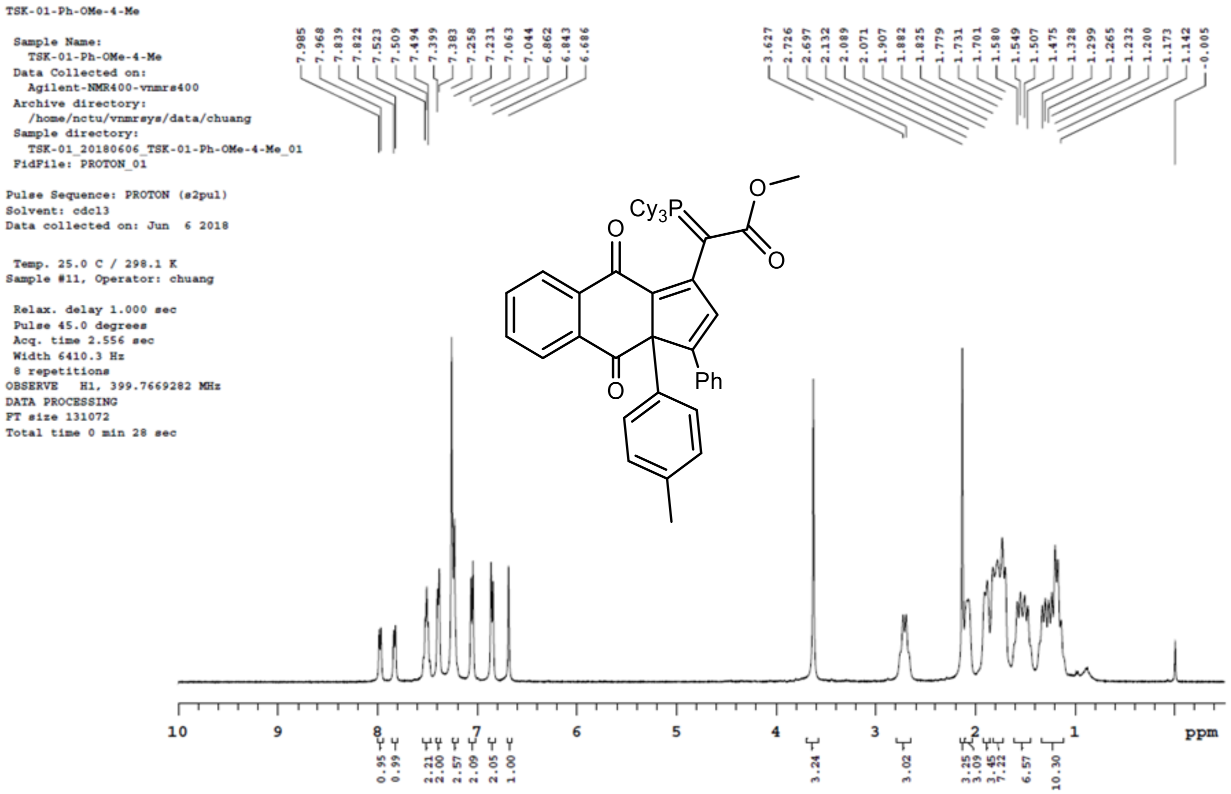

**Figure S4.** $^{13}$C-NMR spectrum of compound **3b** ($CDCl_3$, 100 MHz)

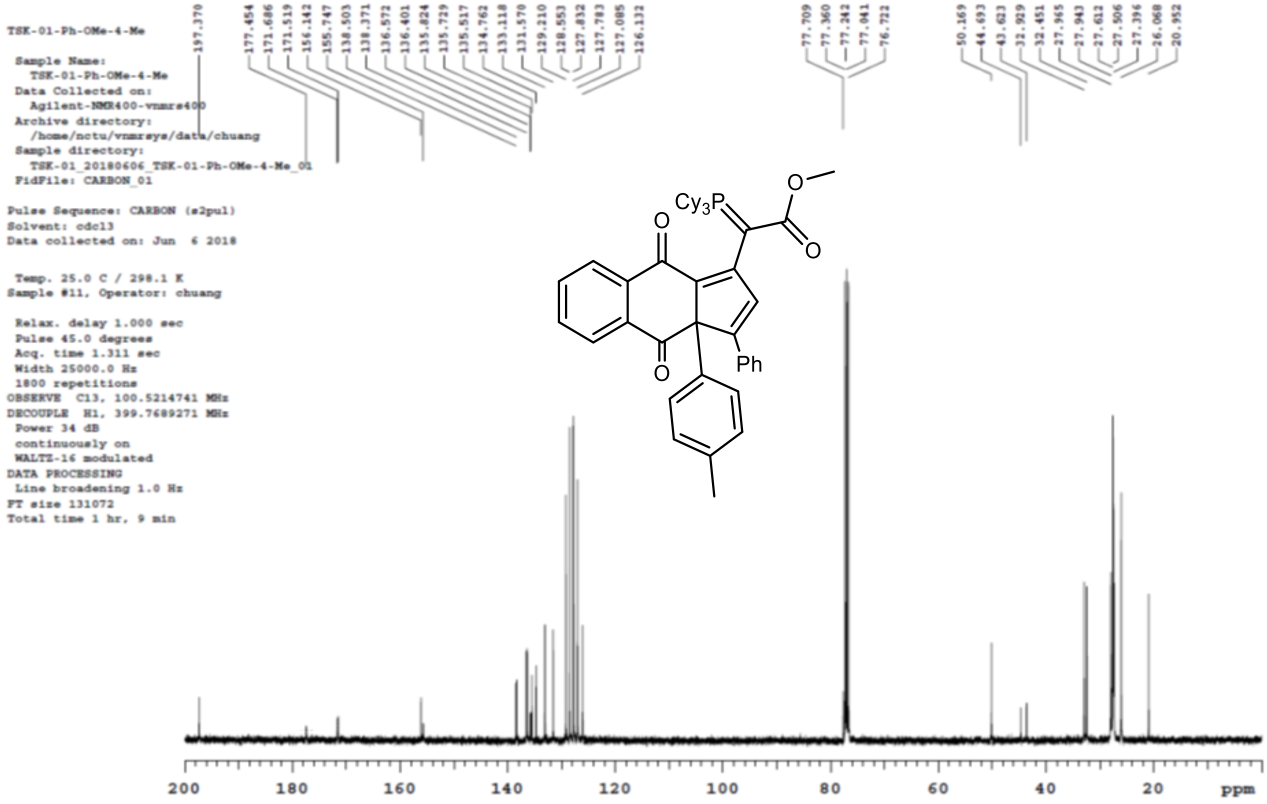

**Figure S5.** $^{1}$H-NMR spectrum of compound **3c** ($CDCl_3$, 400 MHz)

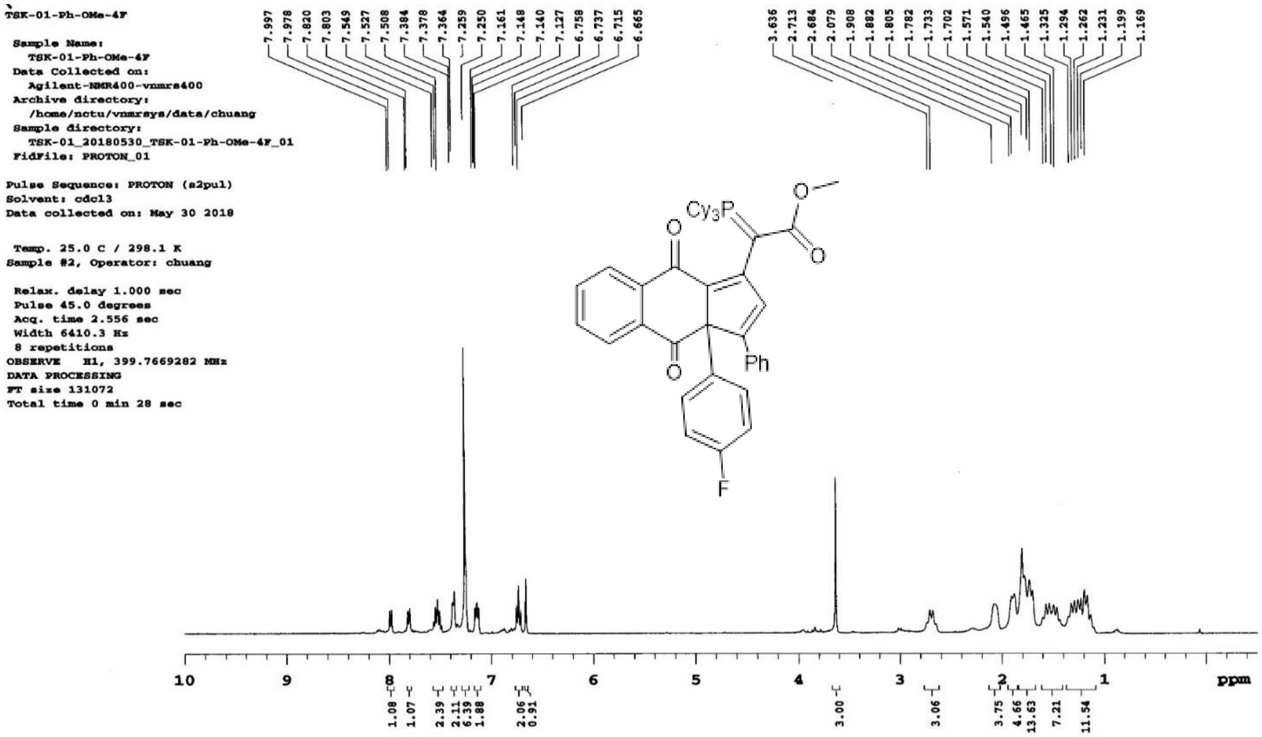

**Figure S6.** $^{13}$C-NMR spectrum of compound **3c** ($CDCl_3$, 100 MHz)

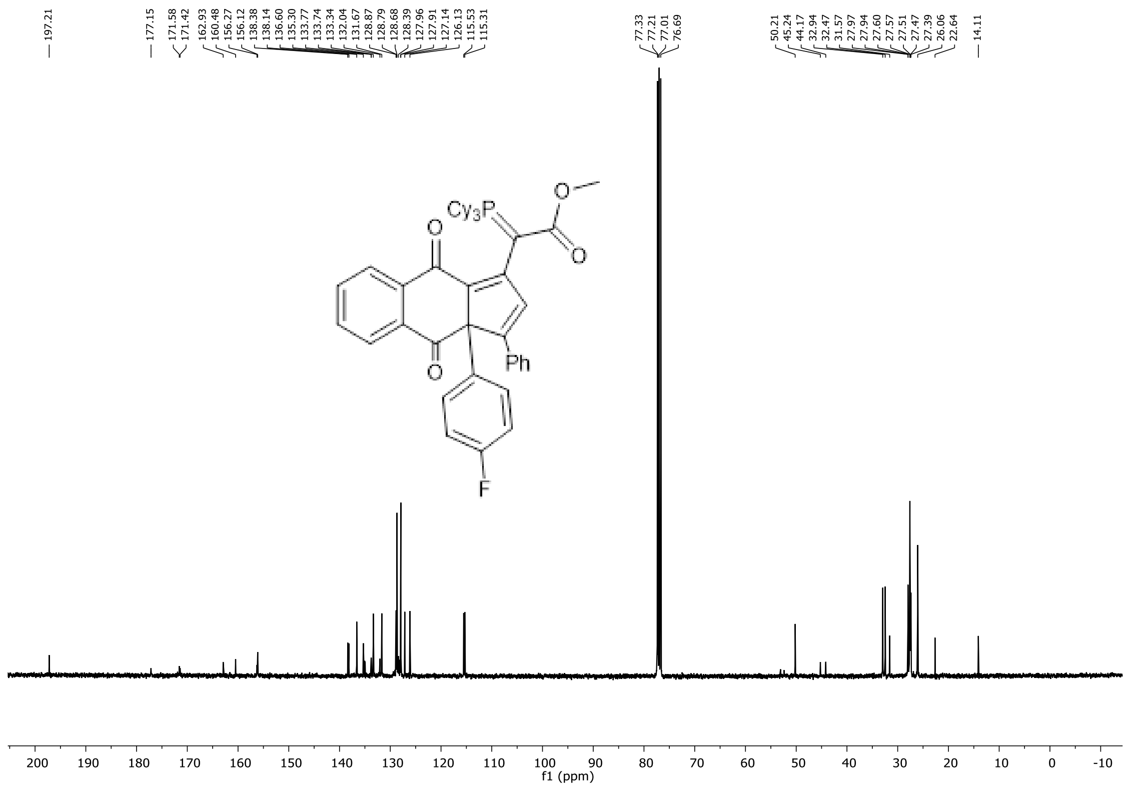

**Figure S7.** $^{1}$H-NMR spectrum of compound **3d** ($CDCl_3$, 400 MHz)

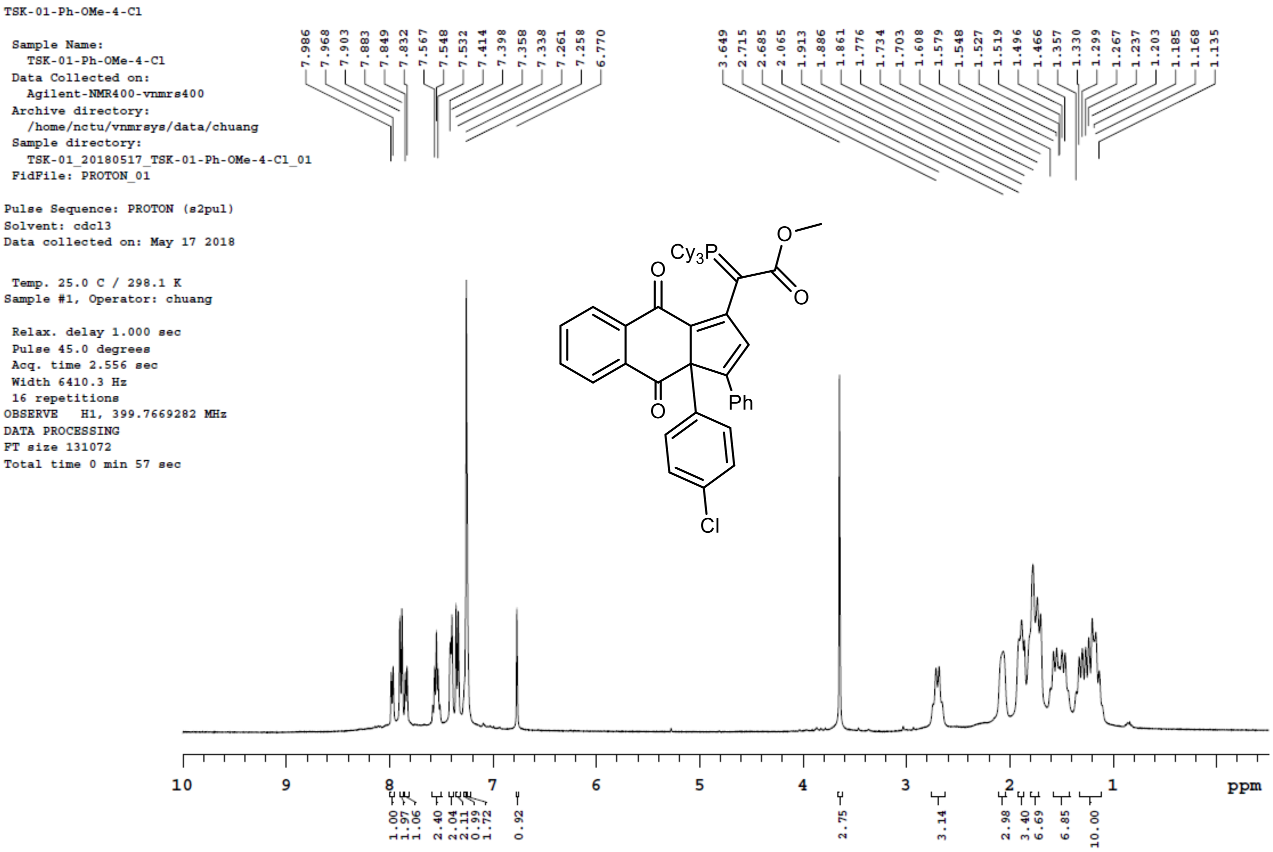


**Figure S8.** $^{13}$C-NMR spectrum of compound **3d** ($CDCl_3$, 100 MHz)

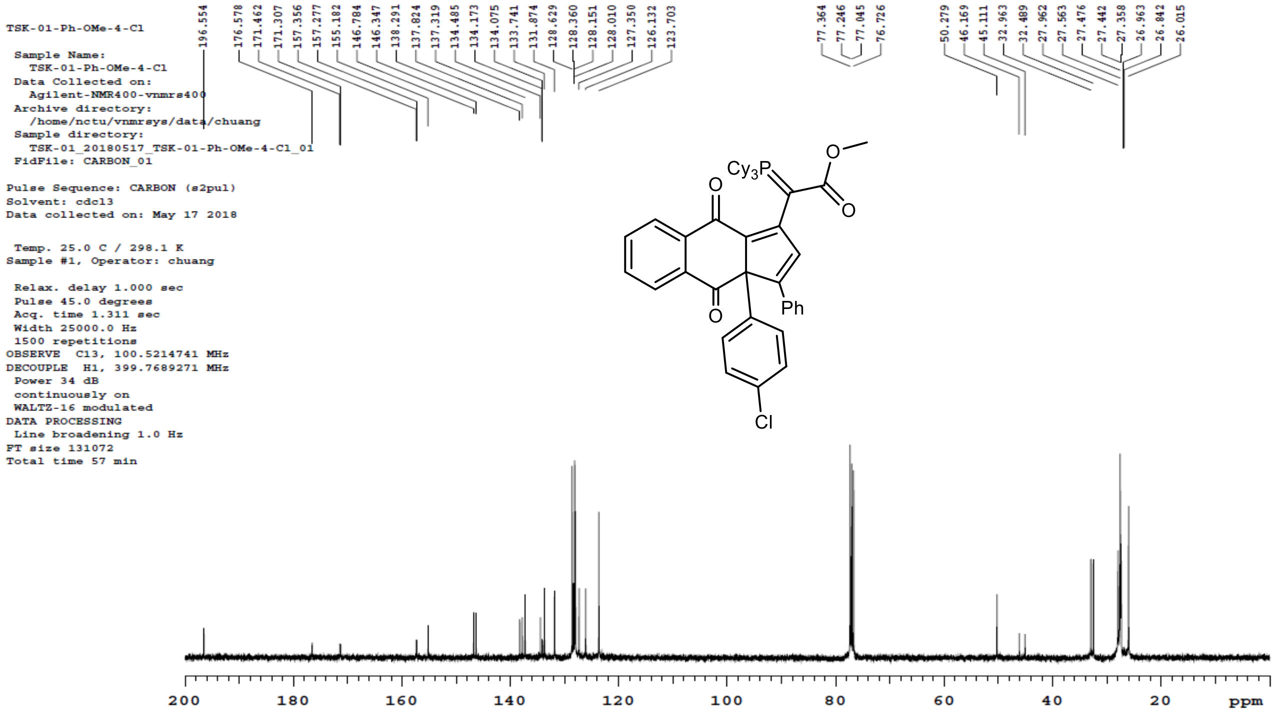


**Figure S9.** $^{1}$H-NMR spectrum of compound **3e** ($CDCl_3$, 400 MHz)

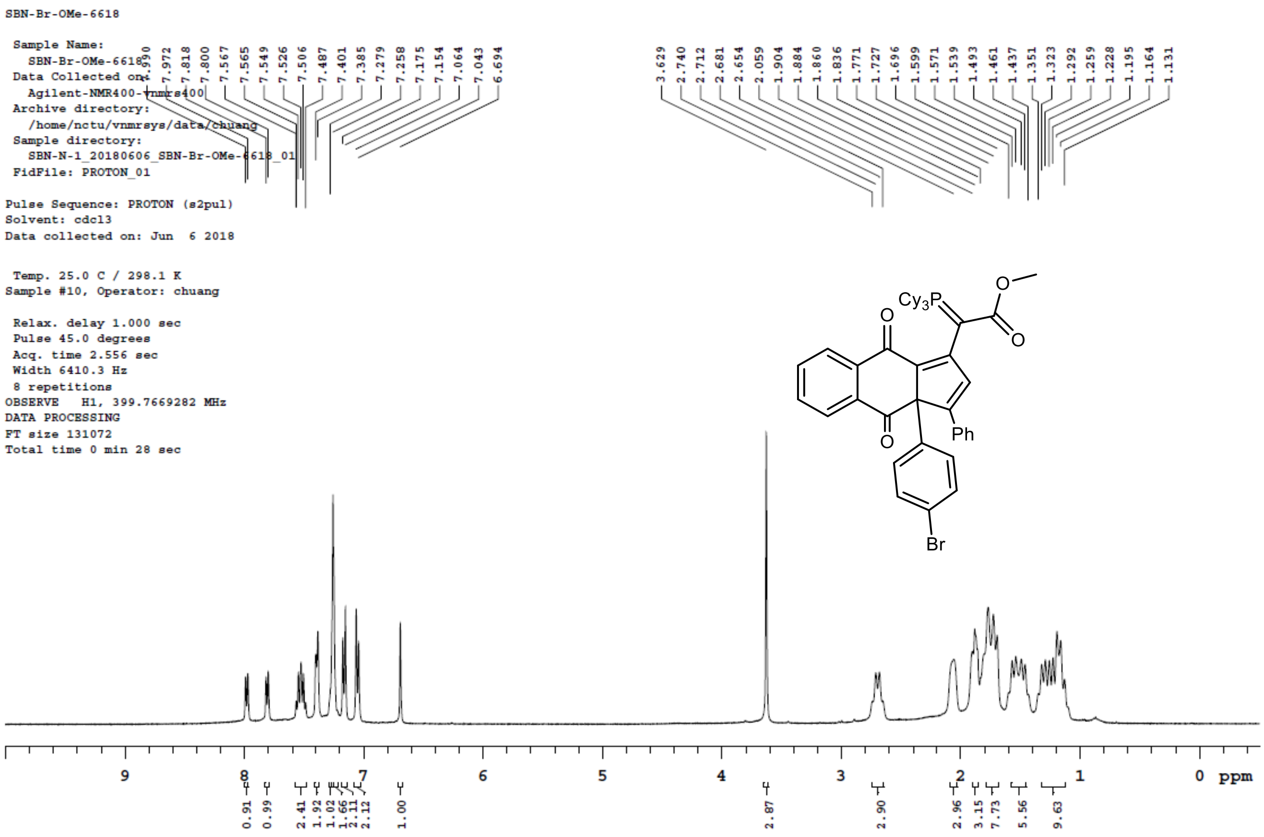


**Figure S10.** $^{13}$C-NMR spectrum of compound **3e** ($CDCl_3$, 100 MHz)

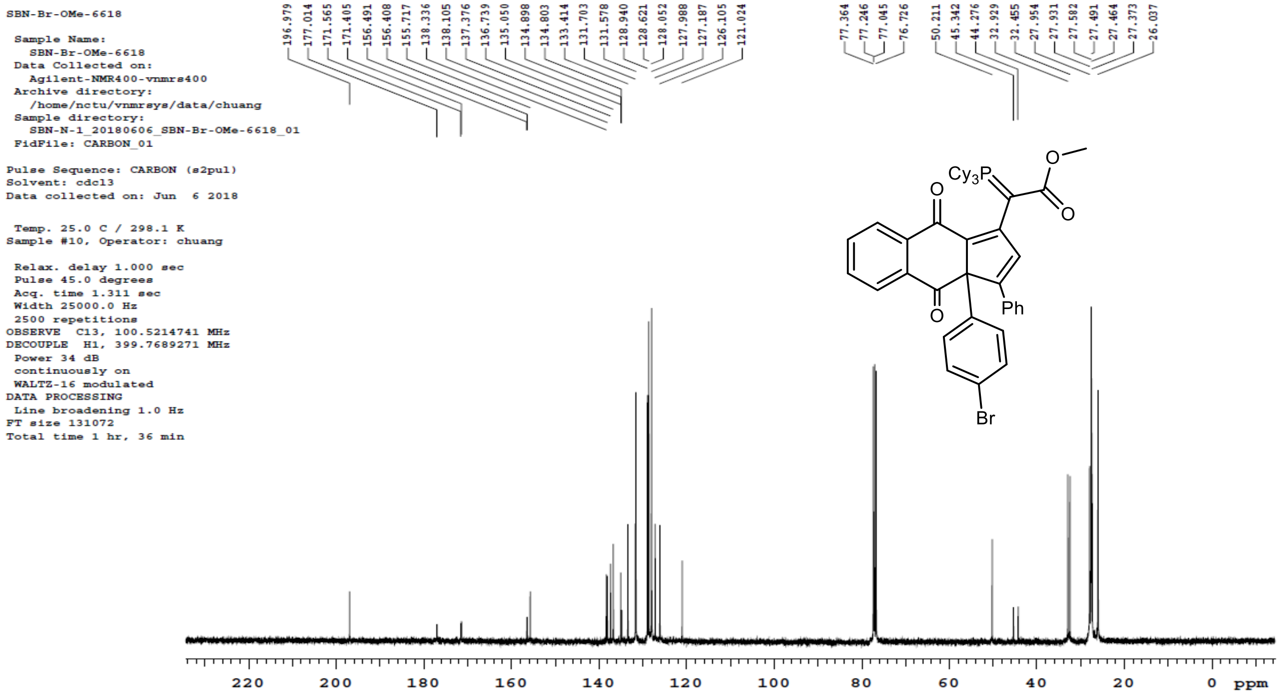

**Figure S11.** $^{1}$H-NMR spectrum of compound **3f** ($CDCl_3$, 400 MHz)

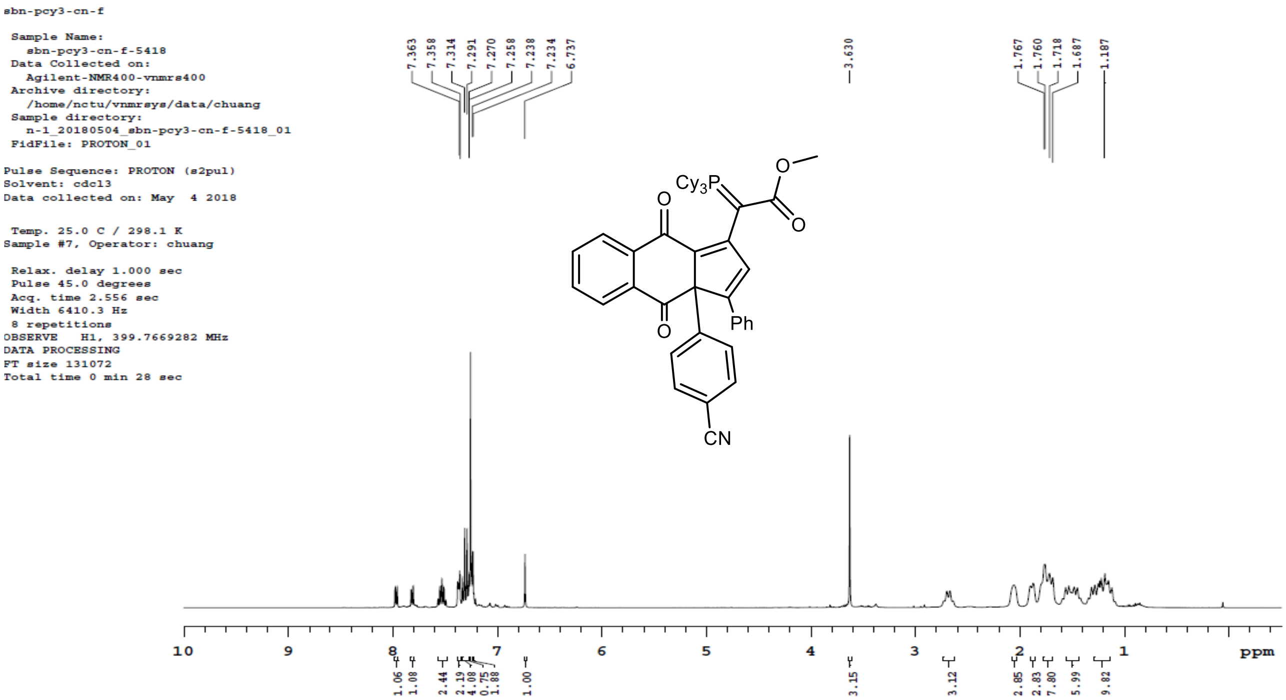


**Figure S12.** $^{13}$C-NMR spectrum of compound **3f** ($CDCl_3$, 100 MHz)

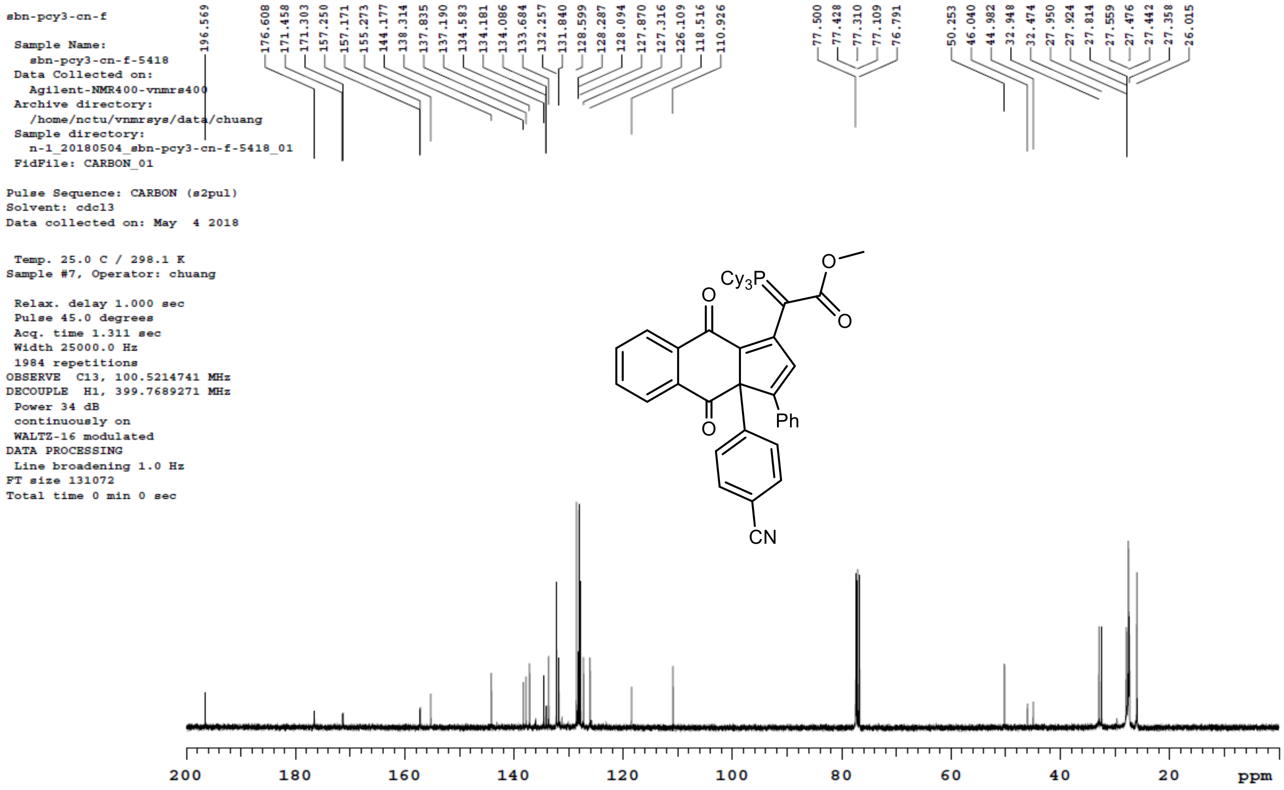

**Figure S13.** $^{1}$H-NMR spectrum of compound **3g** ($CDCl_3$, 400 MHz)

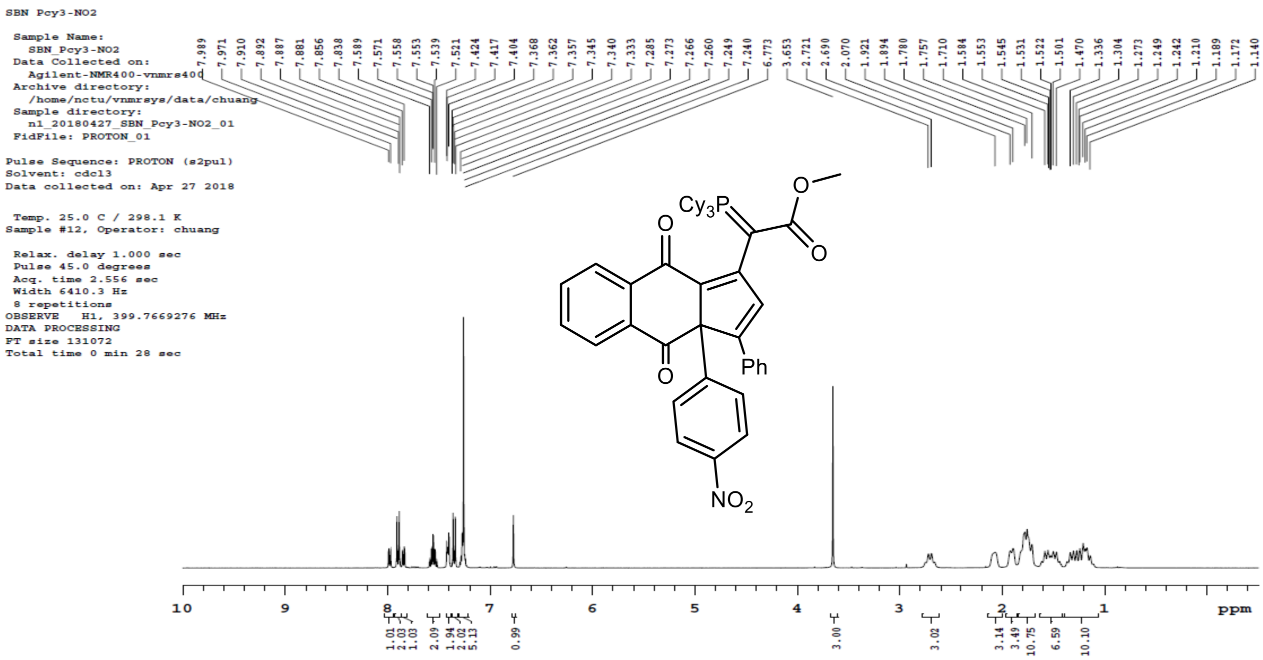


**Figure S14.** $^{13}$C-NMR spectrum of compound **3g** ($CDCl_3$, 100 MHz)

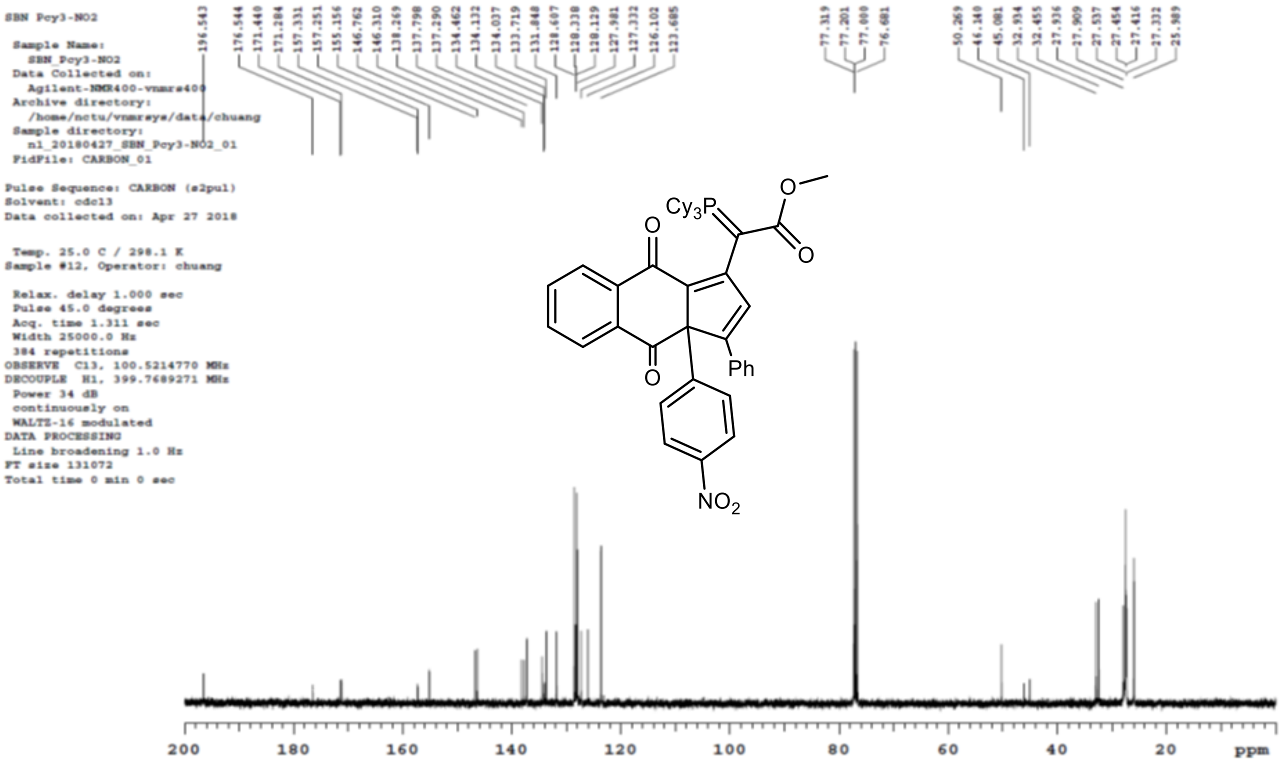

**Figure S15.** $^{1}$H-NMR spectrum of compound **3h** ($CDCl_3$, 400 MHz)

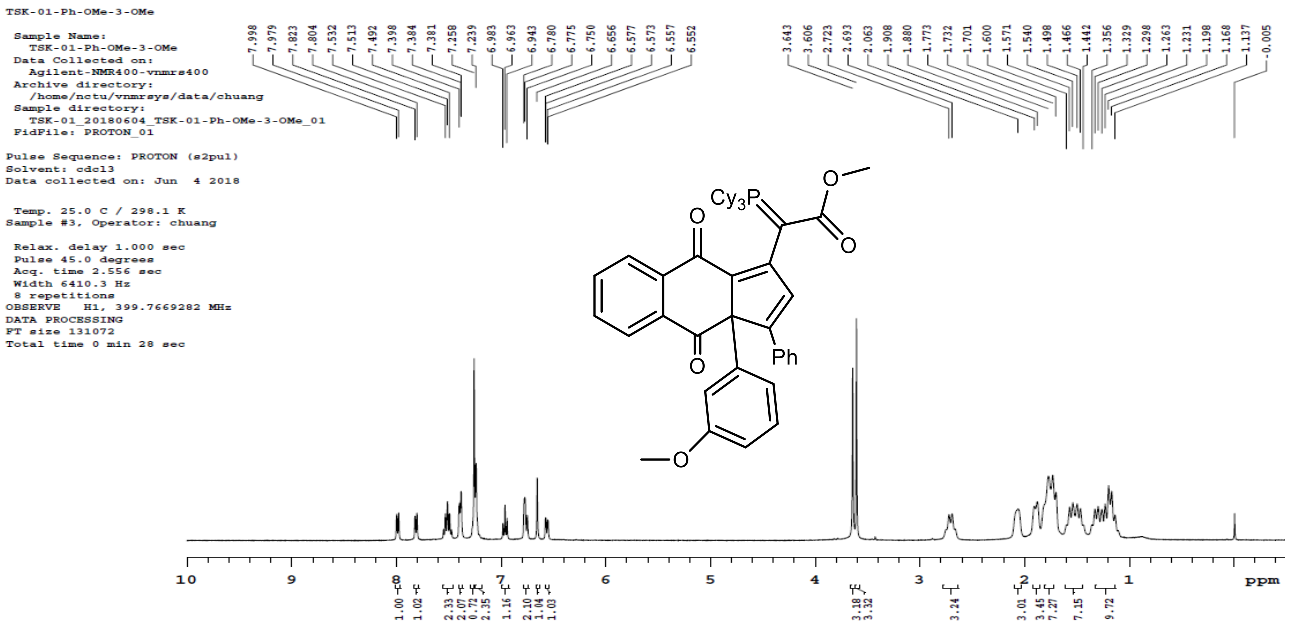

**Figure S16.** $^{13}$C-NMR spectrum of compound **3h** ($CDCl_3$, 100 MHz)

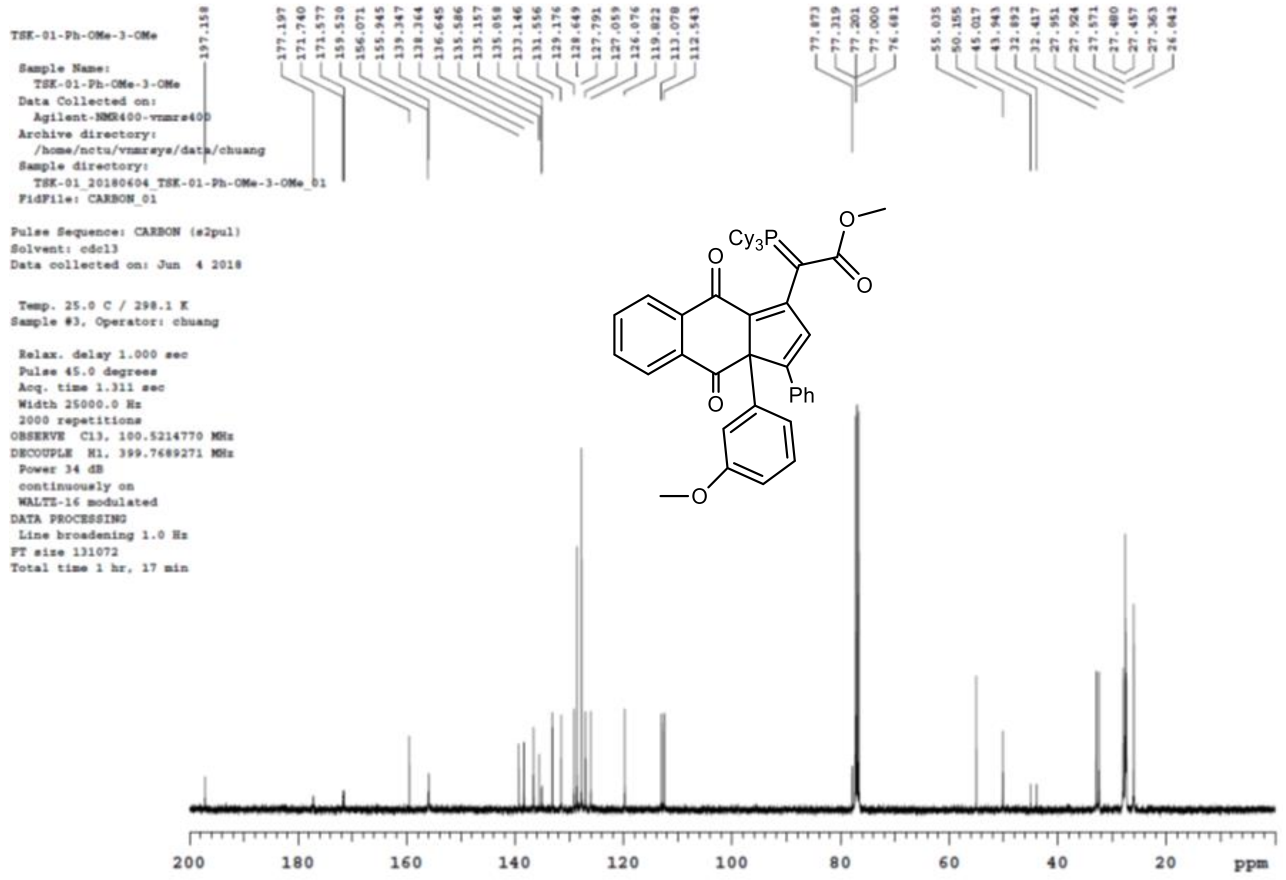

**Figure S17.** $^1$H-NMR spectrum of compound **3i** ($CDCl_3$, 400 MHz)

| | Parameter | Value |
|---|---|---|
| 1 | Title | PROTON_01 |
| 2 | Comment | SBN-PC-18-01 |
| 3 | Origin | Varian |
| 4 | Spectrometer | vnmrs |
| 5 | Solvent | cdcl3 |
| 6 | Temperature | 25.0 |
| 7 | Pulse Sequence | s2pul |
| 8 | Experiment | 1D |
| 9 | Probe | Oneprobe |
| 10 | Relaxation Delay | 1.0000 |
| 11 | Pulse Width | 5.1000 |
| 12 | Presaturation Frequency | |
| 13 | Acquisition Time | 2.5559 |
| 14 | Spectrometer Frequency | 399.77 |
| 15 | Spectral Width | 6410.3 |
| 16 | Lowest Frequency | -806.6 |
| 17 | Nucleus | 1H |
| 18 | Acquired Size | 16384 |
| 19 | Spectral Size | 65536 |

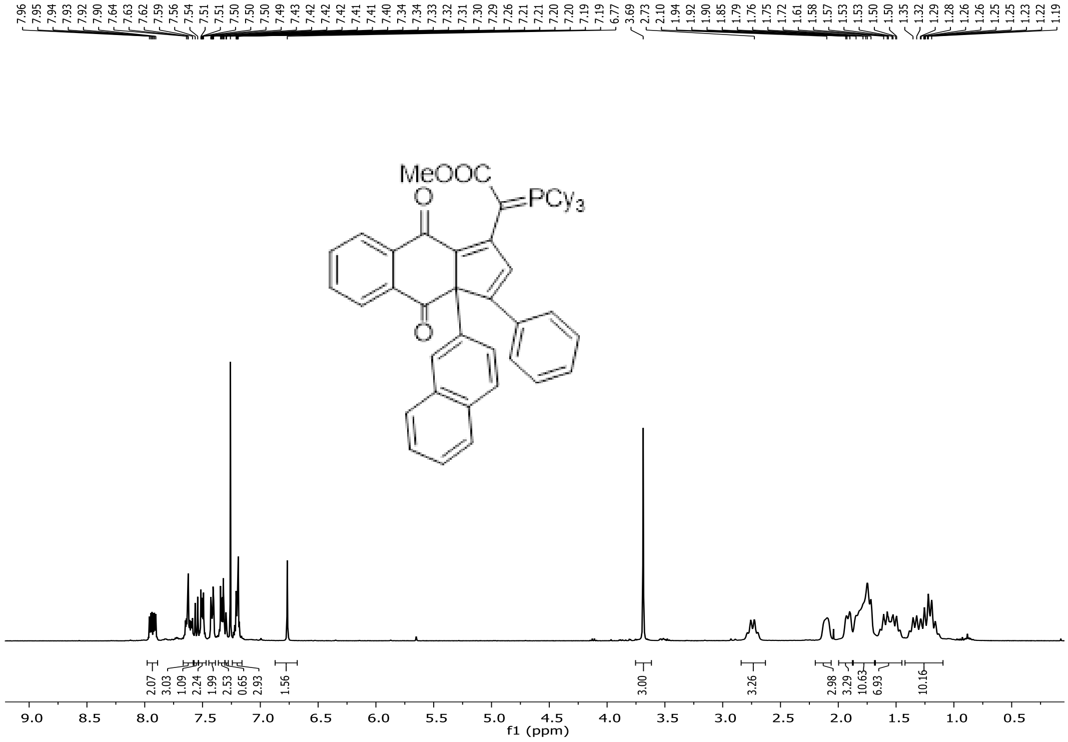


**Figure S18.** $^{13}$C-NMR spectrum of compound **3i** ($CDCl_3$, 100 MHz)

| | Parameter | Value |
|---|---|---|
| 1 | Title | CARBON_01 |
| 2 | Origin | Varian |
| 3 | Spectrometer | vnmrs |
| 4 | Solvent | cdcl3 |
| 5 | Temperature | 25.0 |
| 6 | Pulse Sequence | s2pul |
| 7 | Experiment | 1D |
| 8 | Probe | Oneprobe |
| 9 | Relaxation Delay | 1.0000 |
| 10 | Pulse Width | 5.2500 |
| 11 | Presaturation Frequency | |
| 12 | Acquisition Time | 1.3107 |
| 13 | Spectrometer Frequency | 100.53 |
| 14 | Spectral Width | 25000.0 |
| 15 | Lowest Frequency | -1442.7 |
| 16 | Nucleus | 13C |
| 17 | Acquired Size | 32768 |
| 18 | Spectral Size | 65536 |

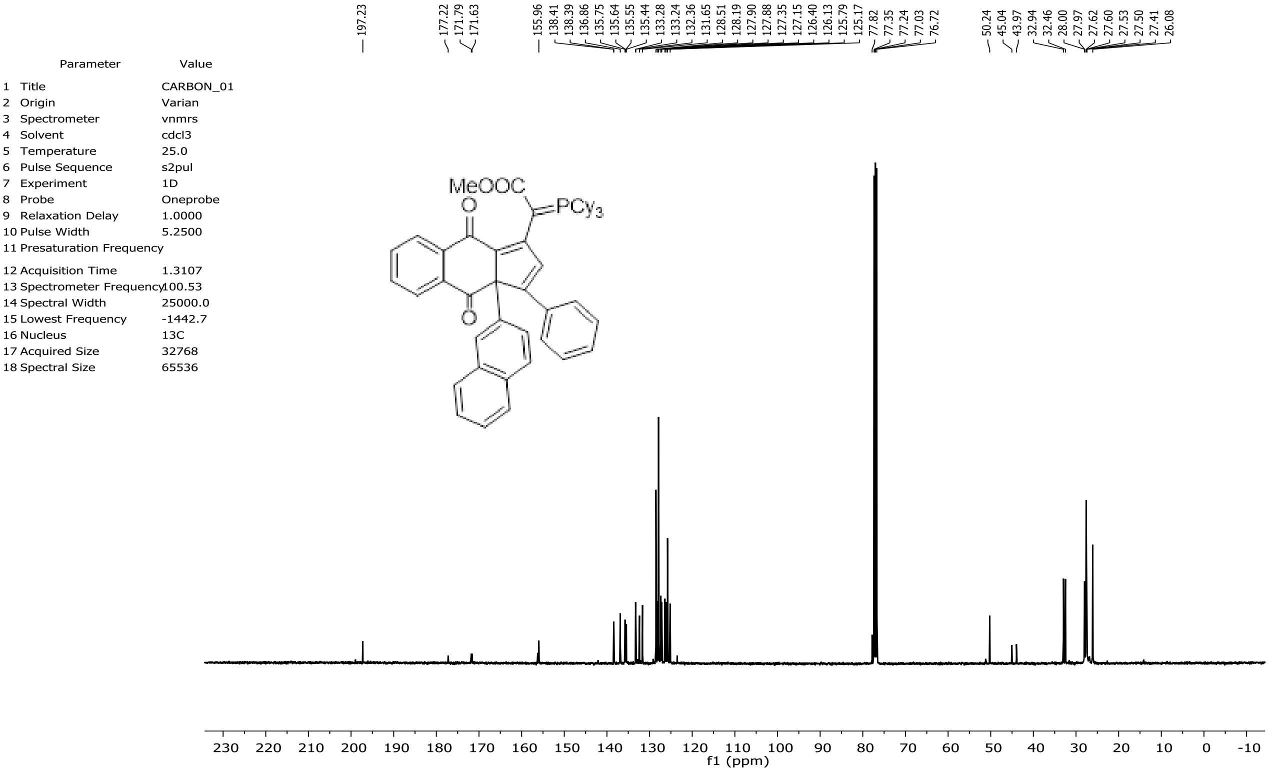

**Figure S19.** $^{1}$H-NMR spectrum of compound **3j** ($CDCl_3$, 400 MHz)

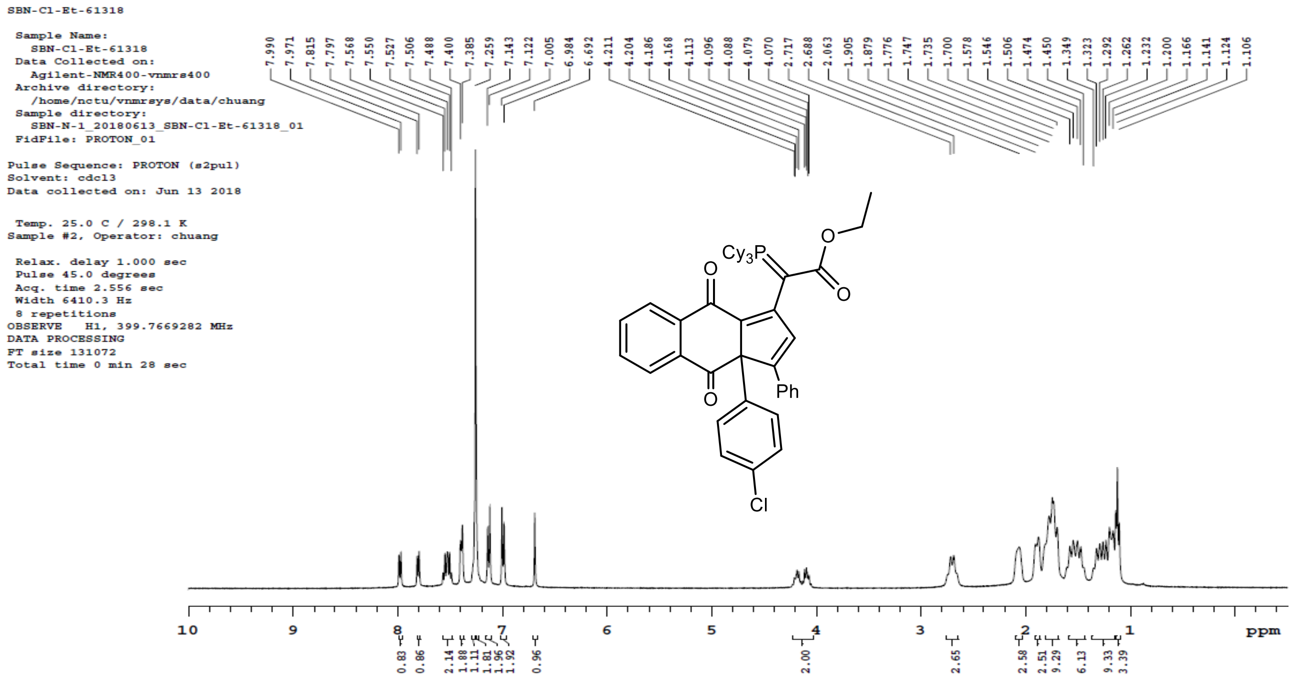


**Figure S20.** $^{13}$C-NMR spectrum of compound **3j** ($CDCl_3$, 100 MHz)

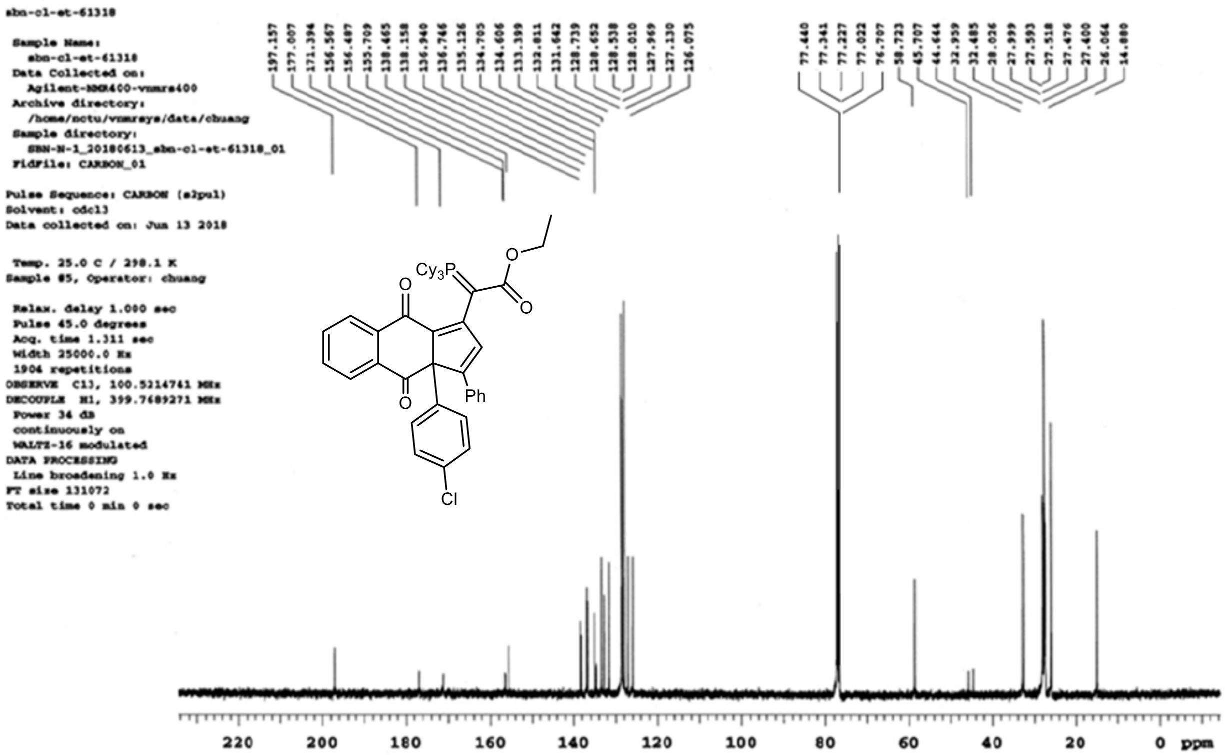

**Figure S21.** $^{1}$H-NMR spectrum of compound **3k** ($CDCl_3$, 400 MHz)

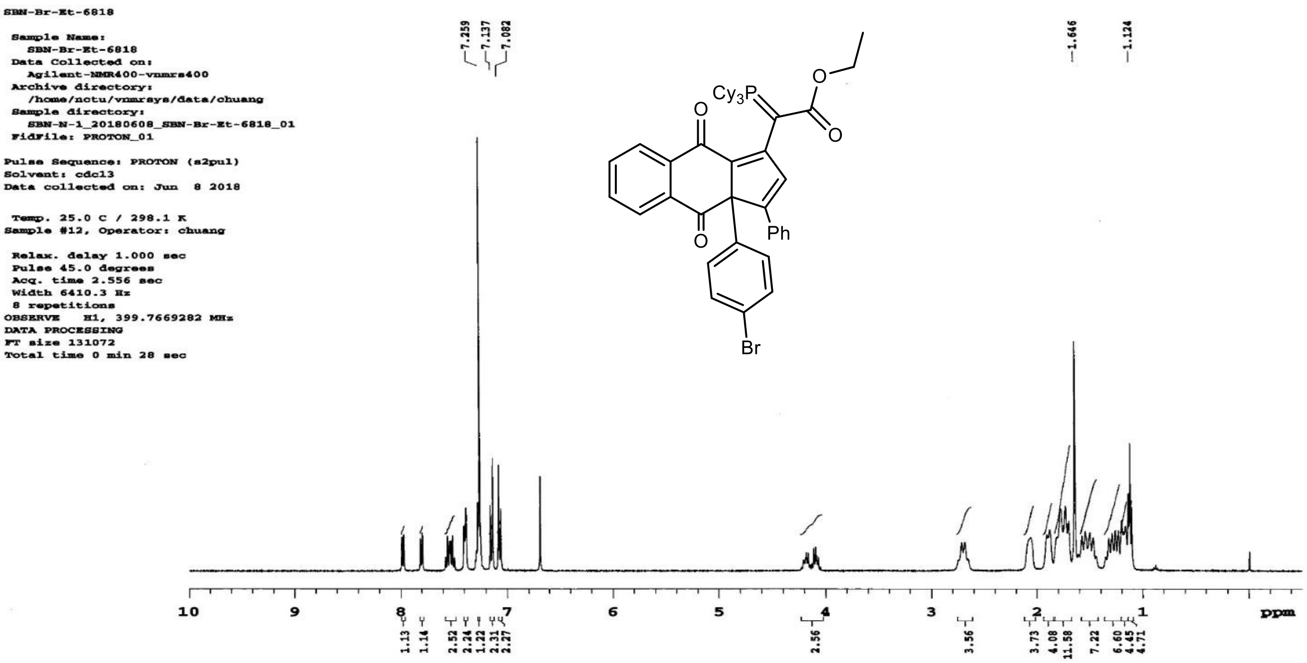


**Figure S22.** $^{13}$C-NMR spectrum of compound **3k** ($CDCl_3$, 100 MHz)

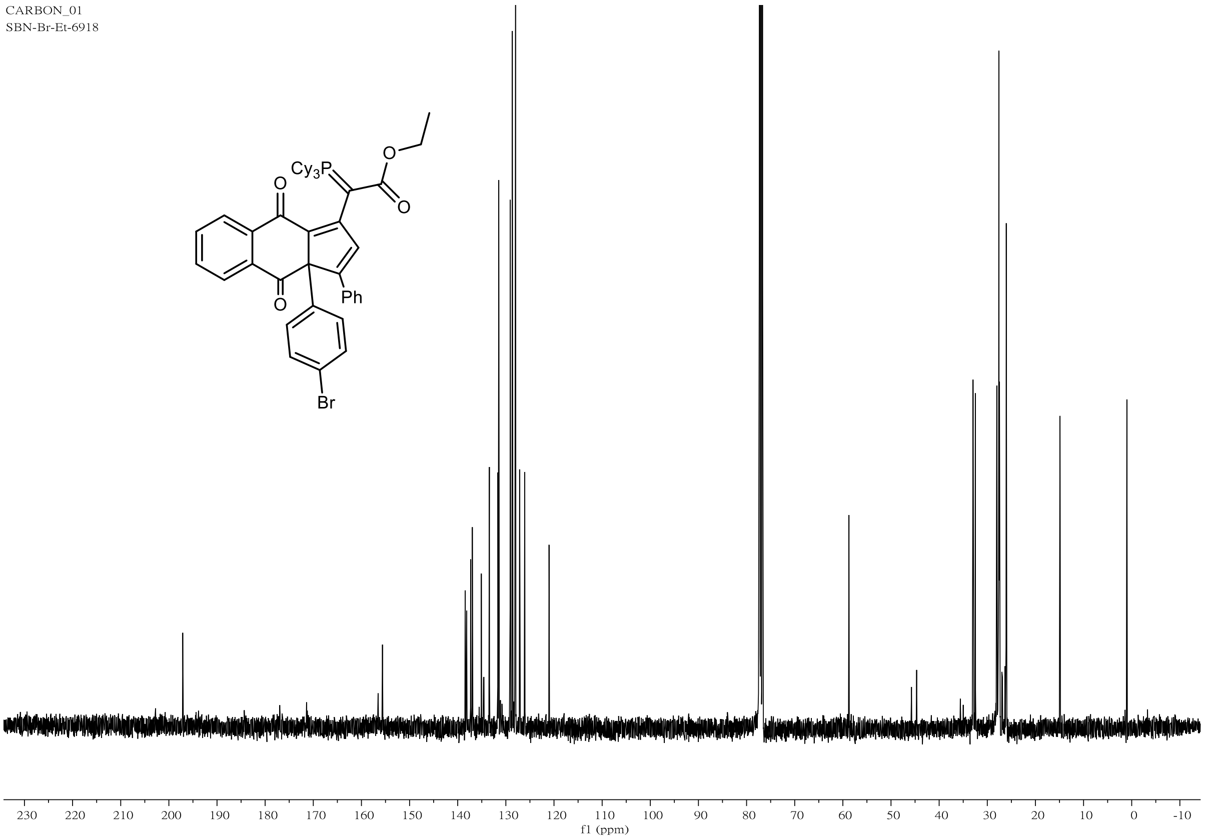

**Figure S23.** $^{1}$H-NMR spectrum of compound **3l** ($CDCl_3$, 400 MHz)

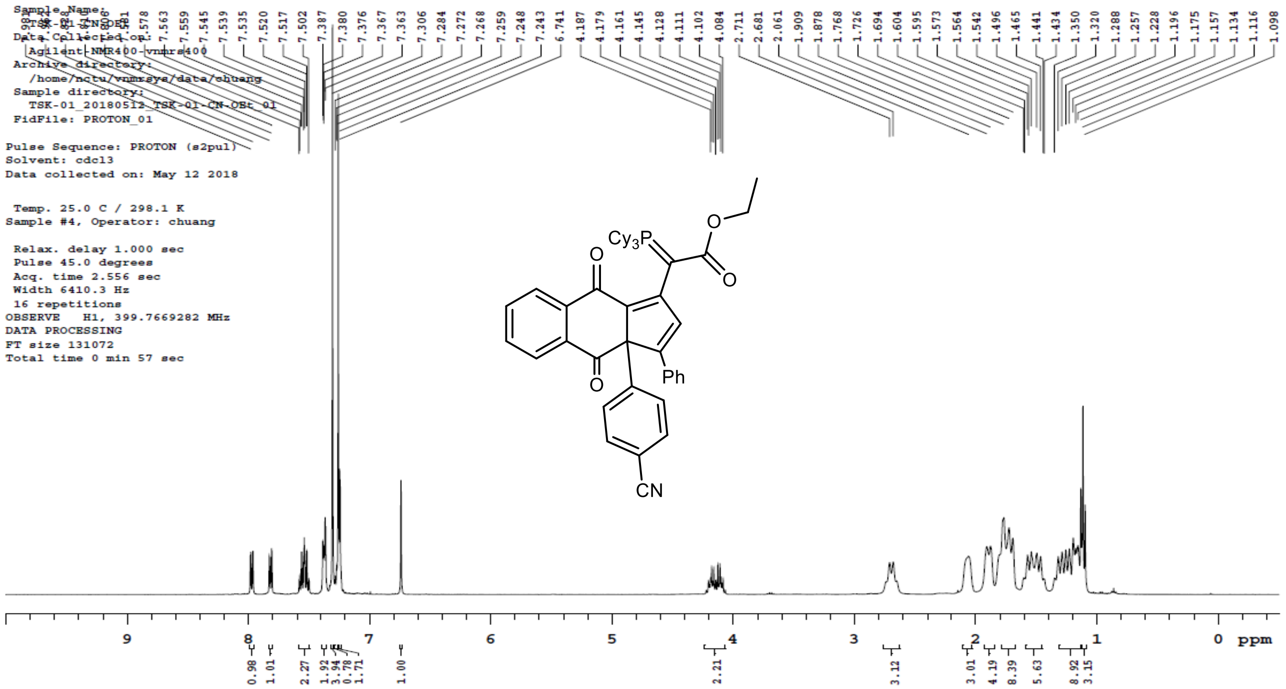


**Figure S24.** $^{13}$C-NMR spectrum of compound **3l** ($CDCl_3$, 100 MHz)

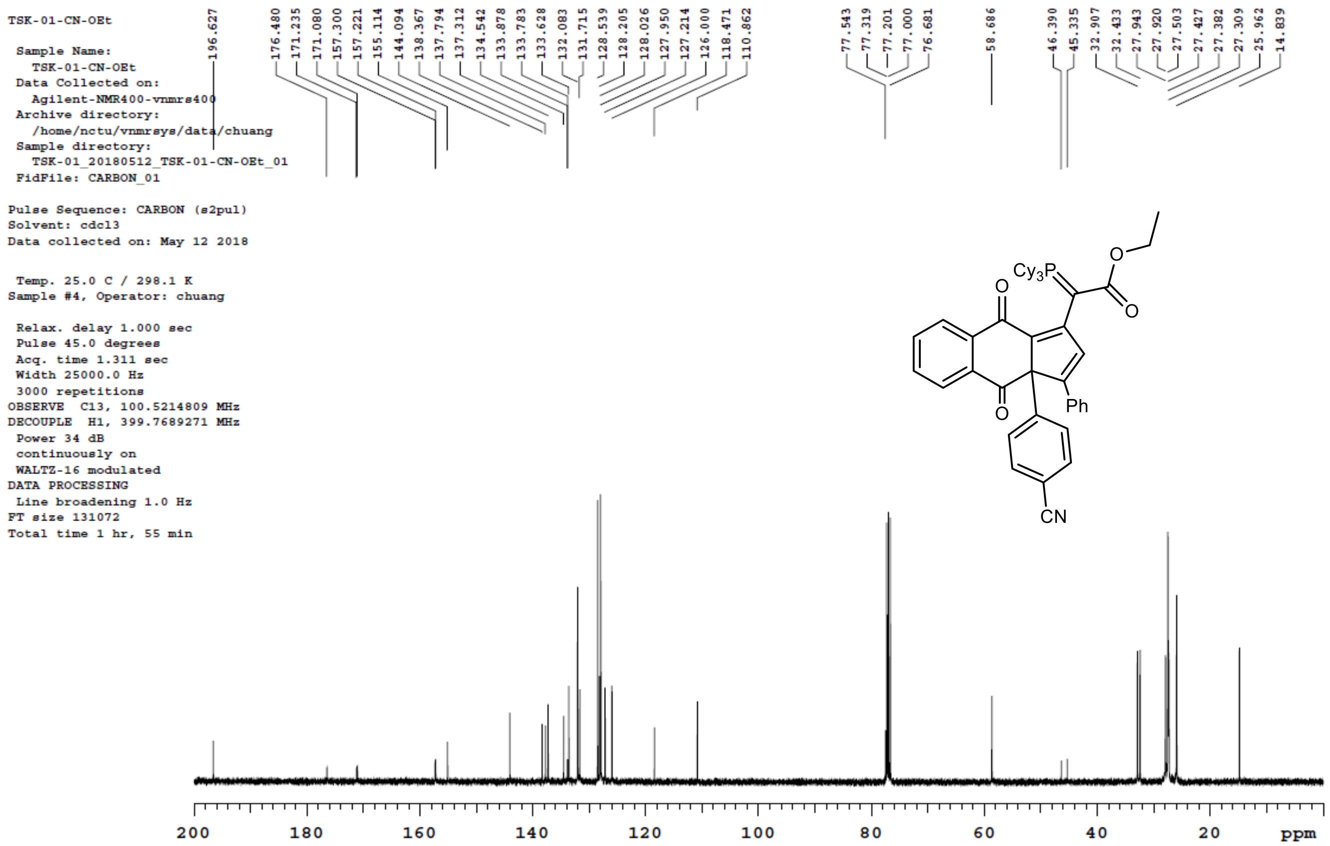

**Figure S25.** $^{1}$H-NMR spectrum of compound **3m** ($CDCl_3$, 400 MHz)

| | Parameter | Value |
|---|---|---|
| 1 | Title | PROTON_01 |
| 2 | Comment | SBN-PCy3-t-Bu |
| 3 | Origin | Varian |
| 4 | Spectrometer | vnmrs |
| 5 | Solvent | cdcl3 |
| 6 | Temperature | 25.0 |
| 7 | Pulse Sequence | s2pul |
| 8 | Experiment | 1D |
| 9 | Probe | Oneprobe |
| 10 | Relaxation Delay | 1.0000 |
| 11 | Pulse Width | 5.1000 |
| 12 | Presaturation Frequency | |
| 13 | Acquisition Time | 2.5559 |
| 14 | Spectrometer Frequency | 399.77 |
| 15 | Spectral Width | 6410.3 |
| 16 | Lowest Frequency | -806.6 |
| 17 | Nucleus | 1H |
| 18 | Acquired Size | 16384 |
| 19 | Spectral Size | 65536 |

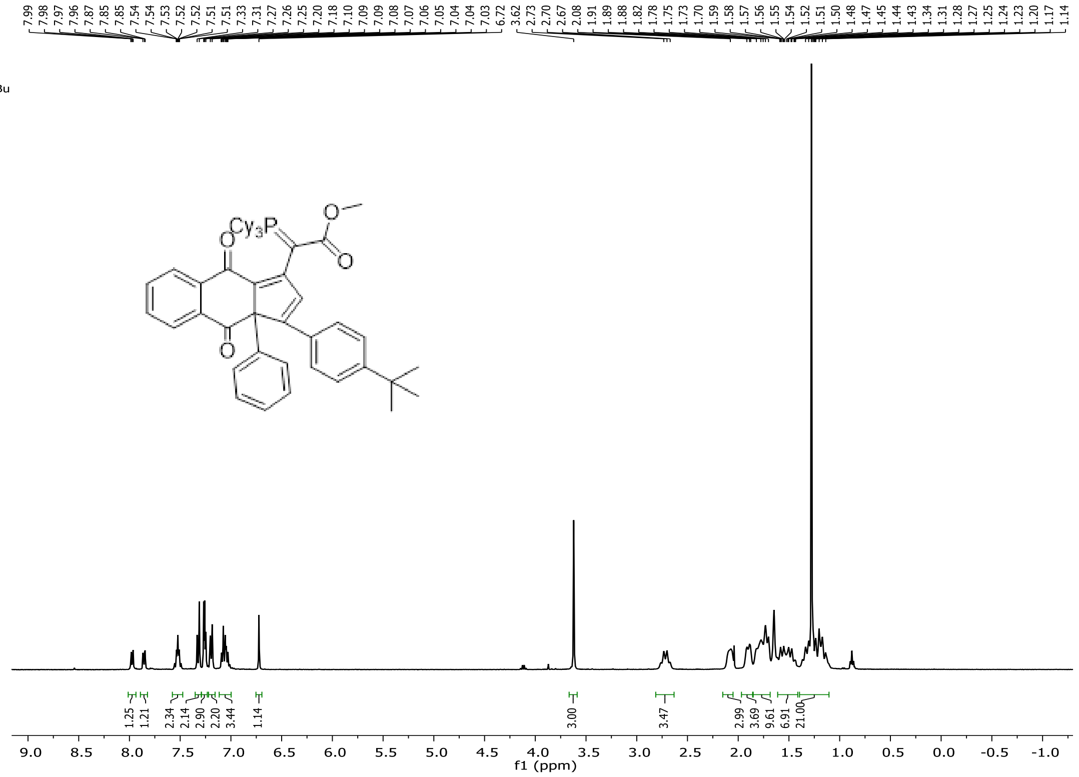


**Figure S26.** $^{13}$C-NMR spectrum of compound **3m** ($CDCl_3$, 100 MHz)

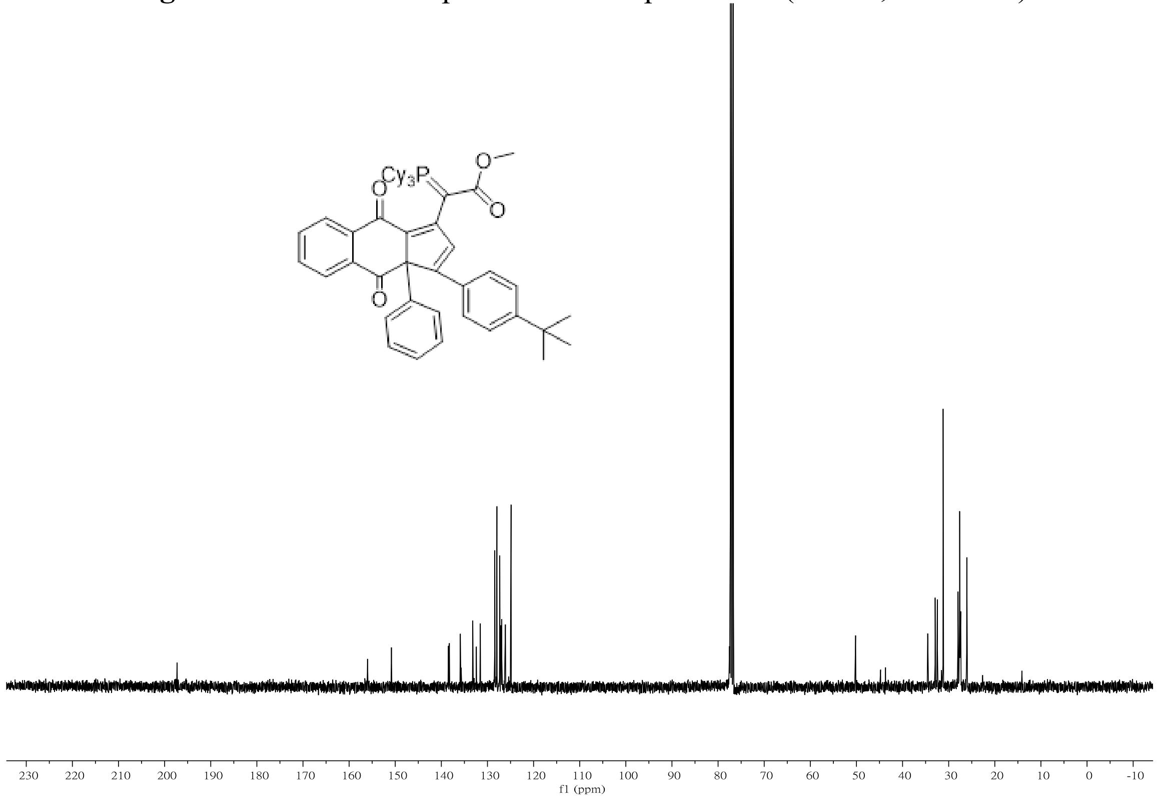

**Figure S27.** $^{1}$H-NMR spectrum of compound **3n** ($CDCl_3$, 400 MHz)

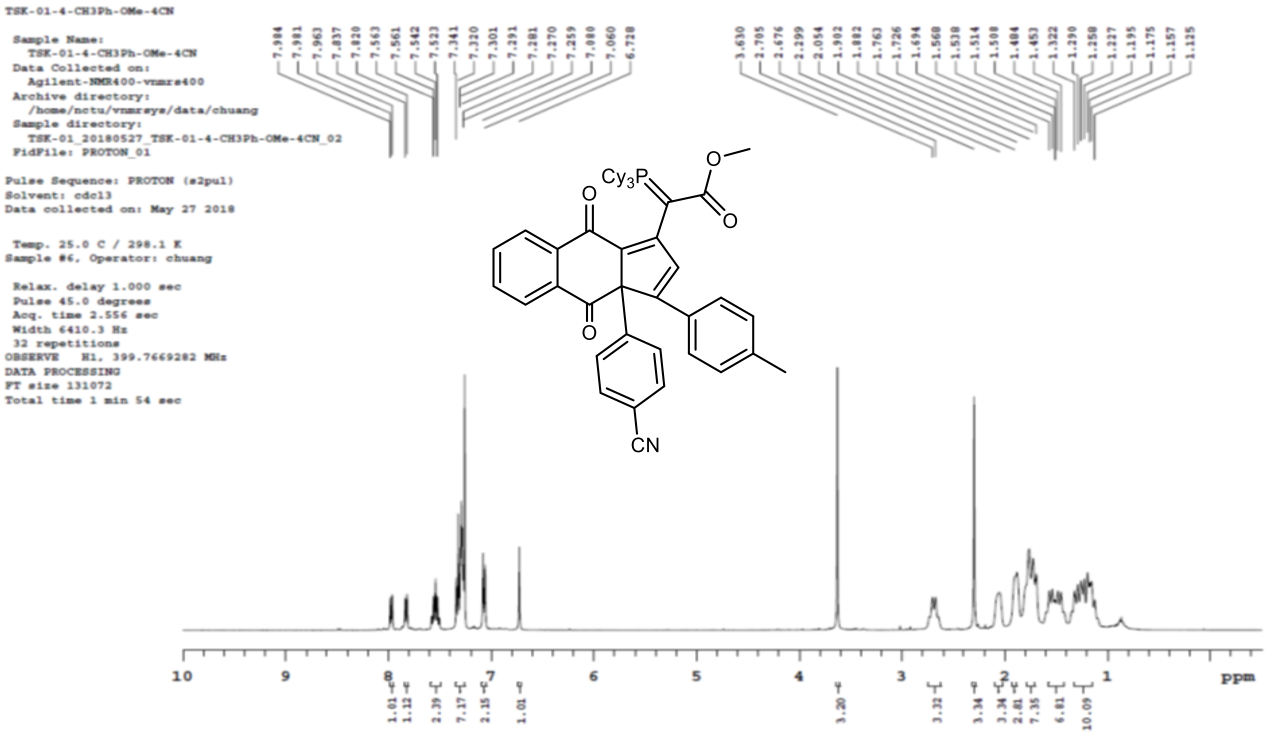

**Figure S28.** $^{13}$C-NMR spectrum of compound **3n** ($CDCl_3$, 100 MHz)

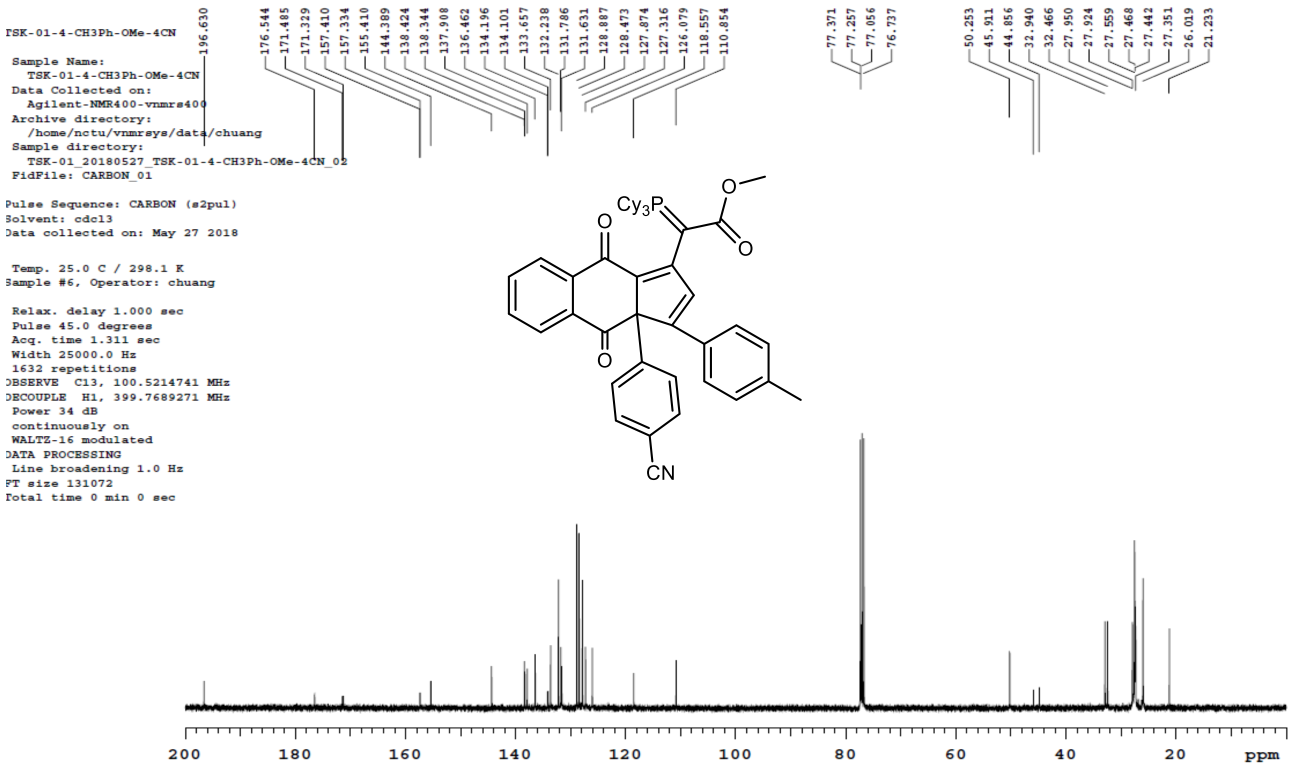

**Figure S29.** $^{1}$H-NMR spectrum of compound **3o** ($CDCl_3$, 400 MHz)

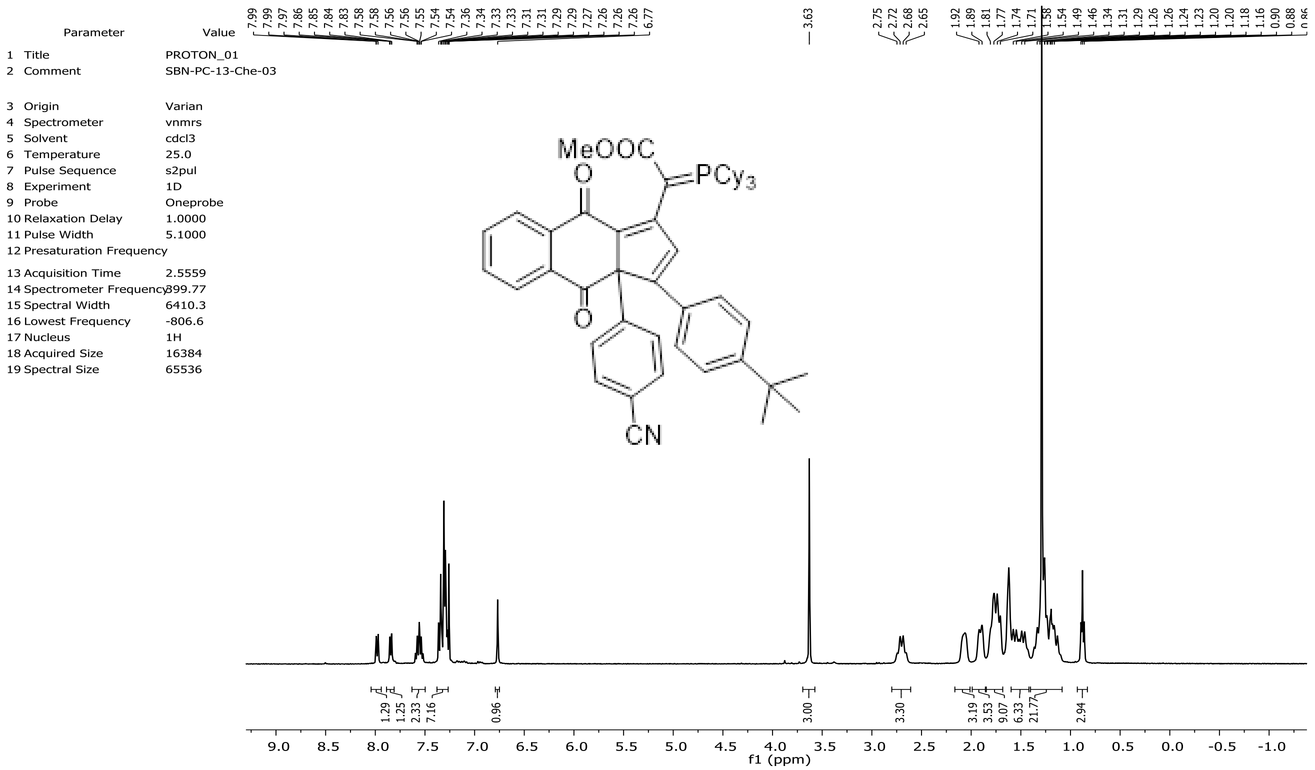


**Figure S30.** $^{13}$C-NMR spectrum of compound **3o** ($CDCl_3$, 100 MHz)

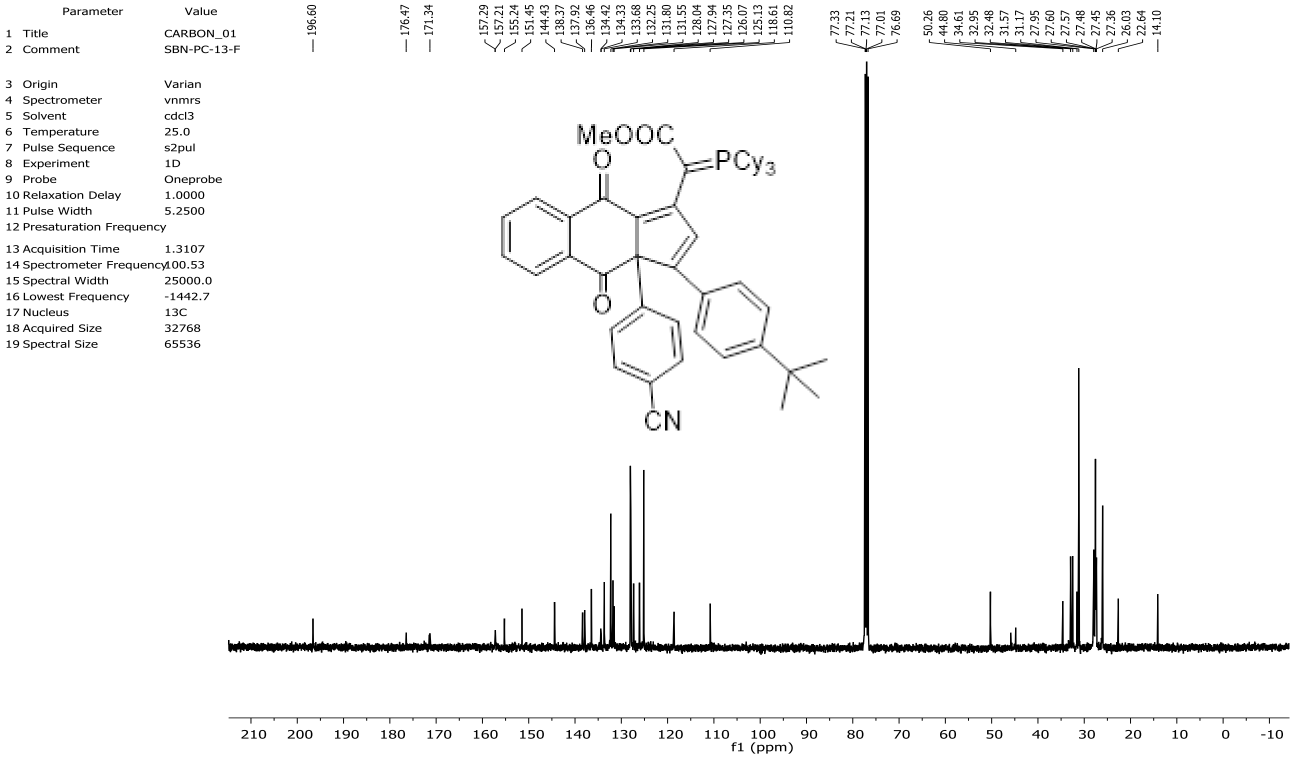

**Figure S31.** $^{1}$H-NMR spectrum of compound **3p** ($CDCl_3$, 400 MHz)

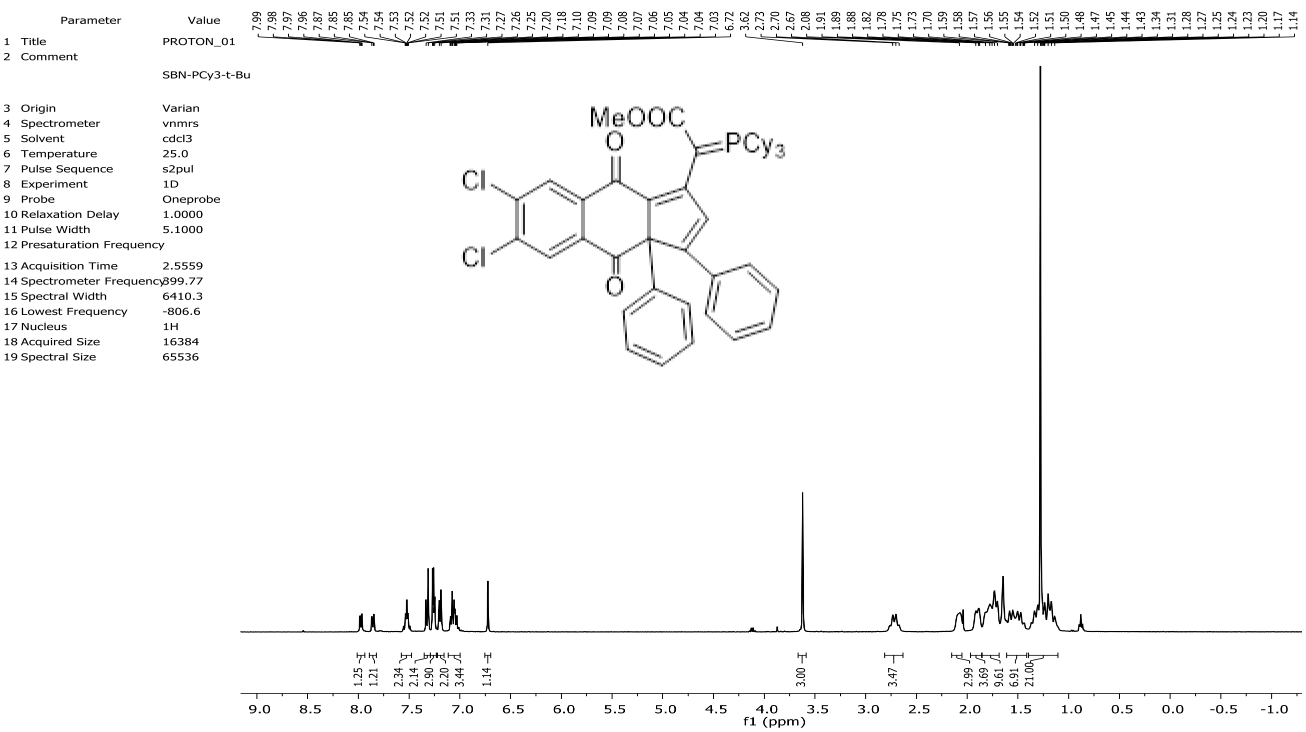


**Figure S32.** $^{13}$C-NMR spectrum of compound **3p** ($CDCl_3$, 100 MHz)

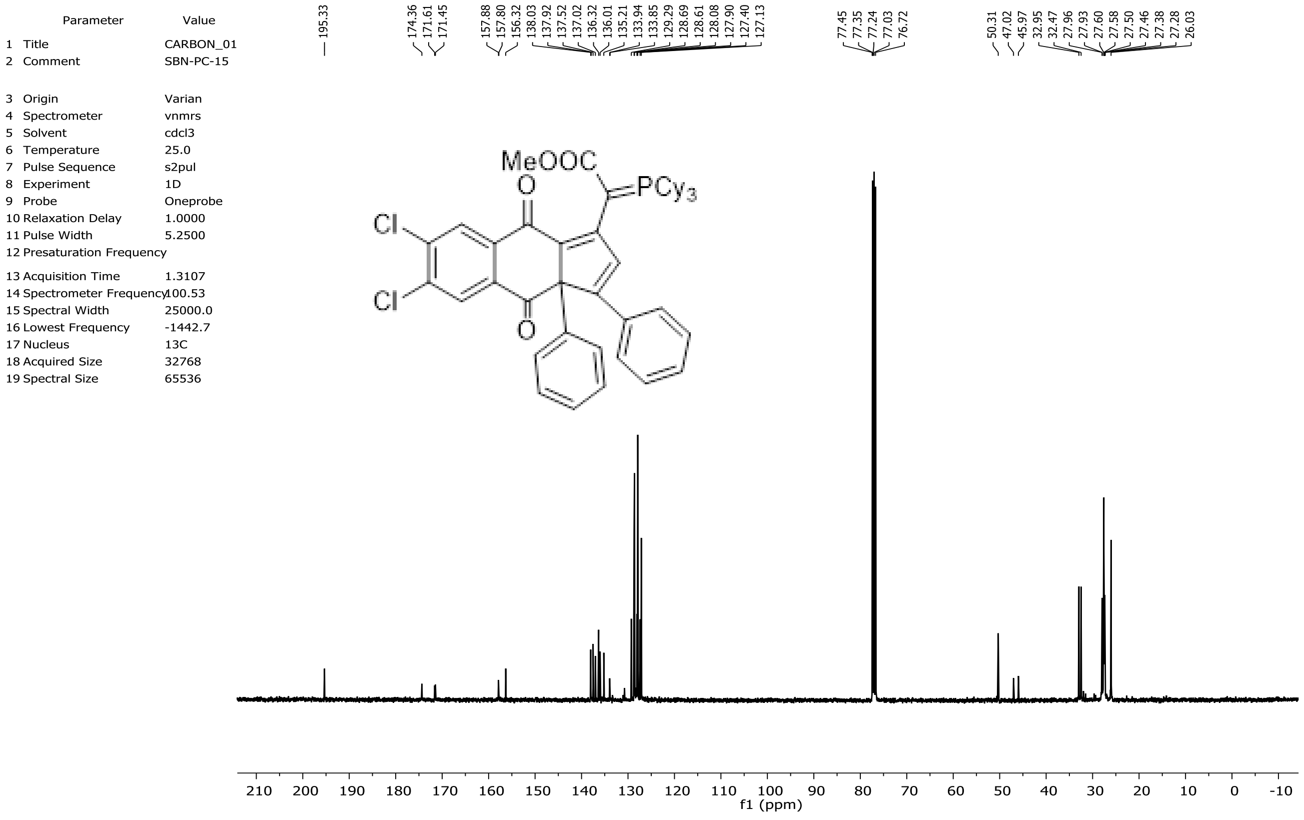

**Figure S33.** $^1$H-NMR spectrum of compound **4a** ($CDCl_3$, 400 MHz)

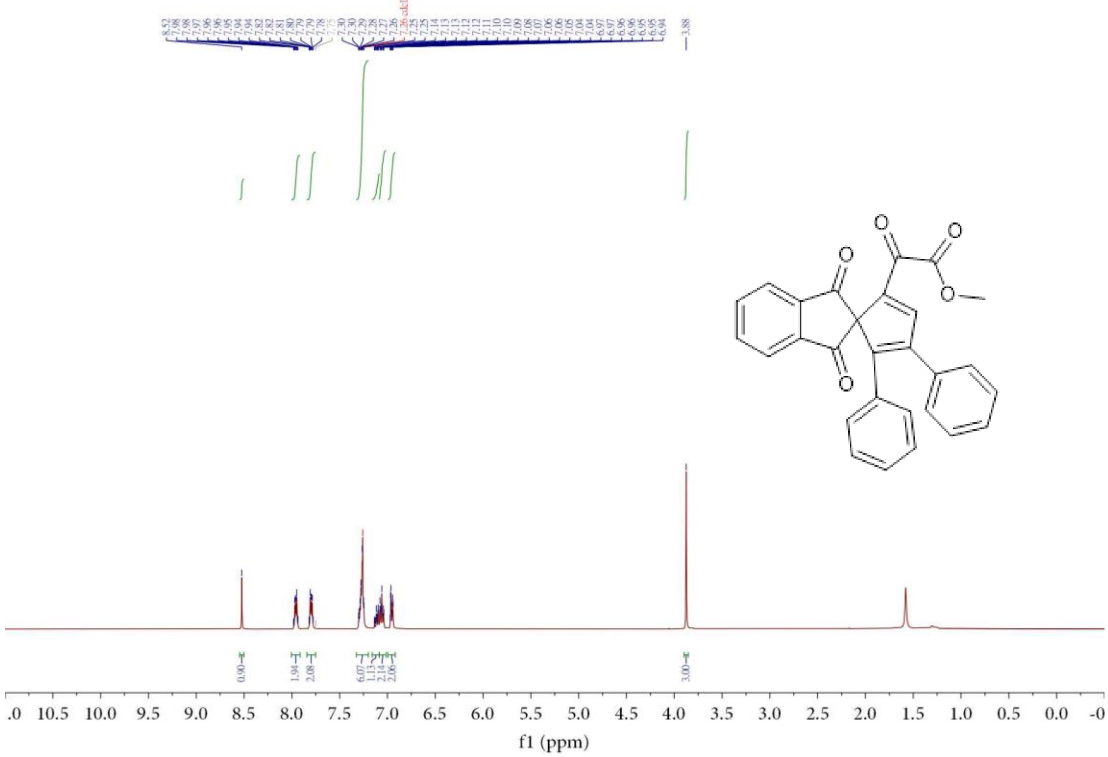

**Figure S34.** $^{13}$C-NMR spectrum of compound **4a** ($CDCl_3$, 100 MHz)

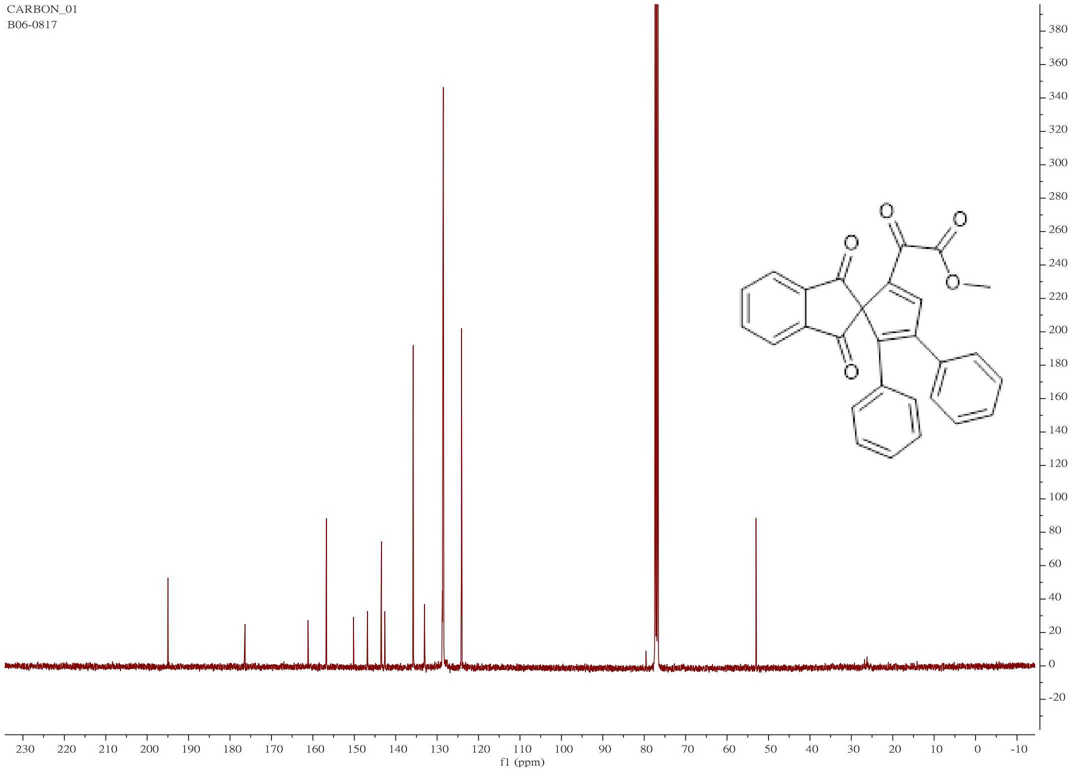

**Figure S35.** $^{1}$H-NMR spectrum of compound **4b** ($CDCl_3$, 400 MHz)

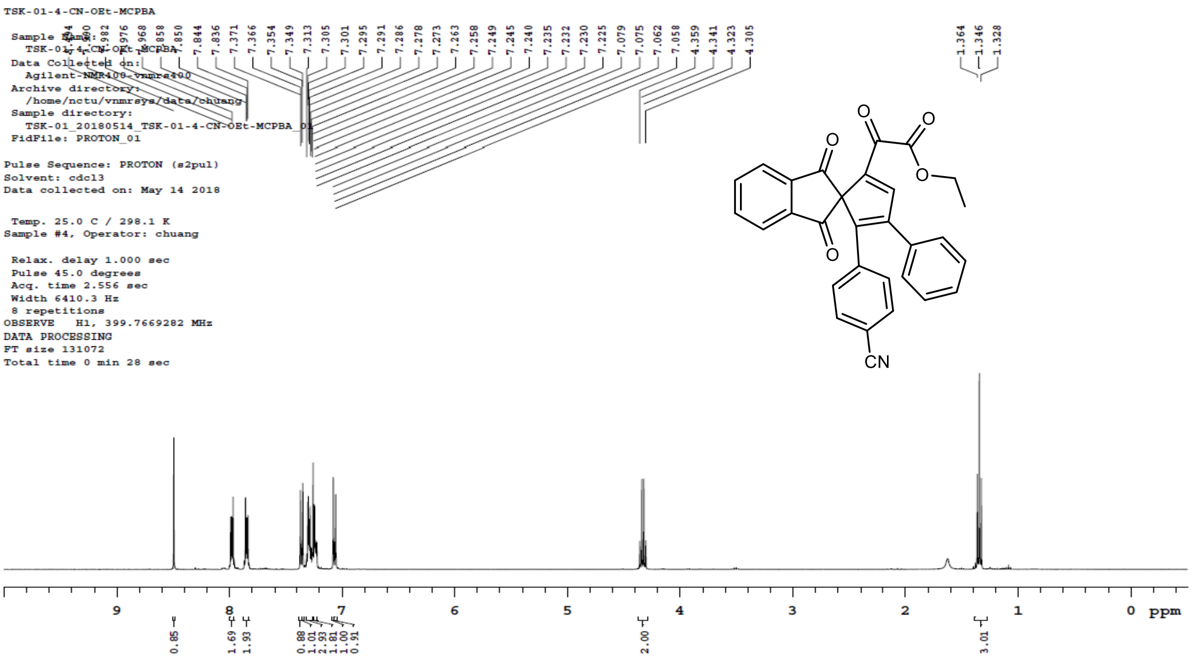


**Figure S36.** $^{13}$C-NMR spectrum of compound **4b** ($CDCl_3$, 100 MHz)

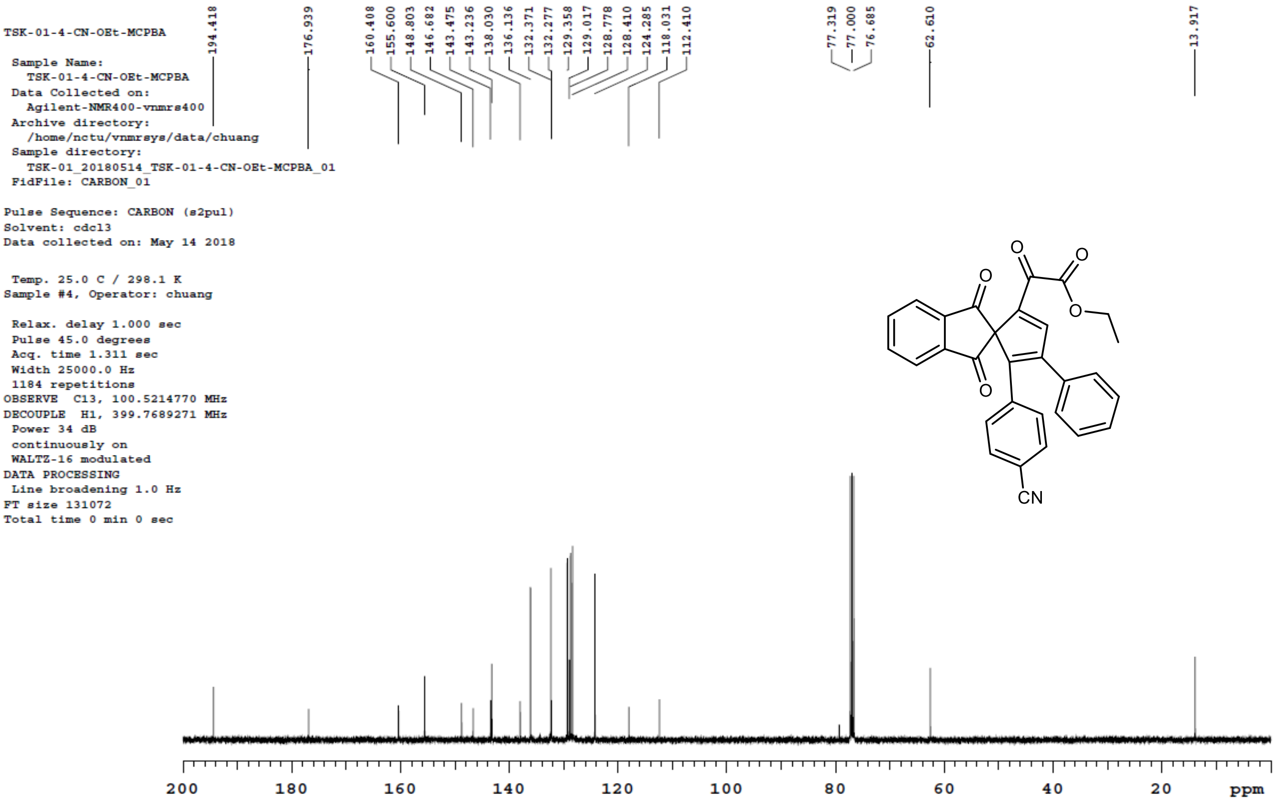

**Figure S37.** $^{1}$H-NMR spectrum of compound **4c** ($CDCl_3$, 400 MHz)

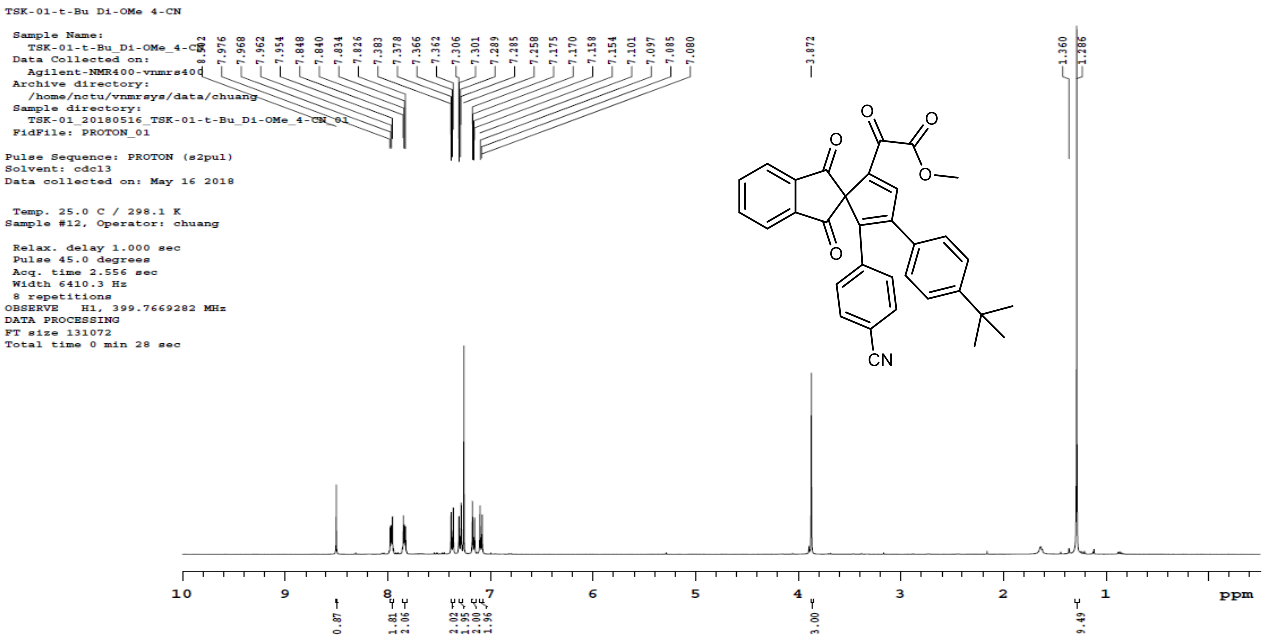


**Figure S38.** $^{13}$C-NMR spectrum of compound **4c** ($CDCl_3$, 100 MHz)

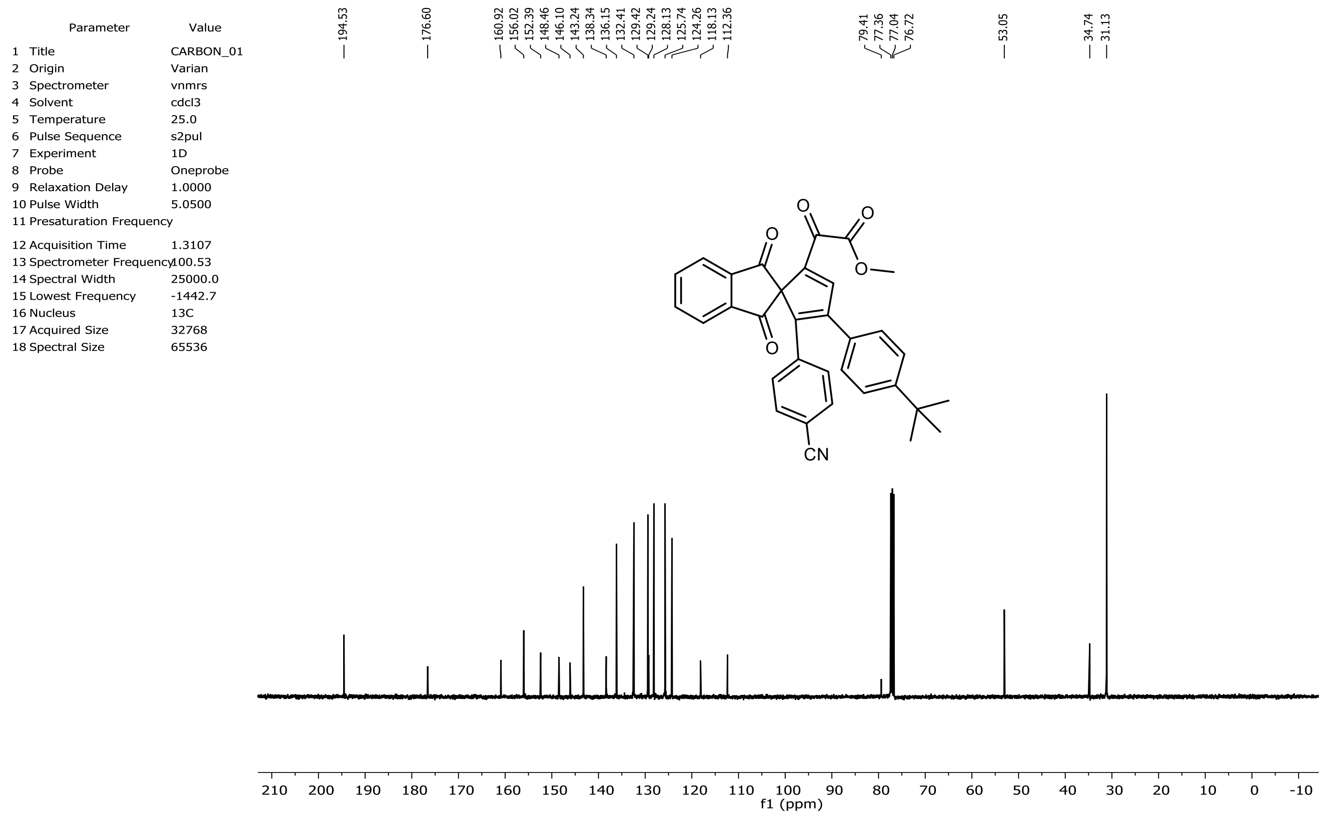

**Crystal growth method and XRD data for compounds 3h and 4a**

**Compound 3h:** Using 1.0 mL of the solvent mixture, [$CH_2Cl_2$: *n*-Hexane (1:1)], used to dissolve the compound and keep the solution in a vial at room temperature for slow evaporation to grow the crystal.

**Compound 4a:** Using 1.0 mL of the solvent mixture, [$CHCl_3$: *n*-Hexane (1:5)], used to dissolve the compound and keep the solution in a vial at room temperature for slow evaporation to grow the crystal.

## Table S1. Crystal data and experimental details for compound 3h (CCDC 2377008)

| | | |
|---|---|---|
| Empirical formula | $C_{47} H_{52} O_5 P$ | |
| Formula weight | 727.85 | |
| Temperature | 100(2) K | |
| Wavelength | 0.71073 Å | |
| Crystal system | Monoclinic | |
| Space group | P 21/c | |
| Unit cell dimensions | a = 12.8936(6) Å | α= 90°. |
| | b = 19.4787(9) Å | β= 103.421(2)°. |
| | c = 17.2463(7) Å | γ = 90°. |
| Volume | 4213.1(3) $Å^3$ | |
| Z | 4 | |
| Density (calculated) | 1.147 $Mg/m^3$ | |
| Absorption coefficient | 0.109 $mm^{-1}$ | |
| F(000) | 1556 | |
| Crystal size | 0.10 x 0.09 x 0.03 $mm^3$ | |
| Theta range for data collection | 1.602 to 26.413°. | |
| Index ranges | -16<=h<=15, -24<=k<=24, -21<=l<=21 | |
| Reflections collected | 34171 | |
| Independent reflections | 8623 [R(int) = 0.0445] | |
| Completeness to theta = 25.242° | 99.8 % | |
| Absorption correction | Semi-empirical from equivalents | |
| Max. and min. transmission | 0.9485 and 0.8771 | |
| Refinement method | Full-matrix least-squares on $F^2$ | |
| Data / restraints / parameters | 8623 / 0 / 480 | |
| Goodness-of-fit on $F^2$ | 1.059 | |
| Final R indices [I>2sigma(I)] | R1 = 0.0640, wR2 = 0.1595 | |
| R indices (all data) | R1 = 0.0888, wR2 = 0.1750 | |
| Extinction coefficient | n/a | |
| Largest diff. peak and hole | 1.859 and -0.873 e.$Å^{-3}$ | |

**Figure S39. Solid state structure of compound 3h** (Thermal ellipsoids are drawn at 50% probability)

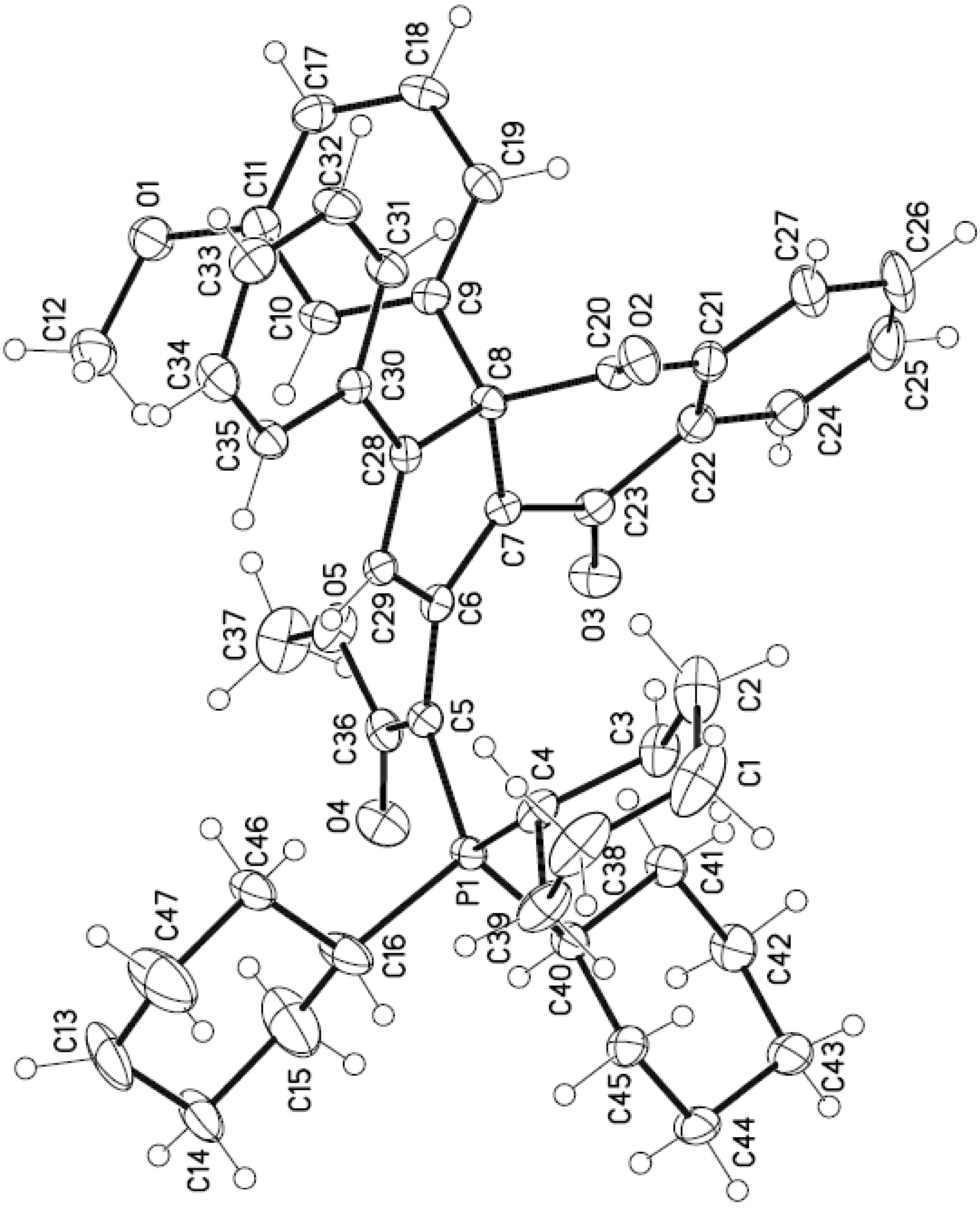

**Table S2. Crystal data and structure refinement for compound 4a (CCDC 2377009)**

| | |
|---|---|
| Identification code | 2403104lt3_auto |
| Empirical formula | $C_{28}H_{18}O_5$ |
| Formula weight | 434.42 |
| Temperature/K | 99.99(11) |
| Crystal system | orthorhombic |
| Space group | Pbca |
| a/Å | 21.8414(10) |
| b/Å | 8.2054(4) |
| c/Å | 23.0018(13) |
| α/° | 90 |
| β/° | 90 |
| γ/° | 90 |
| Volume/Å$^3$ | 4122.3(4) |
| Z | 8 |
| $\rho_{calc}$g/cm$^3$ | 1.400 |
| μ/mm$^{-1}$ | 0.786 |
| F(000) | 1808.0 |
| Crystal size/mm$^3$ | 0.11 × 0.08 × 0.01 |
| Radiation | Cu Kα (λ = 1.54184) |
| 2Θ range for data collection/° | 7.686 to 134.152 |
| Index ranges | $-26 \leq h \leq 23$, $-9 \leq k \leq 9$, $-20 \leq l \leq 27$ |
| Reflections collected | 10777 |
| Independent reflections | 3569 [$R_{int}$ = 0.0479, $R_{sigma}$ = 0.0350] |
| Data/restraints/parameters | 3569/0/299 |
| Goodness-of-fit on F$^2$ | 1.046 |
| Final R indexes [I>=2σ (I)] | $R_1$ = 0.1096, $wR_2$ = 0.3224 |
| Final R indexes [all data] | $R_1$ = 0.1380, $wR_2$ = 0.3446 |
| Largest diff. peak/hole / e Å$^{-3}$ | 0.53/-0.42 |

**Figure S40. Solid state structure of compound 4a** (Thermal ellipsoids are drawn at 50% probability)

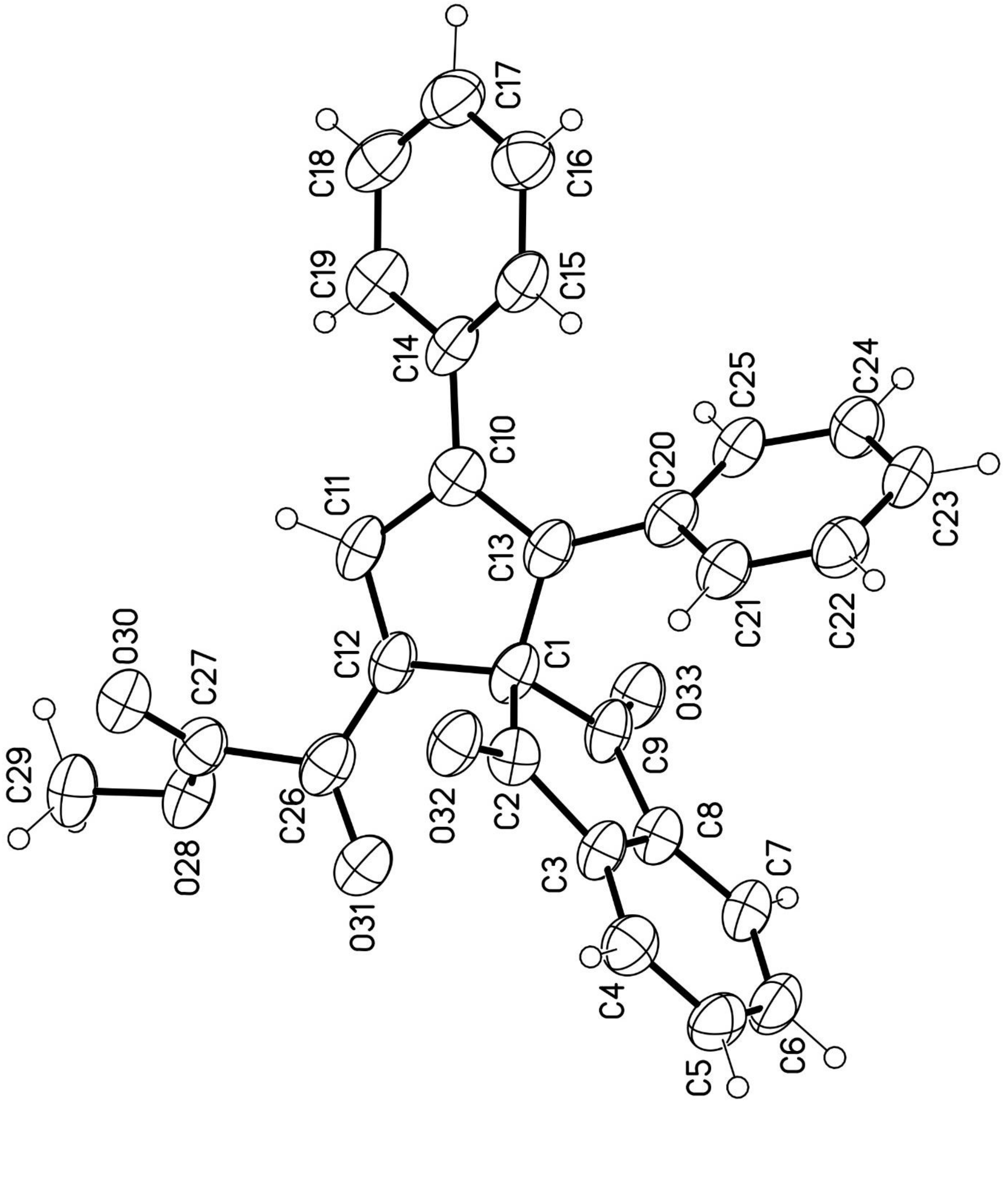

## Atomic coordinates of optimized structures

Atomic coordinates of optimized **3a** ($N_{imag}$ = 0)
G = -2425.145903 hartree
0 1

| | | | |
|---|---|---|---|
| C | 4.51717700 | -2.79052000 | 1.77262700 |
| C | 3.48762100 | -2.15528200 | 1.06712800 |
| C | 2.65291400 | -2.89763100 | 0.21349600 |
| C | 2.86848300 | -4.27120300 | 0.06713100 |
| C | 3.91580200 | -4.89268800 | 0.74270100 |
| C | 4.74038000 | -4.15337700 | 1.59926000 |
| H | 5.12933600 | -2.19985800 | 2.44613100 |
| H | 2.18987700 | -4.82735400 | -0.57066200 |
| H | 4.08697100 | -5.95769200 | 0.61359200 |
| H | 5.54986200 | -4.64301100 | 2.13260600 |
| C | 1.46044900 | -2.25855000 | -0.44638900 |
| C | 3.25672700 | -0.69447500 | 1.26714400 |
| O | 3.61291900 | -0.13828800 | 2.29455400 |
| O | 0.57434400 | -2.98392500 | -0.91844800 |
| C | 1.40703200 | -0.81571500 | -0.37319500 |
| C | 2.60584700 | 0.02798200 | 0.06911700 |
| C | 0.26299400 | -0.02593200 | -0.42318300 |
| C | 0.64103700 | 1.30472800 | 0.05594800 |
| H | -0.04058300 | 2.14738600 | 0.09578000 |
| C | 1.96452900 | 1.40082400 | 0.33838400 |
| C | 3.68186800 | 0.18440100 | -1.03836200 |
| C | 5.00871900 | 0.49269900 | -0.71240000 |
| C | 3.32602100 | 0.07032700 | -2.38918700 |
| C | 5.96338800 | 0.67411400 | -1.71443600 |
| H | 5.30360000 | 0.59585100 | 0.32665800 |
| C | 4.28051700 | 0.26091600 | -3.38796100 |
| H | 2.30137700 | -0.17127400 | -2.65317600 |
| C | 5.60339900 | 0.55908000 | -3.05631400 |
| H | 6.98875600 | 0.90667500 | -1.44134700 |
| H | 3.98821200 | 0.17030700 | -4.43051500 |
| H | 6.34601400 | 0.69908900 | -3.83654500 |
| C | -1.05706800 | -0.40770000 | -0.87240500 |
| C | -1.24163800 | -1.11430600 | -2.12584600 |

| | | | |
|---|---|---|---|
| O | -2.29790600 | -1.57521000 | -2.56932600 |
| O | -0.11353300 | -1.14329000 | -2.89249000 |
| C | -0.18931500 | -1.98443200 | -4.04610300 |
| H | -0.37880500 | -3.01951600 | -3.75213100 |
| H | -0.97956100 | -1.65475400 | -4.72570700 |
| H | 0.78523800 | -1.90551600 | -4.53040700 |
| P | -2.50232500 | 0.12345300 | 0.01304800 |
| C | 2.64423600 | 2.68475800 | 0.61132000 |
| C | 3.65549200 | 2.83506400 | 1.57747700 |
| C | 2.24451600 | 3.82732500 | -0.11095500 |
| C | 4.22803000 | 4.08349300 | 1.81805200 |
| H | 3.95871500 | 1.97139800 | 2.15516400 |
| C | 2.82036600 | 5.07202000 | 0.13078800 |
| H | 1.49578500 | 3.72455700 | -0.89026000 |
| C | 3.81615700 | 5.20651000 | 1.09965200 |
| H | 5.00018700 | 4.17765400 | 2.57658700 |
| H | 2.50016700 | 5.93438500 | -0.44723500 |
| H | 4.27057500 | 6.17515200 | 1.28719900 |
| C | -3.87003000 | -1.12844400 | -0.17857000 |
| C | -3.45906600 | -2.52745000 | 0.33348600 |
| C | -5.26889300 | -0.73530100 | 0.34217400 |
| H | -3.91459600 | -1.20952400 | -1.26894200 |
| C | -4.49594900 | -3.57158000 | -0.11031500 |
| H | -3.40299000 | -2.52277300 | 1.43033700 |
| H | -2.46883100 | -2.79279900 | -0.04559000 |
| C | -6.30110300 | -1.78762200 | -0.10581300 |
| H | -5.27673400 | -0.67656800 | 1.43561300 |
| H | -5.57016500 | 0.24923000 | -0.02924100 |
| C | -5.91195900 | -3.20038700 | 0.35277500 |
| H | -4.21432000 | -4.55705300 | 0.27844800 |
| H | -4.46985400 | -3.64592600 | -1.20516800 |
| H | -7.29051700 | -1.51806300 | 0.28283400 |
| H | -6.37771100 | -1.76913100 | -1.20152400 |
| H | -6.63978500 | -3.93045700 | -0.02043200 |
| H | -5.95871400 | -3.24766800 | 1.45046400 |
| C | -3.06544400 | 1.77226500 | -0.71716400 |
| C | -3.73083900 | 1.55656000 | -2.09835100 |
| C | -3.88342900 | 2.75635200 | 0.14511900 |

| | | | |
|---|---|---|---|
| H | -2.09136500 | 2.24540000 | -0.90571900 |
| C | -3.93066400 | 2.89499100 | -2.82703700 |
| H | -4.70974900 | 1.07997000 | -1.96097500 |
| H | -3.13721700 | 0.87256600 | -2.71064500 |
| C | -4.09369600 | 4.08792500 | -0.60045800 |
| H | -4.86132800 | 2.32565100 | 0.39356600 |
| H | -3.37266200 | 2.95701700 | 1.09213800 |
| C | -4.73868200 | 3.88500200 | -1.97772700 |
| H | -4.42930600 | 2.71759900 | -3.78683500 |
| H | -2.94894900 | 3.32955200 | -3.06153600 |
| H | -4.70506900 | 4.75771900 | 0.01559200 |
| H | -3.12042200 | 4.58284000 | -0.72588600 |
| H | -4.83235700 | 4.84644700 | -2.49575500 |
| H | -5.76018400 | 3.50039100 | -1.84695800 |
| C | -2.05419000 | 0.40565700 | 1.81546600 |
| C | -3.25216000 | 0.62568600 | 2.77092300 |
| C | -1.13996100 | -0.68839100 | 2.42172600 |
| H | -1.47474500 | 1.33889000 | 1.78601700 |
| C | -2.75637600 | 1.08505100 | 4.15379300 |
| H | -3.78970100 | -0.32216700 | 2.88948700 |
| H | -3.97005000 | 1.34754800 | 2.37679500 |
| C | -0.63134700 | -0.25268000 | 3.80661700 |
| H | -1.70978000 | -1.61985500 | 2.51844000 |
| H | -0.29442300 | -0.90604500 | 1.77073300 |
| C | -1.78155800 | 0.07201900 | 4.76893900 |
| H | -3.61618100 | 1.23988700 | 4.81633300 |
| H | -2.26025700 | 2.06066800 | 4.05325200 |
| H | 0.00262300 | -1.04290600 | 4.22420600 |
| H | 0.01371400 | 0.62851400 | 3.68773000 |
| H | -1.38950200 | 0.45130100 | 5.71968400 |
| H | -2.32830900 | -0.85232100 | 5.00333600 |

Atomic coordination of optimized **TS1** ($N_{img}$ = 1; $f_{imag}$ = -476.48)
G = -2425.099849 hartree
0 1

| | | | |
|---|---|---|---|
| C | -3.02281200 | -2.67537000 | -2.29309600 |
| C | -2.41521100 | -2.07929500 | -1.18816200 |
| C | -2.22959700 | -2.79028500 | 0.00003200 |
| C | -2.64438500 | -4.11560800 | 0.10930200 |

| | | | |
|---|---|---|---|
| C | -3.26670000 | -4.71351900 | -0.98750600 |
| C | -3.45352400 | -3.99850500 | -2.17885500 |
| H | -3.14738300 | -2.11746300 | -3.21534300 |
| H | -2.47062200 | -4.65553800 | 1.03440300 |
| H | -3.60302800 | -5.74408200 | -0.92344700 |
| H | -3.93053700 | -4.48324100 | -3.02557100 |
| C | -1.49744900 | -2.01376800 | 1.04719500 |
| C | -1.86071900 | -0.70206400 | -1.15107200 |
| O | -1.38362400 | -0.08362300 | -2.07562200 |
| O | -1.02739600 | -2.53371100 | 2.04600200 |
| C | -1.39090700 | -0.56955800 | 0.65820800 |
| C | -2.60854200 | 0.24021500 | 0.32279200 |
| C | -0.27132200 | 0.33178800 | 0.78624800 |
| C | -0.76799200 | 1.60335100 | 0.44413000 |
| H | -0.18324500 | 2.51395500 | 0.43872500 |
| C | -2.15015400 | 1.58739900 | 0.21084800 |
| C | -4.01869200 | -0.16535300 | 0.58002100 |
| C | -4.37420400 | -0.76807500 | 1.79759900 |
| C | -5.02526200 | 0.07411400 | -0.36920800 |
| C | -5.69959700 | -1.11686800 | 2.05842200 |
| H | -3.60801300 | -0.95531100 | 2.54401200 |
| C | -6.34921200 | -0.27274700 | -0.10626700 |
| H | -4.76309500 | 0.53699400 | -1.31564100 |
| C | -6.69187200 | -0.87035100 | 1.10849700 |
| H | -5.95581700 | -1.57993400 | 3.00706900 |
| H | -7.11390900 | -0.07992900 | -0.85332900 |
| H | -7.72327100 | -1.14337900 | 1.31167000 |
| C | 1.10133000 | -0.04118000 | 1.17575100 |
| C | 1.32124900 | -0.45094800 | 2.54369700 |
| O | 2.34758000 | -0.89834000 | 3.06335000 |
| O | 0.20419900 | -0.22955000 | 3.31068200 |
| C | 0.24308500 | -0.80155800 | 4.61719400 |
| H | 0.26435500 | -1.89354200 | 4.56008600 |
| H | 1.11642200 | -0.45614700 | 5.17628300 |
| H | -0.67510600 | -0.47451400 | 5.10900500 |
| P | 2.44065500 | 0.17950100 | 0.05042400 |
| C | -2.93663500 | 2.79217100 | -0.12296100 |
| C | -2.47731400 | 3.65758100 | -1.13165200 |
| C | -4.11169000 | 3.13570900 | 0.56791900 |
| C | -3.17160500 | 4.82551200 | -1.44361200 |
| H | -1.58144200 | 3.39073100 | -1.68399500 |

| | | | |
|---|---|---|---|
| C | -4.80197100 | 4.30592000 | 0.25723200 |
| H | -4.47256000 | 2.49051300 | 1.36113800 |
| C | -4.33698400 | 5.15447700 | -0.74962900 |
| H | -2.80425400 | 5.47654400 | -2.23199300 |
| H | -5.70335700 | 4.55876100 | 0.80827000 |
| H | -4.87826800 | 6.06493300 | -0.99021000 |
| C | 4.04621400 | -0.40316900 | 0.80504200 |
| C | 4.13094700 | -1.94430000 | 0.91030400 |
| C | 5.34006600 | 0.16797300 | 0.18283600 |
| H | 3.95659600 | -0.03484300 | 1.83252600 |
| C | 5.34970500 | -2.35489800 | 1.75042600 |
| H | 4.22587800 | -2.38266200 | -0.09137400 |
| H | 3.22215700 | -2.33120600 | 1.37279300 |
| C | 6.56328900 | -0.25833200 | 1.01645700 |
| H | 5.46342400 | -0.18926000 | -0.84653300 |
| H | 5.30901700 | 1.25940700 | 0.13724900 |
| C | 6.65266400 | -1.78144000 | 1.17832600 |
| H | 5.40581100 | -3.44866100 | 1.80345800 |
| H | 5.20036200 | -1.99747500 | 2.77714600 |
| H | 7.47574100 | 0.13288400 | 0.55033900 |
| H | 6.49428900 | 0.20724500 | 2.00927500 |
| H | 7.50243700 | -2.04259000 | 1.82022400 |
| H | 6.85125400 | -2.23781500 | 0.19764400 |
| C | 2.55262400 | 2.01841500 | -0.36014700 |
| C | 2.92757600 | 2.82184700 | 0.90679600 |
| C | 3.34073300 | 2.49994700 | -1.59825100 |
| H | 1.49960500 | 2.23144400 | -0.58034400 |
| C | 2.77290200 | 4.33455200 | 0.68300300 |
| H | 3.96779600 | 2.61164500 | 1.18698500 |
| H | 2.30449900 | 2.49778500 | 1.74664400 |
| C | 3.18191700 | 4.02058000 | -1.79052500 |
| H | 4.40578700 | 2.26051100 | -1.50515000 |
| H | 2.97699900 | 1.99610900 | -2.49793700 |
| C | 3.57474000 | 4.80954400 | -0.53546700 |
| H | 3.08754800 | 4.87265500 | 1.58472800 |
| H | 1.71056600 | 4.57377000 | 0.53347000 |
| H | 3.78141100 | 4.34193600 | -2.65054500 |
| H | 2.13485200 | 4.24135300 | -2.04157100 |
| H | 3.42175400 | 5.88261900 | -0.69936000 |
| H | 4.64811200 | 4.67330800 | -0.34023300 |
| C | 2.08690200 | -0.68577600 | -1.58190000 |

| | | | |
|---|---|---|---|
| C | 3.27932700 | -0.80855600 | -2.56075300 |
| C | 1.44860700 | -2.08114600 | -1.39474900 |
| H | 1.32105400 | -0.03985400 | -2.03871600 |
| C | 2.81227600 | -1.36190800 | -3.91910200 |
| H | 4.02055900 | -1.49750200 | -2.13795400 |
| H | 3.79081100 | 0.14415100 | -2.70987500 |
| C | 0.97211000 | -2.64502900 | -2.74366100 |
| H | 2.18311800 | -2.76479000 | -0.95395600 |
| H | 0.62016200 | -2.02866900 | -0.68816000 |
| C | 2.11445300 | -2.71981000 | -3.76599000 |
| H | 3.67185100 | -1.44689200 | -4.59495500 |
| H | 2.12000100 | -0.64412700 | -4.38063800 |
| H | 0.53422400 | -3.63825800 | -2.58987200 |
| H | 0.17139700 | -2.00253500 | -3.13059100 |
| H | 1.73708500 | -3.06361900 | -4.73638600 |
| H | 2.85087900 | -3.46623500 | -3.43538900 |

Atomic coordination of optimized **4aPCy₃** ($N_{imag}$ = 0)
G = -2425.135944 hartree
0 1

| | | | |
|---|---|---|---|
| C | 0.32973300 | 4.05704800 | -1.27877000 |
| C | 0.84246300 | 2.99748600 | -0.52713100 |
| C | 1.37515100 | 3.20541000 | 0.74703200 |
| C | 1.41301200 | 4.47947200 | 1.31290500 |
| C | 0.89146600 | 5.53840300 | 0.57100800 |
| C | 0.35647400 | 5.33022700 | -0.71314200 |
| H | -0.06805800 | 3.88153200 | -2.27302600 |
| H | 1.84074000 | 4.62684900 | 2.29910000 |
| H | 0.90175000 | 6.54279200 | 0.98416900 |
| H | -0.03472200 | 6.17693000 | -1.26930500 |
| C | 1.90775800 | 1.92987100 | 1.30310000 |
| C | 0.94522700 | 1.56489700 | -0.90213500 |
| O | 0.59111800 | 1.09030300 | -1.97022000 |
| O | 2.58044600 | 1.81846600 | 2.30201300 |
| C | 1.53467600 | 0.78964500 | 0.29112100 |
| C | 2.77394300 | -0.05863500 | 0.05244400 |
| C | 0.50611600 | -0.20258900 | 0.89201900 |
| C | 1.18550600 | -1.35370500 | 1.10463600 |
| H | 0.76000300 | -2.23928700 | 1.56145100 |
| C | 2.55838100 | -1.29284200 | 0.59464200 |
| C | 4.03728900 | 0.51590200 | -0.46174900 |
| C | 5.19648300 | 0.51399200 | 0.33457900 |

| | | | |
|---|---|---|---|
| C | 4.10435500 | 1.10009900 | -1.73915000 |
| C | 6.38467800 | 1.06940700 | -0.13536500 |
| H | 5.14765700 | 0.08808700 | 1.33088100 |
| C | 5.29576500 | 1.65524500 | -2.20730100 |
| H | 3.22471800 | 1.09660900 | -2.37477000 |
| C | 6.43952300 | 1.64174400 | -1.40817800 |
| H | 7.26783000 | 1.06284000 | 0.49717700 |
| H | 5.32983900 | 2.09483500 | -3.20027000 |
| H | 7.36612100 | 2.07581500 | -1.77278200 |
| C | -0.93294800 | 0.07738000 | 1.10344100 |
| C | -1.35421100 | 0.89564300 | 2.19796100 |
| O | -2.50688300 | 1.22896400 | 2.50006400 |
| O | -0.29147700 | 1.32986800 | 2.95695800 |
| C | -0.62290000 | 2.15859900 | 4.06973400 |
| H | -1.16961700 | 3.05176600 | 3.75288400 |
| H | -1.24036900 | 1.61834100 | 4.79357500 |
| H | 0.33186600 | 2.43450500 | 4.51986200 |
| P | -2.18610000 | -0.50167300 | -0.00988900 |
| C | 3.48500900 | -2.44125000 | 0.66888700 |
| C | 4.35685700 | -2.75730100 | -0.38815500 |
| C | 3.48371700 | -3.27393500 | 1.80187400 |
| C | 5.20330100 | -3.86087200 | -0.30798000 |
| H | 4.36028200 | -2.13660100 | -1.27756400 |
| C | 4.33470300 | -4.37512200 | 1.88250700 |
| H | 2.82410600 | -3.04153900 | 2.63277500 |
| C | 5.19795100 | -4.67399100 | 0.82747700 |
| H | 5.86586600 | -4.08973800 | -1.13789800 |
| H | 4.32356000 | -4.99940800 | 2.77149400 |
| H | 5.85848800 | -5.53406900 | 0.88770400 |
| C | -3.44424700 | -1.67406000 | 0.78323700 |
| C | -4.27476600 | -1.11289400 | 1.95973100 |
| C | -4.38625300 | -2.42583800 | -0.19063500 |
| H | -2.75028200 | -2.41532400 | 1.20627800 |
| C | -5.02581400 | -2.24533100 | 2.68168100 |
| H | -4.99822300 | -0.38133400 | 1.57499900 |
| H | -3.64141700 | -0.56778600 | 2.65639000 |
| C | -5.10423400 | -3.57829100 | 0.53402900 |
| H | -5.14322000 | -1.73558900 | -0.57430300 |
| H | -3.85097000 | -2.81704200 | -1.05890200 |
| C | -5.91005200 | -3.06630600 | 1.73479400 |
| H | -5.62852500 | -1.81990700 | 3.49276500 |

| | | | |
|---|---|---|---|
| H | -4.29213100 | -2.91072700 | 3.15926700 |
| H | -5.75908400 | -4.10322700 | -0.17205300 |
| H | -4.36074700 | -4.31254500 | 0.87534500 |
| H | -6.37241400 | -3.90456900 | 2.26962700 |
| H | -6.73380600 | -2.43620400 | 1.37018200 |
| C | -1.27544400 | -1.53379700 | -1.29870000 |
| C | -1.01229600 | -2.99638000 | -0.86812300 |
| C | -1.81581700 | -1.50843000 | -2.74708400 |
| H | -0.30791600 | -1.02213000 | -1.33283800 |
| C | -0.00194200 | -3.66612300 | -1.81513900 |
| H | -1.94571800 | -3.57060300 | -0.88940200 |
| H | -0.63865300 | -3.04045900 | 0.15638500 |
| C | -0.80614900 | -2.17668000 | -3.69825100 |
| H | -2.77537200 | -2.03699200 | -2.81326100 |
| H | -1.98847600 | -0.48422500 | -3.08596500 |
| C | -0.47721600 | -3.61355900 | -3.27303600 |
| H | 0.15861500 | -4.70491500 | -1.50315300 |
| H | 0.96720800 | -3.15898000 | -1.72321000 |
| H | -1.20376000 | -2.16247600 | -4.72021500 |
| H | 0.11245700 | -1.57554000 | -3.70846100 |
| H | 0.28303000 | -4.04140800 | -3.93707800 |
| H | -1.37519100 | -4.23870500 | -3.38367500 |
| C | -2.99857900 | 0.91129700 | -0.97485700 |
| C | -4.33738000 | 0.56911200 | -1.66442300 |
| C | -3.13060800 | 2.24532400 | -0.20895700 |
| H | -2.24156900 | 1.07840100 | -1.75498700 |
| C | -4.78053000 | 1.69869800 | -2.61094000 |
| H | -5.10814900 | 0.43238600 | -0.89571500 |
| H | -4.27475900 | -0.36762600 | -2.22334600 |
| C | -3.57473800 | 3.37280500 | -1.15587200 |
| H | -3.84469000 | 2.13848600 | 0.61211500 |
| H | -2.17675500 | 2.50751400 | 0.25094300 |
| C | -4.88672300 | 3.04090300 | -1.87742900 |
| H | -5.73990500 | 1.43414100 | -3.07183000 |
| H | -4.05422800 | 1.78644100 | -3.43148100 |
| H | -3.67731000 | 4.30392900 | -0.58598700 |
| H | -2.78775900 | 3.55135000 | -1.90300900 |
| H | -5.15368800 | 3.83949800 | -2.57995400 |
| H | -5.70090600 | 2.98614100 | -1.14113400 |

Atomic coordination of optimized **3aO** ($N_{imag}$ = 0)
G = -1453.609457 hartree

0 1

| | | | |
|---|---|---|---|
| C | -0.94495900 | 3.28225900 | -1.70272800 |
| C | -0.91122600 | 2.06320100 | -1.01631100 |
| C | -1.94020000 | 1.74463300 | -0.10782800 |
| C | -2.97897800 | 2.65369600 | 0.11451000 |
| C | -2.99168200 | 3.87498400 | -0.55405000 |
| C | -1.97729400 | 4.18681600 | -1.46677800 |
| H | -0.15466800 | 3.50220800 | -2.41234700 |
| H | -3.76596800 | 2.37457300 | 0.80699600 |
| H | -3.79495900 | 4.58303200 | -0.37401000 |
| H | -1.99523600 | 5.13545500 | -1.99464700 |
| C | -1.98266500 | 0.40128200 | 0.54336100 |
| C | 0.22275400 | 1.12055500 | -1.27757700 |
| O | 0.88019400 | 1.19408100 | -2.29895500 |
| O | -2.93428500 | 0.04244800 | 1.23431400 |
| C | -0.83299400 | -0.45882500 | 0.25269800 |
| C | 0.52337900 | 0.12447400 | -0.12434300 |
| C | -0.77507900 | -1.82111000 | 0.17732300 |
| C | 0.56645500 | -2.21908900 | -0.20769200 |
| H | 0.86447800 | -3.25458200 | -0.30769000 |
| C | 1.35627300 | -1.13336200 | -0.42924400 |
| C | 1.18470500 | 0.92017900 | 1.03066900 |
| C | 2.11582800 | 1.92991800 | 0.75882900 |
| C | 0.90303900 | 0.60033600 | 2.36544900 |
| C | 2.74508600 | 2.61258600 | 1.80094000 |
| H | 2.35943600 | 2.18609100 | -0.26663300 |
| C | 1.53653500 | 1.28066000 | 3.40441800 |
| H | 0.18719700 | -0.18186200 | 2.59568000 |
| C | 2.45751900 | 2.29192100 | 3.12672500 |
| H | 3.46193400 | 3.39520500 | 1.57084500 |
| H | 1.30534500 | 1.01932300 | 4.43276800 |
| H | 2.94605500 | 2.82453600 | 3.93706600 |
| C | -1.81985600 | -2.84182200 | 0.47199800 |
| C | -3.31353600 | -2.56222000 | 0.22402000 |
| O | -4.19466700 | -2.99640200 | 0.92140500 |
| O | -3.48337800 | -1.87130300 | -0.91839400 |
| C | -4.85308000 | -1.56146500 | -1.24077100 |
| H | -5.44876600 | -2.47514000 | -1.29509400 |
| H | -5.27004800 | -0.90249900 | -0.47591100 |
| H | -4.82190300 | -1.06136500 | -2.20802800 |
| C | 2.80209000 | -1.20899100 | -0.70401800 |

| | | | |
|---|---|---|---|
| C | 3.46948400 | -0.32734000 | -1.57371100 |
| C | 3.55385400 | -2.22754000 | -0.08323300 |
| C | 4.83430200 | -0.47099000 | -1.81846200 |
| H | 2.90213400 | 0.43900300 | -2.08555800 |
| C | 4.91668500 | -2.36532900 | -0.32893100 |
| H | 3.06463800 | -2.89637500 | 0.61772100 |
| C | 5.56414600 | -1.48607200 | -1.19928200 |
| H | 5.32780500 | 0.21285100 | -2.50309500 |
| H | 5.47526100 | -3.15337800 | 0.16750800 |
| H | 6.62834600 | -1.59000900 | -1.38958400 |
| O | -1.49969000 | -3.94096500 | 0.88264800 |

Atomic coordination of optimized **TS2** ($N_{imag}$ = 1; $f_{imag}$ = -417.92)
G = -1453.582019 hartree
0 1

| | | | |
|---|---|---|---|
| C | 1.39712300 | -2.80386900 | 2.27873400 |
| C | 0.67044600 | -2.15232700 | 1.27846300 |
| C | 0.22923600 | -2.84288800 | 0.14317200 |
| C | 0.51151200 | -4.19945300 | -0.00882500 |
| C | 1.24465100 | -4.85183500 | 0.98170700 |
| C | 1.68493500 | -4.15842400 | 2.11774800 |
| H | 1.72061800 | -2.25808500 | 3.15864300 |
| H | 0.14778600 | -4.71810900 | -0.88943900 |
| H | 1.47219200 | -5.90838400 | 0.87785700 |
| H | 2.24740500 | -4.68294100 | 2.88396900 |
| C | -0.56699200 | -2.04336900 | -0.83303900 |
| C | 0.26737900 | -0.73477300 | 1.37546900 |
| O | 0.05771900 | -0.06972800 | 2.34179400 |
| O | -1.04316600 | -2.50639200 | -1.85349000 |
| C | -0.64903600 | -0.60156900 | -0.43609200 |
| C | 0.55185800 | 0.24150400 | -0.29313700 |
| C | -1.78289000 | 0.22099000 | -0.25911300 |
| C | -1.31796200 | 1.54249000 | -0.02214800 |
| H | -1.96338700 | 2.38455000 | 0.18159700 |
| C | 0.07145500 | 1.58488600 | -0.06247200 |
| C | 1.91549000 | -0.11366600 | -0.77991800 |
| C | 3.05189300 | 0.19186400 | -0.01310900 |
| C | 2.08912900 | -0.73537500 | -2.02692200 |
| C | 4.32731000 | -0.11763700 | -0.48056500 |
| H | 2.93037900 | 0.67612700 | 0.95080600 |
| C | 3.36788000 | -1.04220600 | -2.49303100 |
| H | 1.22215800 | -0.97119400 | -2.63579600 |

| | | | |
|---|---|---|---|
| C | 4.48989500 | -0.73614400 | -1.72217600 |
| H | 5.19512400 | 0.12359900 | 0.12618200 |
| H | 3.48468400 | -1.51956400 | -3.46147600 |
| H | 5.48438900 | -0.97727000 | -2.08569900 |
| C | -3.13671600 | -0.33231600 | -0.17058700 |
| C | -4.32653300 | 0.65328500 | -0.23898400 |
| O | -4.24657500 | 1.85427600 | -0.07701200 |
| O | -5.46695600 | -0.00014500 | -0.48508800 |
| C | -6.64306500 | 0.82460900 | -0.55141100 |
| H | -6.80861200 | 1.33152600 | 0.40236800 |
| H | -6.54020900 | 1.57633500 | -1.33784900 |
| H | -7.46405100 | 0.14420200 | -0.77320900 |
| C | 0.87495400 | 2.80337300 | 0.17403200 |
| C | 0.60552800 | 3.60326300 | 1.29790200 |
| C | 1.87682000 | 3.21911800 | -0.71960800 |
| C | 1.31585800 | 4.78195600 | 1.52182400 |
| H | -0.15495400 | 3.28154500 | 2.00259800 |
| C | 2.58572100 | 4.39770700 | -0.49362100 |
| H | 2.08726500 | 2.62409700 | -1.60187700 |
| C | 2.30917200 | 5.18307100 | 0.62733000 |
| H | 1.09519100 | 5.38470400 | 2.39814700 |
| H | 3.35104300 | 4.70722800 | -1.19965400 |
| H | 2.86283100 | 6.10122800 | 0.80091700 |
| O | -3.35854200 | -1.52715700 | -0.04468200 |

Atomic coordination of optimized **4a** ($N_{imag} = 0$)
G = -1453.621078 hartree
0 1

| | | | |
|---|---|---|---|
| C | 4.03749000 | -1.20712700 | -1.32426200 |
| C | 2.90337200 | -0.75752900 | -0.64950900 |
| C | 2.81879100 | -0.81726700 | 0.74275700 |
| C | 3.86443900 | -1.32971400 | 1.50954200 |
| C | 5.00035100 | -1.78249900 | 0.83940000 |
| C | 5.08608300 | -1.72143500 | -0.56239500 |
| H | 4.09053700 | -1.14919100 | -2.40641700 |
| H | 3.78472200 | -1.36579100 | 2.59093300 |
| H | 5.83451600 | -2.18652400 | 1.40514300 |
| H | 5.98528300 | -2.07898900 | -1.05514100 |
| C | 1.52246000 | -0.27250900 | 1.21457800 |
| C | 1.66618000 | -0.17747500 | -1.22818600 |
| O | 1.43262600 | -0.00616100 | -2.40506700 |
| O | 1.15147800 | -0.17452100 | 2.36194300 |

| | | | |
|---|---|---|---|
| C | 0.68782700 | 0.14223600 | -0.05133600 |
| C | -0.63285200 | -0.60659700 | -0.08930400 |
| C | 0.25982700 | 1.59005000 | -0.00131700 |
| C | -1.09932400 | 1.63819200 | 0.03386700 |
| H | -1.69087200 | 2.54359100 | 0.05778500 |
| C | -1.66316000 | 0.29785300 | -0.01852800 |
| C | -0.71654300 | -2.08223400 | -0.05425700 |
| C | -0.12597400 | -2.87872000 | -1.05126800 |
| C | -1.37906000 | -2.71758600 | 1.01134800 |
| C | -0.20356600 | -4.26971400 | -0.98447000 |
| H | 0.36692900 | -2.40799200 | -1.89502500 |
| C | -1.44779400 | -4.10728700 | 1.07729900 |
| H | -1.82473800 | -2.11132900 | 1.79284100 |
| C | -0.86117900 | -4.88833000 | 0.07946000 |
| H | 0.24884600 | -4.86965500 | -1.76866500 |
| H | -1.95660000 | -4.58058300 | 1.91188500 |
| H | -0.91644900 | -5.97169500 | 0.13114200 |
| C | 1.24839700 | 2.65579000 | 0.03006400 |
| C | 0.81973100 | 4.13298500 | 0.15470600 |
| O | 1.43513500 | 4.94202800 | 0.80453700 |
| O | -0.27911800 | 4.41567700 | -0.57683300 |
| C | -0.68337600 | 5.79995300 | -0.55900800 |
| H | -0.94068400 | 6.10963000 | 0.45653700 |
| H | 0.12488000 | 6.43444700 | -0.92884100 |
| H | -1.55164300 | 5.85993000 | -1.21390000 |
| C | -3.11969800 | 0.04549900 | -0.02125700 |
| C | -3.69592100 | -0.93670200 | -0.84463700 |
| C | -3.96689000 | 0.82276700 | 0.78705300 |
| C | -5.07421400 | -1.13856300 | -0.85040400 |
| H | -3.05963200 | -1.53205000 | -1.49018300 |
| C | -5.34549500 | 0.61673200 | 0.78265500 |
| H | -3.53923800 | 1.58030100 | 1.43772600 |
| C | -5.90444800 | -0.36501600 | -0.03649000 |
| H | -5.50182900 | -1.89818500 | -1.49822600 |
| H | -5.98214700 | 1.22256900 | 1.42080800 |
| H | -6.97866200 | -0.52415400 | -0.04348800 |
| O | 2.45163500 | 2.42437000 | 0.00022900 |

Atomic coordination of optimized **3aOH** ($N_{imag}$ = 0)
G = -1453.955162 hartree
1 1

| | | | |
|---|---|---|---|
| C | -0.94150400 | 3.26108700 | -1.75549800 |
| C | -0.88816600 | 2.05448500 | -1.05132900 |
| C | -1.89887900 | 1.74527900 | -0.11306900 |
| C | -2.93981500 | 2.65043800 | 0.11761300 |
| C | -2.97269000 | 3.85949400 | -0.57390600 |
| C | -1.97986700 | 4.16008600 | -1.51373700 |
| H | -0.16713100 | 3.48216700 | -2.48181900 |
| H | -3.70727200 | 2.38959300 | 0.83830800 |
| H | -3.77388300 | 4.56726700 | -0.38759400 |
| H | -2.01720400 | 5.09722200 | -2.05963300 |
| C | -1.90490000 | 0.42944300 | 0.56778200 |
| C | 0.24497600 | 1.12283000 | -1.31429700 |
| O | 0.92250500 | 1.16466100 | -2.31311900 |
| O | -2.76872700 | 0.05641200 | 1.35540100 |
| C | -0.77842300 | -0.44656800 | 0.18555000 |
| C | 0.57079700 | 0.10347700 | -0.12844600 |
| C | -0.74588500 | -1.83184500 | 0.07076900 |
| C | 0.60957800 | -2.24415400 | -0.24603500 |
| H | 0.90895000 | -3.27617400 | -0.36814900 |
| C | 1.40666500 | -1.15203700 | -0.42873400 |
| C | 1.19062600 | 0.93837400 | 1.01934700 |
| C | 2.10038300 | 1.96631900 | 0.74687000 |
| C | 0.88416000 | 0.62507800 | 2.35107600 |
| C | 2.68933800 | 2.67502500 | 1.79445500 |
| H | 2.36308500 | 2.21579200 | -0.27515500 |
| C | 1.47516000 | 1.33706600 | 3.39360600 |
| H | 0.19284000 | -0.18004300 | 2.58133800 |
| C | 2.37717100 | 2.36559100 | 3.11833700 |
| H | 3.39470100 | 3.46898200 | 1.57102800 |
| H | 1.23041200 | 1.08420500 | 4.42033000 |
| H | 2.83600800 | 2.92001200 | 3.93053700 |
| C | -1.83017000 | -2.69530200 | 0.35197800 |
| C | -3.32234100 | -2.49521300 | 0.11236300 |
| O | -4.07277500 | -3.28795200 | 0.64971700 |
| O | -3.61583100 | -1.56414300 | -0.76376800 |
| C | -5.03618600 | -1.35287700 | -1.02005500 |
| H | -5.50727700 | -2.29990300 | -1.28468400 |
| H | -5.49815400 | -0.93654300 | -0.12361800 |
| H | -5.07124400 | -0.64774300 | -1.84682200 |
| C | 2.85118400 | -1.22291000 | -0.68045700 |
| C | 3.53600300 | -0.30974200 | -1.50444700 |

| | | | |
|---|---|---|---|
| C | 3.58438900 | -2.27060600 | -0.08326600 |
| C | 4.90305000 | -0.45210200 | -1.72986500 |
| H | 2.98794500 | 0.47733100 | -2.00439300 |
| C | 4.95042400 | -2.40254900 | -0.30611400 |
| H | 3.08309100 | -2.96150000 | 0.58710100 |
| C | 5.61499200 | -1.49350500 | -1.13271300 |
| H | 5.41288400 | 0.25133000 | -2.38056900 |
| H | 5.49855200 | -3.20809100 | 0.17198000 |
| H | 6.68152300 | -1.59521400 | -1.30703900 |
| O | -1.57727500 | -3.87914700 | 0.81447700 |
| H | -2.45141100 | -4.31532900 | 0.99051700 |

Atomic coordination of optimized **TS3** ($N_{imag}$ = 1; $f_{imag}$ = -216.71)
G = -1453.948462 hartree

1 1

| | | | |
|---|---|---|---|
| C | -1.17483000 | 2.76785700 | -2.11507900 |
| C | -1.07345900 | 1.69946600 | -1.21565100 |
| C | -1.89780500 | 1.62889200 | -0.07759700 |
| C | -2.83200300 | 2.63505800 | 0.16280800 |
| C | -2.92566000 | 3.70942100 | -0.72433900 |
| C | -2.10528600 | 3.77378200 | -1.85764400 |
| H | -0.53650800 | 2.80405800 | -2.99113500 |
| H | -3.46751500 | 2.56448700 | 1.03897500 |
| H | -3.64374800 | 4.50111900 | -0.53633600 |
| H | -2.19600700 | 4.60870000 | -2.54453900 |
| C | -1.78879900 | 0.43374000 | 0.80371100 |
| C | -0.12329900 | 0.61333300 | -1.45800700 |
| O | 0.38004400 | 0.18669600 | -2.43875800 |
| O | -2.50855800 | 0.22961600 | 1.76594300 |
| C | -0.69982100 | -0.50425300 | 0.39876800 |
| C | 0.65440400 | -0.04973400 | 0.18739200 |
| C | -0.71913900 | -1.92521600 | 0.24221900 |
| C | 0.64740200 | -2.34927700 | 0.00089800 |
| H | 0.94065700 | -3.37846800 | -0.15775800 |
| C | 1.47188700 | -1.26678500 | -0.08538500 |
| C | 1.23396300 | 1.16546900 | 0.83950400 |
| C | 2.20653400 | 1.95091400 | 0.19953300 |
| C | 0.83728500 | 1.50053000 | 2.14678300 |
| C | 2.76150300 | 3.05096200 | 0.84946300 |
| H | 2.53302500 | 1.70467700 | -0.80390200 |
| C | 1.40270600 | 2.59673500 | 2.79453000 |

| | | | |
|---|---|---|---|
| H | 0.11144800 | 0.88694200 | 2.67043300 |
| C | 2.36173900 | 3.37748000 | 2.14675800 |
| H | 3.51002400 | 3.65119700 | 0.34240400 |
| H | 1.09906900 | 2.83407600 | 3.80908000 |
| H | 2.80060500 | 4.23140000 | 2.65278000 |
| C | -1.79630800 | -2.79830300 | 0.31697500 |
| C | -3.28447700 | -2.51937400 | 0.22032100 |
| O | -4.04975900 | -3.40195400 | 0.55983400 |
| O | -3.60063700 | -1.36285600 | -0.32722700 |
| C | -5.02541000 | -1.07410900 | -0.41888600 |
| H | -5.52719500 | -1.87111500 | -0.96845400 |
| H | -5.44212400 | -0.98958900 | 0.58564400 |
| H | -5.08817900 | -0.12792700 | -0.95113600 |
| C | 2.92889500 | -1.33215000 | -0.32414500 |
| C | 3.52510900 | -0.72469100 | -1.44194700 |
| C | 3.73332500 | -2.07174600 | 0.55843800 |
| C | 4.89563900 | -0.84762200 | -1.66237800 |
| H | 2.90894600 | -0.19583700 | -2.16173700 |
| C | 5.10515100 | -2.18548300 | 0.33816700 |
| H | 3.28137700 | -2.54191500 | 1.42654900 |
| C | 5.68933800 | -1.57246500 | -0.77088700 |
| H | 5.34283200 | -0.38562900 | -2.53703600 |
| H | 5.71619700 | -2.75268000 | 1.03317500 |
| H | 6.75704400 | -1.66346800 | -0.94362000 |
| O | -1.56080300 | -4.08209800 | 0.45939000 |
| H | -2.43373900 | -4.52984900 | 0.55722200 |

Atomic coordination of optimized **4aH** ($N_{img}$ = 0)
G = -1453.99502 hartree
1 1

| | | | |
|---|---|---|---|
| C | -3.92276100 | -1.35948300 | 1.55359700 |
| C | -2.83931500 | -0.91258000 | 0.79629200 |
| C | -2.84642700 | -0.99287400 | -0.60154300 |
| C | -3.93686400 | -1.52557000 | -1.28864900 |
| C | -5.02095200 | -1.97720100 | -0.53477100 |
| C | -5.01434200 | -1.89475700 | 0.86867500 |
| H | -3.90695700 | -1.28873800 | 2.63602300 |
| H | -3.93248800 | -1.58004800 | -2.37203100 |
| H | -5.88620900 | -2.39787600 | -1.03716200 |
| H | -5.87415000 | -2.25342800 | 1.42552100 |
| C | -1.60267900 | -0.43660100 | -1.17562100 |
| C | -1.58095200 | -0.31712500 | 1.29284300 |

| | | | |
|---|---|---|---|
| O | -1.25383400 | -0.10147000 | 2.43341300 |
| O | -1.30650500 | -0.32383600 | -2.34000200 |
| C | -0.68474000 | 0.02748800 | 0.02468200 |
| C | 0.67226800 | -0.62927200 | 0.00789700 |
| C | -0.34687000 | 1.50459000 | -0.01381600 |
| C | 1.05560000 | 1.62427200 | 0.02859900 |
| H | 1.58709500 | 2.56500700 | 0.06795600 |
| C | 1.67741300 | 0.36856200 | 0.05513700 |
| C | 0.88026000 | -2.05556200 | -0.14618500 |
| C | -0.02039200 | -3.00775200 | 0.38948900 |
| C | 1.98404500 | -2.52696600 | -0.90171500 |
| C | 0.18862900 | -4.36687600 | 0.19878600 |
| H | -0.85761400 | -2.68976700 | 0.99706000 |
| C | 2.16430800 | -3.88564500 | -1.11793600 |
| H | 2.66944800 | -1.81867800 | -1.34894200 |
| C | 1.27560400 | -4.81051400 | -0.56098400 |
| H | -0.49554200 | -5.08344000 | 0.64085000 |
| H | 2.99892200 | -4.22692200 | -1.72117200 |
| H | 1.42839800 | -5.87341100 | -0.71928200 |
| C | -1.27620000 | 2.51608300 | -0.04854000 |
| C | -1.05058900 | 4.01127300 | -0.12338300 |
| O | -2.03549300 | 4.72790700 | -0.14088500 |
| O | 0.20811200 | 4.40991700 | -0.16289900 |
| C | 0.41440100 | 5.85156400 | -0.22950100 |
| H | -0.05405600 | 6.24708600 | -1.13147900 |
| H | -0.01763400 | 6.32455900 | 0.65319100 |
| H | 1.49366800 | 5.98181200 | -0.25690100 |
| C | 3.14300400 | 0.20624000 | 0.17378200 |
| C | 3.69874700 | -0.62945500 | 1.15751300 |
| C | 3.99806400 | 0.94550900 | -0.65990200 |
| C | 5.08102600 | -0.72228000 | 1.29859300 |
| H | 3.04786500 | -1.18910200 | 1.82157600 |
| C | 5.38144300 | 0.84058400 | -0.52136300 |
| H | 3.57926200 | 1.58553000 | -1.43161800 |
| C | 5.92486300 | 0.00839200 | 0.45840400 |
| H | 5.50038500 | -1.36122200 | 2.06920000 |
| H | 6.03295700 | 1.40761900 | -1.17856300 |
| H | 7.00174600 | -0.06942100 | 0.56901900 |
| O | -2.56791400 | 2.22627700 | -0.02754700 |
| H | -3.04958800 | 3.08581800 | -0.06216100 |
